# Integrated multi-omics analyses identify anti-viral host factors and pathways controlling SARS-CoV-2 infection

**DOI:** 10.1038/s41467-023-44175-1

**Published:** 2024-01-02

**Authors:** Jiakai Hou, Yanjun Wei, Jing Zou, Roshni Jaffery, Long Sun, Shaoheng Liang, Ningbo Zheng, Ashley M. Guerrero, Nicholas A. Egan, Ritu Bohat, Si Chen, Caishang Zheng, Xiaobo Mao, S. Stephen Yi, Ken Chen, Daniel J. McGrail, Nidhi Sahni, Pei-Yong Shi, Yiwen Chen, Xuping Xie, Weiyi Peng

**Affiliations:** 1 Department of Biology and Biochemistry, University of Houston, Houston, TX USA; 2 Department of Bioinformatics and Computational Biology, The University of Texas MD Anderson Cancer Center, Houston, TX USA; 3 Department of Biochemistry & Molecular Biology, The University of Texas Medical Branch, Galveston, TX USA; 4 Department of Computer Science, Rice University, Houston, TX USA; 5 Neuroregeneration and Stem Cell Programs, Institute for Cell Engineering, Department of Neurology, Johns Hopkins University School of Medicine, Baltimore, MD USA; 6 Department of Oncology, Livestrong Cancer Institutes, and Department of Biomedical Engineering, The University of Texas at Austin, Austin, TX USA; 7 Interdisciplinary Life Sciences Graduate Programs (ILSGP) and Oden Institute for Computational Engineering and Sciences (ICES), The University of Texas at Austin, Austin, TX USA; 8 Center for Immunotherapy and Precision Immuno-Oncology, Cleveland Clinic, Cleveland, OH USA; 9 Department of Epigenetics and Molecular Carcinogenesis, The University of Texas MD Anderson Cancer Center, Houston, TX USA; 10 Institute for Human Infections and Immunity, The University of Texas Medical Branch, Galveston, TX USA; 11 Sealy Institute for Vaccine Sciences, The University of Texas Medical Branch, Galveston, TX USA; 12 Sealy Center for Structural Biology & Molecular Biophysics, The University of Texas Medical Branch, Galveston, TX USA; 13 Institute for Translational Science, The University of Texas Medical Branch, Galveston, TX USA; 14 Sealy Institute for Drug Discovery, The University of Texas Medical Branch, Galveston, TX USA; 15 Quantitative Sciences Program, MD Anderson Cancer Center, UT Health Graduate School of Biomedical Sciences, Houston, TX USA; 16 Present Address: Computational Biology Department, School of Computer Science, Carnegie Mellon University, Pittsburgh, PA USA

**Keywords:** SARS-CoV-2, High-throughput screening, Viral infection, Antimicrobial responses

## Abstract

Host anti-viral factors are essential for controlling SARS-CoV-2 infection but remain largely unknown due to the biases of previous large-scale studies toward pro-viral host factors. To fill in this knowledge gap, we perform a genome-wide CRISPR dropout screen and integrate analyses of the multi-omics data of the CRISPR screen, genome-wide association studies, single-cell RNA-Seq, and host-virus proteins or protein/RNA interactome. This study uncovers many host factors that are currently underappreciated, including the components of V-ATPases, ESCRT, and N-glycosylation pathways that modulate viral entry and/or replication. The cohesin complex is also identified as an anti-viral pathway, suggesting an important role of three-dimensional chromatin organization in mediating host-viral interaction. Furthermore, we discover another anti-viral regulator KLF5, a transcriptional factor involved in sphingolipid metabolism, which is up-regulated, and harbors genetic variations linked to COVID-19 patients with severe symptoms. Anti-viral effects of three identified candidates (DAZAP2/VTA1/KLF5) are confirmed individually. Molecular characterization of DAZAP2/VTA1/KLF5-knockout cells highlights the involvement of genes related to the coagulation system in determining the severity of COVID-19. Together, our results provide further resources for understanding the host anti-viral network during SARS-CoV-2 infection and may help develop new countermeasure strategies.

## Abstract (web summary)

Amnesic screening methods are useful to discover host factors that are important for SARS-CoV2 infection. Here the authors use a CRISPR screen to identify three anti-viral factors, which are associated with the coagulation system, and two pro-viral candidates and then use individual genetic deletion experiments to characterise their effect.

## Introduction

The 2019 coronavirus disease (COVID-19) pandemic, caused by severe acute respiratory syndrome coronavirus 2 (SARS-CoV-2), has already claimed over six million lives and resulted in global economic disruption^1^. As a newly emerged coronavirus, SARS-CoV-2 is an enveloped, single-stranded, positive-sense RNA virus^2^. Similar to other coronaviruses, SARS-CoV-2 hijacks a broad range of host factors to complete its infection cycle, including viral entry, replication, virion assembly, and dissemination. Although COVID-19 vaccines have successfully prevented severe disease and death^3^, SARS-CoV-2 variants with increased transmissibility and immune evasion continue to emerge, leading to the prolonged pandemic and breakthrough infections. A better understanding of virus-host interactions will provide new strategies for countermeasure development.

Unbiased virus-host interactome screens and genetic screens have advanced our understanding of SARS-CoV-2 biology. In virus-host interactome screens, cellular proteins that interact with viral proteins are pulled down by affinity purification (AP) using tagged viral proteins, followed by mass spectrometry (MS) to determine the identities of the interacting proteins. This approach has identified hundreds of SARS-CoV-2 interacting proteins that are involved in epigenetic regulation, mRNA translation machinery, protein post-translational modifications, and innate immune responses^4,5^. Some viral interacting proteins, such as Sigma receptors, display in vitro anti-viral activity^6^. In genetic screens, the impact of CRISPR guide RNA (gRNA)-mediated perturbation of an individual host gene in response to viral infection-mediated cytopathic effects (CPEs) is evaluated. Genes whose expression significantly affects CPEs are identified as pro-viral or anti-viral host factors. For SARS-CoV-2, both loss-of-function (LOF) and gain-of-function (GOF) screens have been performed by using the CRISPR knockout (KO) system^7–15^ and the CRISPR activation system^14,16,17^. These studies confirm the prominent roles of several known host factors in SARS-CoV-2 infection, such as ACE2 and TMEM30A as receptors to bind viral spike proteins, and TMPRSS2 and cathepsin L as key proteases for viral entry. More importantly, these screens provide a wealth of information on further pro-viral host factors including KCNA6, TMEM41B, TMEM106B, HMGB1, class III PI3K subunits, and proteins in the SWI/SNF chromatin remodeling complex.

However, recently reported CRISPR genetic screens are largely biased for identifying pro-viral host factors for SARS-CoV-2. Despite genome-wide, bidirectional CRISPR KO screens have been conducted by two independent groups^14,15^, all loss-of-function screens were under the condition of low multiplicities of infection (MOI) (range from 0.01 to 1) of virus for a long period of selection (ranging from 7 to 14 days post-infection). Under this condition, host cells are selected by many rounds of viral infection cycles, which could bias to identify host factors that regulate cell growth under the infection condition. Another weakness of this long selection screen is the loss of high complexities of the gRNA library in the pooled samples at the interrogation time point(s) when viral selection lasts more than 7 days. As gRNAs targeting potential pro-viral factors should be enriched in pooled samples, the library complexity in the pooled sample is not critical for the identification of these factors. However, keeping high library complexity is important to capture anti-viral host factors, whose gRNAs should be underrepresented in pooled samples.

In this study, we perform the genome-wide CRISPR dropout screen opting for a selection condition of a 2-day SARS-CoV-2 infection at MOI = 5. This selection condition achieves a delicate balance by preserving library complexity while capturing host factors directly regulating viral infection, thus enhancing the chance to identify physiologically relevant anti-viral host factors. The identified host factors are further analyzed for their clinical relevance by integrating databases of virus-host interactome, genome-wide association analysis (GWAS), and single-cell transcriptome of COVID-19 patients. The top 30 ranked hits (4 of pro-viral factors and 26 of anti-viral factors) are individually validated for their phenotypes observed in our genome-wide dropout screen. Among the validated hits, we characterize the role of two pro-viral factors (*ATP6V0D1* and *DPAGT1*) and three anti-viral factors (*DAZAP2*, *VTA1*, and *KLF5*) in regulating viral replication for mechanistic insights. Taken together, our study has broadened the understanding of the virus-host interaction during SARS-CoV-2 infection and has identified host risk factors associated with COVID-19 severity.

## Results

### Genome-wide CRISPR dropout screens identify host factors controlling vulnerability to SARS-CoV-2 infection

**Fig. 1:**
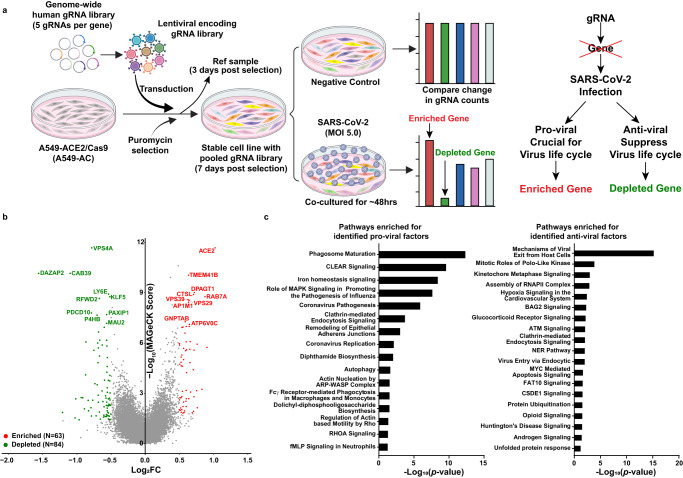
Discovery of host factors controlling SARS-CoV-2 infection. **a** A schematic diagram of the functional CRISPR/Cas9 dropout screen based on virus-induced cytopathic effect (CPE). A549-AC cells were transduced with a genome-wide human gRNA library (five gRNAs per gene) and followed by puromycin selection. After 3-day puromycin selection, 30 million pooled cells were collected as the reference sample. On day 7 after selection, pooled A549-AC cells were infected with recombinant SARS-CoV-2 at MOI = 5 for 48 h. Pooled A549-AC cells without viral treatment were severed as the controls. The changes in gRNA distribution between the virus-infected samples and controls were determined. **b** A volcano plot showing top candidates for pro-viral and anti-viral host factors. The gene-level MAGeCK scores and the changes in gRNA distribution between A549-AC cells with and without viral infection were calculated. The log_2_ fold change of the second-best gRNA for each gene was selected for data representation. Genes whose gRNAs were significantly enriched or depleted in the infected group (*P* value < 0.05 and |log_2_FC | ≥0.5) were labeled as red and green dots, respectively. The top ten enriched/depleted (pro-viral/anti-viral) genes based on MAGeCK scores were indicated. *P* values were calculated from the negative-binomial model. Two one-sided *P* values were provided to test whether gRNA was positively or negatively selected. Adjusted *P* value was calculated by using the Benjamini–Hochberg procedure. **c** Ingenuity Pathway Analysis of identified host factors for SARS-CoV-2 infection. Enriched pathways for pro-viral factors (enriched, left panel) and anti-viral factors (depleted, right panel) with statistical significance (*P* value < 0.05) were illustrated. *P* values for each gene set were calculated by using a Right-Tailed Fisher’s Exact Test and exact *P* values were provided in the source data file.

Here, we performed a genome-wide LOF screen based on the virus-induced cytopathic effect. Among several SARS-CoV-2-permissive epithelial cell lines, the A549 cell line is derived from a type II pneumocyte human lung adenocarcinoma and is commonly used for studying respiratory infections. To minimize the variability in response to SARS-CoV-2 infection resulting from miscellaneous levels of ACE2 expression within the host cell line and to enhance the efficiency of genetic perturbations, we generated a stable cell line termed A549-AC which constitutively expresses human ACE2 and Cas-9 (Supplementary Fig. 1a–c). The A549-AC cells were transduced with the lentiviral vectors encoding the gRNA library, followed by puromycin selection (Fig. 1a). After the puromycin-selected cells were passaged for 7 days, the cells were divided into two groups to be infected with the SARS-CoV-2 or to be treated with mock control (without infection) respectively (Fig. 1a). It is expected that cells with gRNAs targeting anti-viral host factors would be significantly depleted after SARS-CoV-2 infection compared with the control group, whereas those with gRNAs targeting pro-viral host factors would be enriched (Fig. 1a). To achieve 50-80% of cell loss (an optimal selection condition for dropout screens)^18^, we infected A549-AC cells with MOIs ranging from 0.1 to 40 and measured cell viability at 48-h post-infection. The results showed an MOI of 4.26 to yield 50% cell loss at 48-h post-infection (Supplementary Fig. 1d). Thus, an estimated MOI of 5 was selected in our screen. Indeed, at 48 h post-infection, around 50% of the cells exhibited CPE (Supplementary Fig. 1e). Based on genomic DNA amount isolated from the control and infected groups, our dropout screen reached a 47% of cell loss rate. Our screen exhibited sufficient gRNA reads for all sequenced samples, thus maintaining the library complexity (over 10 million reads from 30 million cells per sample; Supplementary Fig. 2a). The abundance of gRNAs among triplicate samples is highly correlated, demonstrating the consistency of our barcode sequencing results (Supplementary Fig. 2b). As expected for the working positive controls of effective CRISPR/Cas9 knockout, we observed a notable depletion in the abundance of the gRNAs targeting core essential genes after the 7-day expansion (Supplementary Fig. 2c).

To identify the enriched/depleted gRNAs and their target genes in the SARS-CoV-2 infection group compared with the control group, we used the Model-based Analysis of Genome-wide CRISPR/Cas9 Knockout (MAGeCK) as previously described^18^. Figure 1b summarizes the top ten enriched (pro-viral factors, colored in red) and depleted (anti-viral factors, colored in green) genes. Among them, three pro-viral hits (*ACE2, TMEM41B*, and *CTSL*) and one anti-viral hit (*LY6E*) were in common with previously published reports^11,12,14^. Using a fold change (FC) cut-off in gRNA abundance (|Log_2_FC|≥0.5) and a statistically significant threshold of *P* < 0.05, we found 63 enriched and 84 depleted genes and named them as pro-viral hits and anti-viral hits, respectively (Supplementary Data 1 and 2). Ingenuity Pathways Analysis (IPA) showed the pro-viral hits are not only from known signaling pathways required for viral replication and pathogenesis, but also from the pathways involved in phagosome maturation, endocytosis, and autophagy (left panel in Fig. 1c). In contrast, top anti-viral hits are from pathways involved in viral entry and exit, and cellular RNA polymerase II complex assembly (right panel in Fig. 1c). The roles of many enriched pathways in viral life cycle, such as iron homeostasis signaling, DNA repair, and protein ubiquitination, remain to be determined (Fig. 1c).

### Integrative analyses reveal the clinical relevance of identified host factors in COVID-19 patients

**Fig. 2:**
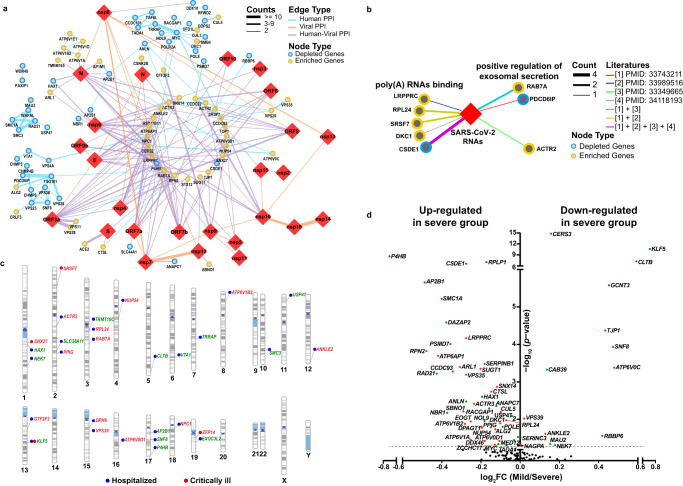
Integrative analysis revealing virus-host interactome networks and potential clinical relevance of identified host factors. **a** Protein-protein interactome (PPI) networks between viral proteins and host factors are identified by the dropout screen. 229 interactions between 26 SARS-CoV-2 proteins (red diamonds) and 147 human proteins (circles; depleted hits: blue; enriched hits: yellow) were found. The color of the edge indicates the type of interaction (blue: host-host PPI; orange: viral-viral PPI; purple: host-viral PPI) and the thickness of the edge indicates the count number of published datasets. **b** RNA-protein interactome networks between the viral RNA and host factors identified by the dropout screen. SARS-CoV-2 viral RNA was indicated as the red diamond; identified host factors were represented as circles (enriched hits: yellow; depleted hits: blue). The interaction between RNA and identified host factors was indicated as different edge types (colors: literature ID; thickness: count number of published manuscripts). **c** Gene variations in multiple identified host factors are associated with disease severity in COVID-19 patients. The Genome-Wide Association Study (GWAS) between variants of identified host factors and clinical features was performed by using the COVID-19-hgGWAS meta-analyses. “Hospitalized” indicates that single nucleotide polymorphisms (SNPs) of identified host factors were related to hospitalized COVID-19 patients, which were labeled with blue dots. “Critically ill” indicates SNPs of identified host factors were related to COVID-19 patients with severe respiratory symptoms, which were labeled with red dots. The names of enriched genes and depleted genes were labeled in red and green, respectively. **d** A volcano plot showing the changes in mRNA expression of identified host factors in epithelial cells from COVID-19 patients with and without severe illness. COVID-19 patients with mild symptoms or hospitalized in the ward were stratified in the mild group, whereas COVID-19 patients with severe symptoms or hospitalized in the intensive care unit (ICU) were stratified in the severe group. The fold change of gene expression was calculated. *P* values for each gene expression in different groups were calculated by Wilcoxon rank-sum’s post hoc test. Identified pro-viral factors and anti-viral factors which are differentially expressed (*P* < 0.05) in these two groups were highlighted with red and green dots, respectively.

To shed light on the potential mechanisms of host factors in SARS-CoV-2 infection, we first constructed a protein-protein interaction (PPI) network that contained 114,366 PPIs covering 13,716 human proteins and 30 SARS-CoV-2 proteins, based on the published PPI datasets^19^. We then constructed a sub-network that only covered 147 CRISPR screen hits (63 enriched and 84 depleted) and 26 SARS-CoV-2 proteins, including 79 PPIs between CRISPR screen hits, 22 PPIs between SARS-CoV-2 proteins, and 128 PPIs between SARS-CoV-2 and CRISPR screen hits (Fig. 2a). As shown in Fig. 2a, there are three major host PPI sub-networks. The first anti-viral sub-network is composed of the subunits of the cohesin complex, including *SMC1A*, *SMC3*, *RAD21*, and the cohesin complex release factor *WAPL*. Given that the cohesin complex plays critical roles in regulating mitosis and three-dimensional (3D) chromatin organization^20,21^, perturbing the cohesin complex could result in alteration of gRNAs distribution in our screen via regulating cell proliferation. To determine whether cohesin-related genes identified in our screens have additional roles in viral infection which are independent of their role in cell growth and survival, we stratified gRNAs targeting cohesin-related genes into two categories: Inscreen (gRNAs targeting the genes with statistically significant depletion in the SARS-CoV-2 group compared with the control group) and Others (gRNAs targeting the genes without statistically significant depletion in the SARS-CoV-2 group), and evaluated gRNA abundance in the cells from three groups: the Ref group (the puromycin-selected cells before 7-day expansion), the control group (the cells without viral infection after 7-day expansion) and the SARS-CoV-2 infection group (the cells with viral infection after 7-day expansion). As expected, gRNAs targeting both Inscreen and Others categories of cohesin-related genes are significantly depleted in the SARS-CoV-2 and the control group compared with the Ref group (Supplementary Fig. 3). Only the gRNAs targeting the cohesin-Inscreen category exhibit a further decrease in the SARS-CoV-2 group compared with the control group (Supplementary Fig. 3). These findings suggest that cohesin-related genes identified from our screen have anti-viral roles independent on regulating cell mitosis.

The second PPI sub-network is from the components of the ESCRT (The endosomal sorting complexes required for transport) pathway, including *CHMP3, CHMP4B*, *CHMP6, TSG101*, *VPS25, VPS28*, *VPS36*, *SNF8*, *VTA1*, *VPS4A*, and *PDCD6IP*. The ESCRT pathway has been shown to play an important role in regulating the infection of enveloped viruses, such as human immunodeficiency virus (HIV)^22^. The discovery of ESCRT-related PPI sub-network as anti-viral factors suggests that the ESCRT pathway may also play an important function in mediating SARS-CoV-2 infection. The third PPI sub-network is related to c-MYC and its interacting proteins, such as TRRAP. TRRAP was originally known as a co-activator for c-MYC and is critical for its oncogenic activities^23^, suggesting involvement of c-MYC-mediated transcriptional regulation in SARS-CoV-2 infection. Additionally, the host PPI sub-network analysis identified subunits of vacuolar-type ATPase (V-ATPase; including *ATP6V1E1*, *ATP6V1D*, *ATP6V1B2*, and *ATP6V1A*) as pro-viral factors (Fig. 2a).

We also constructed an RNA-protein interaction (RPI) network between SARS-CoV-2 RNAs and host proteins. This network includes 452 nodes (The SARS-CoV-2 RNAs are represented by one node) and 706 edges. The CRISPR screen hits that formed an interaction with SARS-CoV-2 RNAs fall into two major categories (Fig. 2b). The first category is the poly(A)-binding proteins, including four pro-viral factors (*LRPPRC*, *RPL24*, *SRSF7*, and *DKC1*) and one anti-viral factor (*CSDE1*). *RPL24* was recently reported to be involved in RNA metabolism, such as translation and splicing^24^. *LRPPRC* encodes an RNA-binding protein (RBP) that binds to poly(A)-mRNAs in the nucleus and mitochondria; LRPPRC is required for HIV-1^25^ and hepatitis C virus (HCV) infections^26^. *CSDE1* is an RBP that participates in the regulation of translation and mRNA turnover and is required for coxsackievirus B3 (CVB3) infection^27^. The second category includes the proteins that are positive regulators of exosomal secretion, including two pro-viral factors (*RAB7A*, *ACTR2*) and one anti-viral factor (*PDCD6IP*; Fig. 2b).

To uncover the clinical relevance of identified host factors, we integrated our CRISPR screen data with the COVID-19 GWAS meta-analysis results. The analysis showed that gene variations in 29 identified host factors (15 for pro-viral and 14 for anti-viral factors) were significantly associated with the critically ill and/or hospitalized COVID-19 patients (*P* < 0.001; Fig. 2c). In accordance with the PPI analyses, we found that the V-ATPase subunit *ATP6V1B2*, the c-MYC co-activator *TRRAP*, and the cohesin complex subunit *SMC3* were associated with the COVID-19 patient hospitalization, suggesting their clinical relevance to disease. We also found that *KLF5*, a transcription factor of the Krüppel-like factor subfamily of zinc finger proteins that showed anti-viral activity in our CRISPR screen, was associated with COVID-19 hospitalization and severe symptoms (Fig. 2c).

Lastly, we extracted transcriptional expression results of identified hits in epithelial cells from a published scRNA-Seq dataset of airway cells in COVID-19 patients^28^ and compared differences between mild and severe COVID-19 patients. Our results showed that 59 of 147 identified hits (30 pro-viral factors and 29 anti-viral factors) displayed differential expression between these two groups (*P* < 0.05; Fig. 2d). Based on the principle that pro-viral factors and anti-viral factors are expected to be upregulated and downregulated in severe COVID-19 patients, respectively, the concordance of changes of differentially expressed genes (DEG) between CRISPR screen and scRNA-Seq data was examined. We observed 24 pro-viral factors and 8 anti-viral factors showed a concordant up- or downregulation in the severe COVID-19 patients compared with the mild ones (Fig. 2d), suggesting these pro- and anti-viral factors may contribute to the severity of viral pathogenesis. Interestingly, *KLF5* (anti-viral factor) also displays a concordant change when comparing gene expression between severe and mild COVID-19 patients (Fig. 2d). Together with the GWAS analysis, these results highlight the clinical relevance of *KLF5* as an anti-viral factor.

### Selection of hits from the CRISPR dropout screen for individual validation

**Fig. 3:**
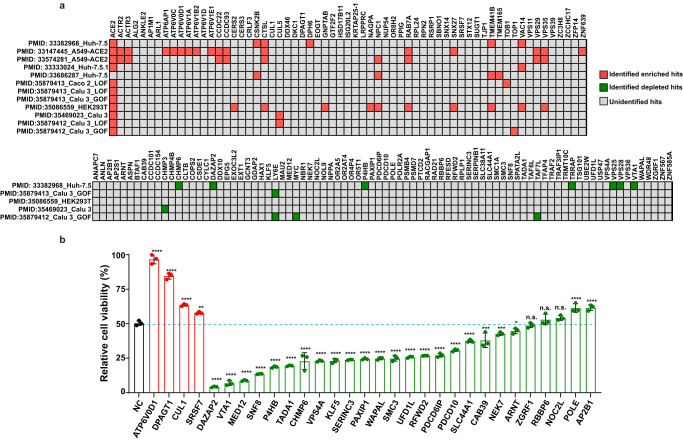
Validation of host factors identified from the CRISPR dropout screen. **a** Performance of identified host factors in previously reported SARS-CoV-2 screens. For each dataset, enriched and depleted hits that meet the listed criteria were marked as red and green squares, respectively. Others that fail to be identified in the listed datasets were marked as grey squares. **b** Effects of perturbation of top 30 hits on the CPE caused by SARS-CoV-2 infection. Four pro-viral and 26 anti-viral factors were selected for validation. A set of A549-AC cell lines expressing gene-specific gRNAs were infected with recombinant SARS-CoV-2 at MOI = 2.5. The percentages of viable cells were measured at 48 h post-infection. Data were normalized using the viability of corresponding cells at mock conditions. Statistical significance between cells expressing gene-specific gRNAs and non-targeting gRNA (NC) was determined by one-way ANOVA with repeated measurements. At least two independent experiments were performed, and samples were triplicated in each independent experiment. Data were analyzed using one-way ANOVA followed by Dunnett’s post hoc test and were presented as mean values  ±  SD; *n*  =  3 biologically independent samples. Exact *P* values were provided in the source data file. *P < 0.05; **P < 0.01; ***P < 0.001; ****P < 0.0001. n.s. not significant.

We compared the hits from our screen with those from previous SARS-CoV-2 screens. Supplementary Data 3 summarizes the 12 datasets from genome-wide CRISPR screens based on the CPE of SARS-CoV-2 on human epithelial cells. Although the majority of these screens were enrichment screens and focused on the discovery of pro-viral host factors, 5 of them included evaluation of depleted hits (anti-viral factors). Based on publicly available results, we set different criteria to determine whether our selected candidates are marked as putative host factors in corresponding screens (Supplementary Data 3). Among the hits selected by our screen, 29 enriched (46%) and 11 depleted (13%) hits can be identified by at least one of the listed datasets (Fig. 3a). Well-recognized pro-viral host factors such as *ACE2*, *CTSL*, and *TMEM41B* were identified by our screens and at least three additional CRISPR screens, validating the power of this genetic approach in exploring pro-viral factors in SARS-CoV-2 infection. However, significant amounts of the hits, particularly depleted hits, from our CRISPR dropout screen were not identified in previous studies. These results underscore the importance of the CRISPR dropout screens in filling in the knowledge gap of anti-viral host factors in SARS-CoV-2 infection.

Next, we selected 30 top-ranking candidates, including 4 pro-viral factors and 26 anti-viral factors, for individual validation. For each of the 30 selected genes, we prepared gRNA-expressing knockout (KO) A549-AC lines. Two independent sets of infection experiments were performed at the MOI of 2.5 and 5. The A549-AC line expressing non-targeting gRNAs served as a control line in all validation experiments. The results showed that 66.7% of the selected screen hits were validated in both sets of independent experiments (Supplementary Data 4). As shown in one representative experiment at MOI of 2.5 (Fig. 3b), inhibition of all tested 4 pro-viral factors significantly reduced the CPE after SARS-CoV-2 infection, with the most reduction observed in the ATP6V0D1- and DPAGT1-KO lines. On the other hand, knocking out 21 of 26 tested anti-viral factors increased cell death of infected cells, with the most increase observed in the DAZAP2- and VTA1-KO lines (Fig. 3b). Similar perturbation effects were observed at the condition with MOI of 5 (Supplementary Fig. 4a). Although multiple tested hits are also implied to directly control the growth of A549 cells (Supplementary Fig. 4b), the impact of each perturbation on cell growth does not correlate with their effect on susceptibility to CPE of infected cells. Furthermore, we seeded an equal number of cells before the viral infection and used cell numbers at the condition without viral infection to normalize the CPE in our validation experiments. Thus, these results implied that hits identified from our dropout screen are not biased by their roles in regulating cell growth. Taken together, literature cross-checks and individual validation demonstrate that our dropout screen successfully revealed a set of host factors/pathways with essential roles in SARS-CoV-2 infection.

### Mechanistic insights of top-ranking host factors in determining viral entry, attachment, and replication

Based on the phenotype of each perturbation in individual validation experiments, we focused on two pro-viral factors (*ATP6V0D1* and *DPAGT1*) and two anti-viral factors *(DAZAP2* and *VTA1)* whose knockout resulted in the most dramatic changes in CPE of infected cells for further mechanistic studies. *KLF5*, one of the top 10 anti-viral factors, was also selected because of its clinical relevance as indicated by both GWAS analysis (Fig. 2c) and transcriptome analysis of COVID-19 patients (Fig. 2d and Supplementary Fig. 5). This trend was not observed when we compared *KLF5* expression between non-COVID-19 pneumonia patients with variable severity (Supplementary Fig. 5), suggesting a specific role of *KLF5* as an anti-viral factor in modulating disease severity of COVID-19 patients. The expressions of pro-viral *ATP6V0D1* and *DPAGT1* and anti-viral *DAZAP2* were upregulated, whereas no difference in the expression was observed for pro-viral *VTA1*, in severe COVID-19 patients (Supplementary Fig. 5).

**Fig. 4:**
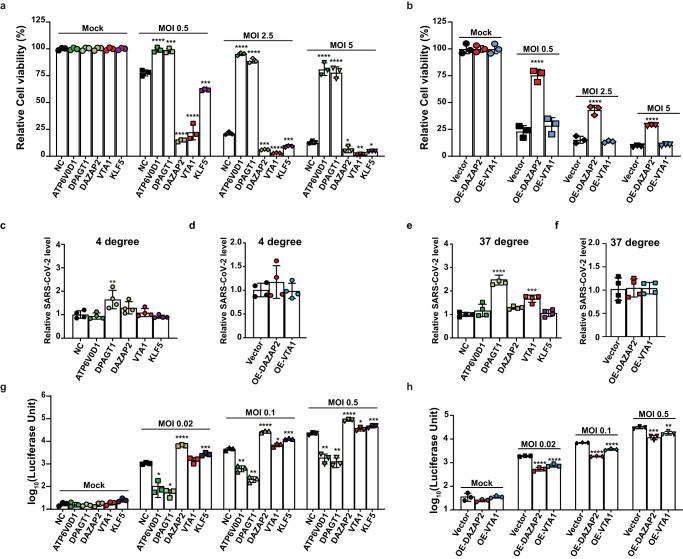
Impacts of top-ranking host factors on virion entry and replication pathways. **a**, **b** Effects of perturbation of top-ranking host factors on CPE caused by SARS-CoV-2 infection at different infection conditions. For gene-specific knockout (KO) effect (**a**), two pro-viral factors (*ATP6V0D1, DPAGT1)* and three anti-viral factors (*DAZAP2, VTA1*, *KLF5)* were selected. For gene-specific overexpression (OE) effect (**b**), two anti-viral factors (*DAZAP2, VTA1)* were selected. Genetically modified A549-AC cells were infected with recombinant SARS-CoV-2 at MOI = 0.5, 2.5, and 5 for 48 h. A549-AC cells expressing a non-targeting gRNA (NC) or the GFP vector served as control cells for the KO and OE experiments, respectively. Data were normalized using the viability of corresponding cells at mock conditions. At least two independent experiments were performed. Data were analyzed using one-way ANOVA followed by Dunnett’s post hoc test and were presented as mean values  ±  SD; *n*  =  3 biologically independent samples. **c**–**f** Effects of perturbation of top-ranking host factors on SARS-CoV-2 attachment and entry. Genetically modified A549-AC cells were infected with recombinant SARS-CoV-2 at MOI = 1. To evaluate the changes in the viral attachment (**c**, **d**), the infection was performed at 4 °C for 1 h; whereas to evaluate the changes in viral entry (**e**, **f**), the infection was performed at 37 °C for 1 h. The levels of RNAs encoding viral N protein and *ACTB* mRNAs were determined by RT-PCR. Viral RNA levels were normalized using the expression of *ACTB* mRNA. At least two independent experiments were performed. Data were analyzed using one-way ANOVA followed by Dunnett’s post hoc test and were presented as mean values  ±  SD; *n*  = 4 biologically independent samples. **g**, **h** Effects of perturbation of top-ranking host factors on SARS-CoV-2 replication. A549-AC cells with gene-specific KO (**g**) or OE (**h**) were infected with SARS-CoV-2-Nluc at MOI = 0.02, 0.1, and 0.5. The luciferase signals were measured 24 h post-infection. Statistical significances between KO/OE cells and control cells at each infection condition were determined by one-way ANOVA with repeated measurements. At least two independent experiments were performed, and samples were triplicated in each independent experiment. At least two independent experiments were performed. Data were analyzed using one-way ANOVA followed by Dunnett’s post hoc test and were presented as mean values  ±  SD; *n*  =  3 biologically independent samples. Exact *P* values were provided in the source data file. *P < 0.05; **P < 0.01; ***P < 0.001; ****P < 0.0001.

We explored the function of the five selected hits in SARS-CoV-2 infection. We first confirmed the knockout efficiency of each gene in the A549-AC cell lines by real-time PCR and Western blot (Supplementary Fig. 6a, b). Infection of these gRNA-expressing A549-AC lines with SARS-CoV-2 at various MOI conditions produced consistent phenotypes: (i) knocking out pro-viral *ATP6V0D1* or *DPAGT1* increased cell viability and (ii) knocking out anti-viral *DAZAP2*, *VTA1*, or *KLF5* decreased cell viability (Fig. 4a). Next, we attempted to produce overexpressing (OE) cell lines for individual genes. Among the five selected genes, we successfully generated stable A549-AC lines exogenously expressing *DAZAP2* or *VTA1* (Supplementary Fig. 6c, d). Interestingly, only overexpression of *DAZAP2* increased the cell viability of SARS-CoV-2-infected cells, whereas overexpression of *VTA1* did not affect the cell viability of infected cells (Fig. 4b). Moreover, we perturbed expression of the top 5 candidates in two additional human cell lines: H2023-AC (a lung adenocarcinoma cell line with ectopically expressed ACE2 and Cas9; Supplementary Fig. 7a) and Calu-3-Cas9 cells (a lung adenocarcinoma line with endogenously expressed ACE2 and ectopically expressed Cas9; Supplementary Fig. 7b). Among the top 5 candidates, *ATP6V0D1* and *DAZAP2* were successfully validated in both H2023-AC and Calu-3-Cas9 cells. *DPAGT1* was validated in H2023-AC cells but not in Calu-3-Cas9 cells, while *VTA1* was validated in Calu-3-Cas9 cells but not in H2023-AC cells (Supplementary Fig. 8). However, the effect of *KLF5* is unique to A549-AC, suggesting that our screen identified both shared and unique host factors among different cell types (Supplementary Fig. 8). Furthermore, in A549-AC cells, we observed similar effects of gRNAs targeting the top five candidates on CPE due to the infection by the SARS-CoV-2 Delta variant, as the SARS-CoV-2 WA1 strain (Supplementary Fig. 9), suggesting conserved roles of these genes in both SARS-CoV-2 strains.

We examined the steps of SARS-CoV-2 infection that are modulated by the identified host factors. To access virus attachment to cells with altered expression of selected factors, we incubated the KO or OE cells with SARS-CoV-2 at 4 °C for 1 h (which allowed the virus to attach to cells without entry) and measured the virions attached to the cell surface by viral RNA-based real-time PCR. DPAGT1-KO cells showed moderately enhanced viral attachment, but not in other types of KO or OE cells (Fig. 4c, d). We also co-cultured these cells with viral particles at 37 °C for 1 h (which allows virus attachment and entry into cells) to determine the impact of each perturbation on virus entry. Consistently, DPAGT1-KO and, to a less extent, VTA1-KO increased virus entry. However, no significant changes were observed in other cell lines when compared with control cells (Fig. 4e, f). Finally, we compared viral replication of SARS-CoV-2 expressing Nanoluc luciferase (SARS-CoV-2-Nluc) on different KO and OE cell lines. We previously showed that luciferase activity can be reliably used to quantify viral replication^29^. Despite variable magnitude, we observed a significant decrease in viral replication in ATP6V0D1-KO and DPAGT1-KO cells, and an increase in viral replication in DAZAP2-KO, VTA1-KO, and KLF5-KO cells. Conversely, DAZAP2-OE and VTA1-OE cells showed decreased viral replication, further confirming their anti-viral effect. Altogether, our results indicate that DPAGT1 facilitates SARS-CoV-2 attachment and entry, whereas ATP6V0D1, DAZAP2, VTA1, and KLF5 affect post-entry stages of SARS-CoV-2 replication.

**Fig. 5:**
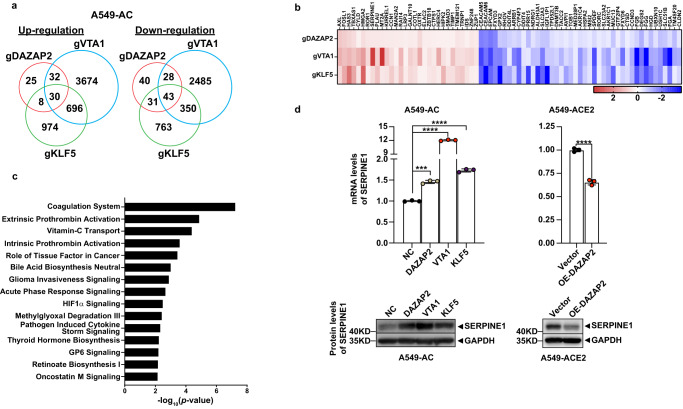
Molecular impacts of perturbing top-ranking anti-viral host factors. The transcriptomic profiles of DAZAP2/VTA1/KLF5-KO A549-AC cells and corresponding control cells were characterized by RNA-Seq. **a** A Venn diagram illustrating the degree of overlapped upregulated DEGs (left panel) and downregulated DEGs (right panel) identified from three types of KO cell lines. The numbers of DEGs that were significantly upregulated or downregulated in each type of KO cell lines ( | Log_2_FC | >0.25 and FDR < 0.25; compared with control cells) were indicated. **b** Heatmaps demonstrating mRNA expression changes of 73 shared DEGs among three types of KO cell lines. **c** Ingenuity Pathway Analysis of results from the shared DEGs among three types of KO cell lines. The top 15 canonical pathways displaying statistical significance were listed. *P* values for each gene set were calculated by using a Right-Tailed Fisher’s Exact Test. **d** Upregulation of SERPINE1 in DAZAP2/VTA1/KLF5-KO A549-AC cells and downregulation of SERPINE1 in DAZAP2-OE A549-ACE2 cells. mRNA levels of *SERPINE1* (upper panel) and protein levels of SERPINE1 (lower panel) were detected by real-time PCR and western blot, respectively. At least two independent experiments were performed. Data were analyzed using one-way ANOVA followed by Dunnett’s post hoc test or unpaired *T* test with two tails and were presented as mean values  ±  SD; *n*  =  3 biologically independent samples. Exact *P* values were provided in the source data file. ***P < 0.001; ****P < 0.0001.

Results from our group and others have strongly suggested that *ATP6V0D1* and *DPAGT1*, two identified pro-viral factors, may promote SARS-CoV2 infections via regulating the function of lysosome/endosome and altering glycosylation status of viral receptors such as ACE2, respectively^30–33^. Therefore, we focused on exploring mechanisms/pathways utilized by three validated anti-viral factors. By comparing the transcriptomic profiles of gRNA-expressing cells (Supplementary Fig. 10a), DEGs in DAZAP2/VTA1/KLF5-KO A549-AC cells were identified based on the statistical significance and fold changes of each comparison between KO and control cells. As shown in Fig. 5a, there are shared and unique DEGs among DAZAP2/VTA1/KLF5-KO A549-AC cells. The expression changes of each shared DEG were shown in Fig. 5b. Pathway analysis revealed a set of pathways in which shared DEGs and unique DEGs are enriched (Fig. 5c and Supplementary Fig. 10b–d). The top enriched pathway of shared DEGs is related to the coagulation system (Fig. 5c). Notably, the following three enriched pathways (Extrinsic prothrombin activation, vitamin-C transport, intrinsic prothrombin activation) are also highly associated with the coagulation system^34–36^. The shared DEGs in the coagulation system include *FGA, FGB, FGG, PLAU*, and *SERPINE1*. Among them, SERPINE1 is a typical member of the Serpin family proteins, which functions as a ‘suicide inhibitor’ binding both tissue type and urokinase plasminogen activator (tPA and uPA)^37^. Increased *SERPINE1* expression has been reported in patients with severe COVID-19 symptoms when compared to patients with moderate symptoms^38^. Additionally, the level of *SERPINE1* was positively associated with mortality of COVID-19 and spontaneous ex vivo clot lysis^39^. scRNA-Seq results of airway epithelial cells in COVID-19 patients also show a trend of a positive association between *SERPINE1* expression and poor outcomes in COVID-19 patients (Supplementary Fig. 11). Consistent with our RNA-Seq analysis, inhibition of *DAZAP2*, *VTA1*, or *KLF5* expression in A549-AC consistently elevated SERPINE1 expression at both mRNA and protein levels (Fig. 5d). Conversely, DAZAP2-OE cells, which showed reduced sensitivity to SARS-CoV-2 infection, display significantly reduced expression of SERPINE1 (Fig. 5d). Our findings suggest these three anti-viral host factors may share a common mechanism of regulating host cell response to SARS-CoV-2 infection, through controlling *SERPINE1* expression. Together with the gene-specific regulatory mechanisms (Supplementary Fig. 10b–d), anti-viral factors may exhibit anti-viral effects and/or impact cytopathic effects upon SARS-CoV-2 infection to varied extent or magnitude.

## Discussion

As summarized in the Supplementary Fig. 12, we performed a genome-wide dropout screen using 48-h SARS-CoV-2 infection based on the impact of genetic perturbation on CPE and integrated our screen results with GWAS analysis, viral RNA and protein interactomes, and transcriptional profiles of epithelial cells in COVID-19 patients. Many host factors/pathways, such as those involved in airway epithelium damage and the regulation of SARS-CoV-2 viral load, have been reported to contribute to the severity of COVID-19^40,41^. By integrating results from CPE-based screens and correlative analysis of clinical datasets, we sought to better understand host factors that potentially impact the COVID-19 pathogenesis.

Despite our experimental condition being optimized to identify anti-viral host factors of SARS-CoV-2, several well-recognized pro-viral host factors/pathways, including *ACE2*, *CTSL*, and the autophagy pathway, were re-validated in our screens. Additionally, 25 of 30 hits identified from our screens were confirmed by individual gene validation. These results indicate the reproducibility of our screen and provide confidence in the list of putative host factors generated in this study. Importantly, when compared with the results from published genomic SARS-CoV-2 screens (either enrichment screens or bi-direction screens), a significant portion of the host factors in our list (87% of anti-viral factors and 54% of pro-viral factors) were not identified by previous screen conditions, suggesting the importance of the dropout screens to identify novel anti-viral factors.

By leveraging viral RNA and protein interactomes, we found that six anti-viral factors identified in this study belong to the cohesin complex, a central organizer of the 3D genome structure^42^. Consistent with our finding, a recent study showed that SARS-CoV-2 was able to rewire host chromatin organization in human cells to confer immunological gene deregulation^43^. Our finding of the subunits of the cohesin complex as inhibitors of SARS-CoV-2 infection after controlling for their effect on mitosis, suggests an important role of 3D chromatin organization in mediating host-virus interactions, which could be independent of their effects on regulating mitosis pathway.

We selected five host factors for mechanistic studies. *ATP6V0D1* encodes a transmembrane V0 domain D subunit of Vacuolar-type H + -ATPase (V-ATPase), which is the primary proton pump for H+ homeostasis and mediates acidification of eukaryotic intracellular organelles. Besides ATP6V0D1, we found a group of V-ATPases interact with the M protein of SARS-CoV-2. These proteins are highly conserved enzymes and are often localized to the plasma membranes of many cell types^44^. Plasma membrane V-ATPases play crucial roles in pH homeostasis and coupled transport^45,46^. Perturbing *ATP6V0D1* in host cells has limited impacts on viral attachment and entry of SARS-CoV-2. However, inhibiting *ATP6V0D1* reduces viral replication and decreases CPE. *DPAGT1* is another pro-viral host factor selected for further studies. It encodes an enzyme that catalyzes the initial step of protein N-glycosylation^47^. N-glycosylation of asparagine is a common post-translational modification, which has been reported to regulate protein stability and function^48^. In our study, *DPAGT1* KO promotes viral entry but reduces viral replication in A549-AC lines. Additionally, *DPAGT1* KO significantly inhibits the CPE induced by SARS-CoV-2 in two tested lines (A549-AC and H2023-AC) whose ACE2 are ectopically expressed. These results suggest that the role of DPAGT1 in SARS-CoV-2 virus life cycle revealed in these two lines is independent on the regulation of ACE2 mRNA expression. Furthermore, higher expression of these two pro-viral factors was found in severe COVID-19 patients. These findings highlight the roles of V-ATPases and N-glycosylation in the pathogenesis of COVID-19. However, further investigations are needed to confirm whether *DPAGT1* KO can alter the binding between spike proteins and functional receptors for viral entry via regulations of protein glycosylation. It is also possible that additional mechanism occurring independent of glycosylation contributes to pro-viral effect of *DPAGT1*.

Furthermore, we characterized changes in gene expression profiles in cell lines with knockout of individual validated anti-viral host factors, DAZAP2, VTA1, and KLF5. Our analyses revealed a panel of molecular pathways that could be under the control of all or individual factors. The expression of genes in the coagulation system including *FGA, FGB, FGG, PLAU*, and *SERPINE1* exhibit consistent upregulation in all three KO cells. We further confirmed a negative regulation of SERPINE1 expression by *DAZAP2, VTA1*, and *KLF5* at both mRNA and protein levels. Together with the fact that upregulated SERPINE1 was found in COVID-19 patients with poor clinical outcomes^38,39^, negative correlations between SERPINE1 and three validated anti-viral host factors provide an additional rationale to target SERPINE1 to prevent severe COVID-19.

Besides the potential shared mechanisms of anti-viral host factors, our data suggested that these factors might have unique mechanisms in regulating the pathogenesis of COVID-19. DAZAP2 was identified as an anti-viral factor that contains several Src homology (SH) domains. As an adaptor protein, DAZAP2 plays important roles in regulating several important biological and pathological processes such as DNA damage^49^, inflammation^50^, and activation of Wnt signaling^51^. Although there is no interaction between DAZAP2 and SARS-CoV-2 proteins or RNAs in our interactomes analysis, DAZAP2 was identified as a flavivirus RNA-binding host factor by using yeast three-hybrid systems^52^. Knockout and overexpression of *DAZAP2* significantly increased and decreased viral replication, respectively; however, neither perturbation affected virus entry. These results provide a strong rationale to determine whether *DAZAP2* interacts with viral RNAs/proteins and, if so, how these interactions modulate viral replication. Furthermore, DEGs identified from DAZAP2-KO cells are enriched in the “Osteoarthritis pathway” and the pathway named as “Role of osteoblasts in rheumatoid arthritis signaling”. This finding may provide the opportunity to explore new mechanisms of joint aging and osteoarthritis after acute and post-acute COVID-19^53^. We also noticed that knocking out *DAZAP2* significantly alters the expression of genes involved in the metabolic pathways of sphingosine, ceramide, and sphingolipids. These metabolites have been reported to serve as Damage-Associated Molecular Patterns (DMAPs) for immune activation and as modulators of SARS-CoV-2 infection^54^.

VTA1 is a component from ESCRT, which sorts membrane proteins for degradation in the lysosomes. The ESCRT-related PPI sub-network was found in the interactome analysis of our anti-viral hits. Increased viral entry was observed in *VTA1*-KO cells, suggesting its potential role in viral internalization. Besides its important function in regulating vascular transport as previously reported^55^, our RNA-Seq data suggested that VTA1 was involved in the biological processes of cell cycle regulation, epithelial adherents junction signaling, and DNA damage response, which might be recognized to play crucial roles in regulating cell death, epithelium integrity triggered by stress and virus infection conditions.

Last, we observed strong correlations between COVID-19 severity and *KLF5* expression. The variants in *KLF5* genomic region and reduced *KLF5* expression are associated with severe COVID-19 patients. KLF5 belongs to a highly conserved Krüppel-like zinc finger transcription factor family, which plays crucial roles in various physiological and pathological processes, including cell cycle, angiogenesis, migration, apoptosis, autophagy inflammation, self-renew, and differentiation^56^. KLF5 was recently recognized as a transcriptional orchestrator of epidermal cell differentiation and pathophysiology of ischemic heart failure by governing sphingolipid metabolism^57,58^. As glycosphingolipid is essential for viral replication, its expression level is significantly induced upon SARS-CoV-2 infection^59^. These results implied that KLF5 could regulate SARS-CoV-2 replication by modulating sphingolipid metabolism. This notion is further supported by the clinical observation that reduced sphingosine levels are associated with the development of symptomatic COVID-19 patients compared to asymptomatic counterparts^60^. Our data also suggest that KLF5 plays a role in regulating sphingolipid metabolism, collagen metabolism, matrix metalloproteases, and wound healing factors. Dysfunctions in those components were reported to contribute to abnormal extracellular matrix (ECM) and metabolic environment during SARS-CoV-2 infection^54,61^.

Like all other in vitro screens, our screen using A549 cells has limitations to obtain a completed list of host factors in SARS-CoV-2 infection. As the pathogenesis, disease severity, or recovery of COVID-19 are also controlled by host factors in other types of cell types, such as immune cells, in vitro screens may not be able to discover host factors in these cell types. Second, malignant and/or immortalized cell lines were used in most of the reported genome-wide screens. Last, although our results from genome-wide genetic screens and a broad ranges of correlative analyses provide a rich resource to facilitate SARS-CoV-2 related research, mechanistic validations are needed to functionally confirm the pathways revealed by our correlative analyses. Despite these limitations, our study has provided a prioritized list of host factors that warrant further investigation of their role in pathogenesis, disease severity, or recovery of COVID-19 in more complex in vivo systems. Recently, inhibition of Cathepsin L (also repeatedly identified in our screens) by a chemically engineered nano system encapsulating CRISPR-Cas13d was reported to alleviate SARS-CoV-2-induced pathological changes in vivo^62^, highlighting the potential of targeting hits identified from genetic screens to develop safe CRISPR-based countermeasures with an anti-viral spectrum.

In summary, our genetic dropout screen and integrative database analysis reveal a broad range of molecules and pathways including the cohesin complex, V-ATPases, N-glycosylation, and sphingolipid metabolism, all of which modulate viral replication and/or COVID-19 manifestation. As the function of these host factors is largely unknown, our findings have broadened the understanding of host-viral interactions, particularly about anti-viral host factors, during SARS-CoV-2 infection and the pathogenesis of COVID-19.

## Methods

### Cell lines and recombinant SARS-CoV-2

A549, H2023, Calu-3, and HEK293T cell lines were obtained from the American Type Culture Collection (ATCC, Bethesda, MD). All cell lines and their genetically modified cell lines were cultured in Dulbecco’s modified Eagle’s medium (DMEM) supplemented with 10% heated-inactivated fetal bovine serum (FBS; #S11150, R&D System, Minneapolis, MN) and 100 μg/ml of Normocin (ant-nr-1, InvivoGen, San Diego, CA). The A549-hACE2 cells that stably express human *ACE2* were generated previously^29^ and grown in the culture medium supplemented with 10 μg/mL Blasticidin (#A1113903; ThermoFisher, Waltham, MA). Cells were grown at 37 °C with 5% CO_2_. All cell lines were authenticated by short tandem repeat fingerprinting or the expression of tagged markers used for genetic modification. The mycoplasma detection kit (#13100-01, SouthernBiotech, Birmingham, AL) was used to routinely monitor for mycoplasma contamination of cultured cells. The maximum length of time of in vitro cell culture between thawing and use in the described experiments was 2 weeks.

The recombinant SARS-CoV-2 WA1 strain (WA1 strain was used in all experiments except when otherwise mentioned) and the Delta variant were generated by a previously described reverse genetic system based on the strain 2019-nCoV/USA_WA1/2020 derived from the first patient diagnosed in the US^63,64^. The recombinant SARS-CoV-2 was used to screen host factors regulating CPE. The nanoluc luciferase severe respiratory syndrome coronavirus 2 (SARS-CoV-2-Nluc) established in our previous study^29^ was used to evaluate the involvement of identified host factors in viral entry and replication. Experiments with SARS-CoV-2 and nano-Luciferase virion were performed in a BSL-3 laboratory by personnel equipped with powered air-purifying respirators. All procedures were followed by biosafety protocols approved by the Institutional Biosafety Committees at the University of Houston and the University of Texas Medical Branch at Galveston.

### Establishment of genetically modified cell lines

Lentivirus-based gene delivery was used to either genetically knockout gene-of-interests (GOIs) or ectopically express GOIs. To generate lentiviral supernatants, HEK293T cells were seeded 16 h before transfection and transfected with the lentiviral vectors encoding different gRNAs or GOIs, along with lentiviral packaging plasmids, pCMV-VSV-G and psPAX2 (#8454 and #12260, Addgene) by the jetPRIME transfection reagent (#101000046, VWR, Radnor, PA) according to the manufacturer’s protocol. Viral supernatants were collected 72 h post-transfection and filtered by 0.45 μm PVDF Syringe Filter Unit (#SLHV033NK, Millipore-Sigma, Burlington, MA) to remove cell debris. Designated titers of lentivirus were used to infect cells in the presence of 8 μg/ml hexadimethrine bromide (#107689, Sigma-Aldrich, St Louis, MO).

To generate A549, H2023, and Calu-3 cells expressing Cas-9 (tagged by both flag and GFP) for gene editing, A549-hACE2, H2023-hACE2, and Calu-3 cell lines were transduced lentivirus expressing lentiCas9-EGFP (#63592, Addgene). The Cas-9 expressing A549-hACE2 (A549-AC), H2023-hACE2 (H2023-AC), and Calu-3-Cas9 cell lines were generated by sorting GFP^+^ cells 72 h after lentivirus transduction. To genetically suppress the expression of GOIs in cells, lentiviral gRNA-expressing vectors were constructed. Fully synthesized double strand (ds) DNA fragments (Twist Bioscience, San Francisco, CA) encoding gene-specific gRNAs were inserted into the lentiGuide-Puro (#52963, Addgene) as previously described^18^. The forward sequences of protospacer sequences of gRNAs were listed in Supplementary Data 5. A549-AC, H2023-AC, and Calu-3-Cas9 cells were transduced with gRNA-expressing vectors and followed by antibiotic selection using 1 μg/ml of puromycin (#A1113803, Gibco, Carlsbad, CA) to generate stable KO cell lines. Cells transduced with the viral vector encoding a non-targetable gRNA (NC) were generated and served as control cells. To ectopically express GOIs in cells, dsDNA fragments encoding FLAG-tagged human open reading frames (ORFs) of *DAZAP2* (NM_014764.4) and *VTA1* (NM_016485.5) were inserted into the lentiviral vector, pLVX-IRES-ZsGreen1 (#PT4064-5, Takara Bio, San Jose, CA). A549-hACE2 cells were transduced with lentiviral ORF vectors. Stable cell lines were generated by sorting GFP^+^ cells 72 h after lentivirus transduction. Cells transduced with pLVX-IRES-ZsGreen1 were generated and served as control cells.

### Detection of ACE2 and Cas9 expression levels by flow cytometry

To confirm the expression of ACE2 and GFP-tagged Cas9, A549-AC cells were harvested and stained with anti-human ACE2 antibody (FAB9332R, R&D systems, Minneapolis, MN; at the working concentration of 1:100). The expression levels of ACE2 and tagged GFP were determined by an LSRFortessa X-20 (BD Biosciences).

### CRISPR dropout screens

The optimized human genome-wide knockout (KO) CRISPR Library (H1) consisting of 92,817 gRNAs targeting 18,436 genes (5 gRNAs for each gene) was purchased from Addgene (Pooled Library #1000000132) and used to generate lentivirus as described above. 1.5 × 10^8^ of A549-AC cells were transduced with pooled library lentivirus at a low multiplicity of infection (MOI; ~0.15–0.2) to ensure that each cell would receive only one gRNA as previously described^18^. In all, 48 h after transduction, cells were cultured in the growth medium in the presence of 1μg/ml puromycin to select transduced cells. In all, 72 h after puromycin selection, 30 million cells were collected and used as the reference sample. Seven days post puromycin selection, pooled gRNA-expressing A549-AC cells were re-seeded in T175 cell culture flasks (10 million cells/flask). The next day, for the group receiving virus infection, SARS-CoV-2 was used to infect 2 × 10^8^ of pooled A549-AC cells at MOI = 5 for 48 h. Pooled A549-AC cells cultured in the same assay medium were collected and served as the control group. 48 h post SARS-CoV-2 infection, non-adhesive cells were removed by repeated wash using pre-warmed PBS. Adherent cells were harvested by using 0.25% Trypsin-EDTA (#15050065, ThermoFisher) and washed three times using PBS. Three replicated samples were collected for each group.

Genomic DNA from all cell samples was extracted by using TRIzol (#15596026; ThermoFisher) according to the manufacturer’s protocol. DNA fragments containing gRNA sequences were amplified and barcoded with adaptation by nested polymerase chain reaction (PCR) as previously described^18^. The quality and concentration of all PCR products were determined by the Qubit ssDNA high sensitivity assay kit (#Q10212; ThermoFisher) and the bioanalyzer High Sensitivity DNA Kit (#5067-4626 2100; Agilent, Santa Clara, CA). Samples were then sequenced by Illumina Next-Generation Sequencing (NSG) at the MD Anderson Cancer Center Advanced Technology Genomics Core.

### Assays to evaluate the virus-induced cytopathic effect

A set of genetically modified A549-AC cell lines (10,000 cells per well in DMEM medium containing 2% FBS) were plated into clear flat-bottom 96-well plates. On the next day, the recombinant SARS-CoV-2 was used to infect pre-seeded A549-AC cells at designated MOIs (0.5, 2.5, or 5). Forty-eight hours after viral infection, 4 μL of Cell Counting Kit-8 (#CCK-8, Sigma-Aldrich, St Louis, MO) was added to each well. After incubation at 37 °C for 90 min, absorbance at 450 nm was measured using a Cytation5 multi-mode microplate reader (BioTek, Winooski, VT). The relative cell viability of each group was calculated by normalizing the absorbance of the control groups (set as 100%). At least two independent experiments were performed to determine the sensitivity of genetically modified cells to virus-induced CPE. For each experiment, triplication was performed for all groups.

### Assays to evaluate viral entry and replication

Genetically modified A549-AC cell lines (10,000 cells per well in DMEM medium containing 2% FBS) were plated in white opaque 96-well plates. On the next day, the recombinant SARS-CoV-2-Nluc virus^29^ was used to infect pre-seeded A549-AC cells at designated MOIs (0.02, 0.1, and 0.5). In all, 18 h after infection, cells were applied to Nano-Glo® Dual-Luciferase® reporter assays (#N1610; Promega, Madison, WI) according to the manufacturer’s instructions. Luciferase signal from all samples was measured using a Synergy™ Neo2 microplate reader. At least two independent experiments were performed to determine the sensitivity of genetically modified cells to virus-induced cytopathic effect. For each experiment, triplication was performed for all groups.

### Assays to evaluate viral entry and attachment

Genetically modified A549-AC cell lines (25,000 cells per well in DMEM medium containing 2% FBS) were plated in 12-well plates. On the next day, SARS-CoV-2 was used to infect pre-seeded cells at MOI = 1.0. For the virus entry assay, cells were co-incubated with SARS-CoV-2 at 37 °C for 1 h. For the viral attachment, cells were co-incubated with the virus at 4 °C for 1 h. After co-incubation, RNAs were isolated from infected cells by Trizol and Direct-zol RNA Miniprep Plus Kits (#R2071; Zymo Research, Irvine, CA) according to the manufacturer’s instructions. The relative expression levels of the SARS-CoV-2 nucleocapsid (N) protein were determined from the quantitative Real-time PCR (qRT-PCR) using the iTaq Universal One-Step RT-qPCR Kit (#1725151; Bio-Rad, Hercules, CA). Triplication of PCR reactions was included in all assays. The expression levels of *ACTB* were used for data normalization. The sequences of RT-PCR primers are listed in Supplementary Data 6.

### Immunoblot analysis

To verify the expression of GOIs, proteins were extracted by lysed cells using RIPA Lysis and Extraction Buffer (#89900; ThermoFisher) and the concentrations of protein samples were quantified with the Pierce BCA Protein Assay Kit (#23225; ThermoFisher). The western blot analysis was used to determine the expression of protein-of-interest. The intensity of protein bands was detected by Immobilon Western Chemiluminescent HRP Substrate (#WBKLS0500; Millipore-Sigma, Burlington, MA) using the ChemiDoc Imaging System. Antibodies targeting β-actin (8H10D10, #3700) and SERPINE1 (E3I5H, #49536) were purchased from the Cell Signaling Technology (Danvers, MA). Anti-human ACE2 antibody (AF933) was purchased from R&D Systems. Antibodies targeting DAZAP2 (G-4, sc-515182) and GAPDH (0411, sc-47724) were purchased from Santa Cruz Biotechnology (Dallas, TX). Anti-KLF5 antibody (21017-1-AP) was purchased from Proteintech Group (Rosemont, IL) and the monoclonal anti-FLAG antibody (M2, #F3165) was purchased from Millipore-Sigma. The working concentration for primary antibodies listed above is 1:1000. HRP-conjugated secondary antibodies including anti-rabbit IgG (#7047) and anti-mouse IgG (#7076) were purchased from the Cell Signaling Technology (Danvers, MA). The working concentration for both secondary antibodies is 1:4000. Uncropped and unprocessed scans of the blots were found in the Source Data file.

### Bioinformatic analysis

The MAGeCK (v0.5.9.4) count module was used to calculate the read count of individual sgRNAs in different samples with the following parameters: “-l human_sgrna_sequences_A.library --control-sgrna human_sgrna_sequences_A.library.negctrl --norm-method control --sample-label C-1,C-2,C-3,control1,control2,control3,Ref-1 -n COVID-19_CRISPR_210212.count --fastq files.fq”. MAGeCK test module was then applied with parameters “-k COVID-19_CRISPR_210212.count.txt -c control1,control2,control3 -t C-1,C-2,C-3 --norm-method control --keep-tmp -n COVID-19_CRISPR_210212_C_control --control-sgrna human_sgrna_sequences_A.library.negctrl --gene-lfc-method secondbest”, to identify the genes that showed a significant differential selection ( | log_2_(fold-change)| ≥0.5 and *P* < 0.05) between the control and SARS-CoV-2 infection groups (Supplementary Software).

For the whole transcriptome analysis of DAZAP2-KO, VTA1-KO, and KLF5-KO and negative control gRNA-transduced A549-AC cell lines, RNAs were extracted from cells in triplicate using a Direct-zol RNA Miniprep Plus kit (R2071, Zymo Research, Irvine, CA). RNA-Seq libraries were prepared and subjected to paired-end sequencing by the Next-Generation Sequencing Core Facility at The University of Texas Medical Branch. The raw sequencing reads were quantified using kallisto^65^ and mapped to GRCh38 as transcripts per million for differential expression analysis.

QIAGEN Ingenuity Pathway Analysis (Version: 90348151) was used to select, annotate, and visualize genes by function and pathway. The DEGs with a cut-off of false discovery rate less than 0.25 and |Log_2_FC|>0.25 were selected for the IPA analysis. IPA calculates a *P* value for each gene set using a Right-Tailed Fisher’s Exact Test to reflect the likelihood of the gene set and the DEGs being random. IPA analysis identified those canonical pathways differentially expressed (*p* < 0.05) between comparison groups.

The COVID-19 GWAS meta-analyses results (release 6)^66^ for “Hospitalized covid vs. population” and “Very severe respiratory confirmed covid vs. population” were downloaded from the COVID-19 Host Genetics Initiative (https://www.covid19hg.org/). The CRISPR screen hits that are within +/− 10 kb of the SNPs reaching the significance level of *P* < 0.001 in the GWAS meta-analysis were then identified as candidate genes associated with “Hospitalized” and “Critically-ill” conditions (Supplementary Software).

We also performed an integrated analysis of host-host and host-viral interactome based on the protein-protein interaction (PPI) data from the BioGRID database (Release 4.4.205)^19^. Briefly, we first constructed a human-human and human-SARS-CoV-2 PPI (CoV-2_HsPPI) network that contain the human-human or human-viral PPIs supported by at least two independent experiments which resulted in a total of 114,366 interactions covering 13,716 human proteins and 30 SARS-CoV-2 proteins. By selecting the CRISPR screen hits and the SARS-CoV-2 proteins as the seed nodes, a sub-network was then constructed as the CRISPR screen hits related host-host and host-viral PPI network (Supplementary Software).

Besides the PPI, we integrated four host-viral protein-RNA interactome (RPI) datasets^67–70^ that characterize the interaction between human protein and SARS-CoV-2 RNAs to construct a “SARS-CoV-2 RNAs – human proteins” (CoV-2_HsRPI) network that included 452 nodes and 706 edges. By selecting the CRISPR screen hits as the seed nodes, a sub-network was then constructed as the CRISPR screen hits related RPI network.

scRNA-Seq analysis was performed using a previously published and de-identified dataset^28^. Results of airway epithelial cells were extracted from the dataset generated by Wauters et al., which include 65,166 cells from 35 pneumonia patients, 22 of whom tested positive for SARS-CoV-2, while the other 13 are infected by other pathogens. Among all the patients, 14 of whom experienced mild symptoms, while the other 21 are severe cases. The data analysis was performed as described in previous studies^28^. Briefly, based on the source of cells, epithelial cells were dissected into four groups, namely non COVID19_mild, non COVID19_severe, COVID19_mild, and COVID19_severe. To test the hypothesis that whether candidates identified in our screen are differentially expressed among epithelial cells derived from different groups, we use the Kruskal–Wallis test to identify if any group of cells is significantly different from another, and if so, the Wilcoxon rank-sum test was used as a post hoc test to identify the pairs of groups that are significantly different (Supplementary Software).

### Statistical analyses

Summary statistics (e.g., mean, SEM) of the data are reported. Assessments of differences in continuous measurements between the two groups were made using a two-sample *t* test. Multiple group comparisons were performed by Analysis of Variance (ANOVA) with repeated measures. A *P* value of less than 0.05 was considered significant. Graph generation statistical analyses were performed using the Prism software program (GraphPad Software 9.0.2), Tableau 8.2 software program (Tableau Software), and R software programming language (version 3.1.0). The sample size for each experiment was chosen based on the study’s feasibility given its exploratory nature.

### Reporting summary

Further information on research design is available in the Nature Portfolio Reporting Summary linked to this article.

### Supplementary information

Supplementary Information

Description of Additional Supplementary Files

Supplementary Data 1

Supplementary Data 2

Supplementary Data 3

Supplementary Data 4

Supplementary Data 5

Supplementary Data 6

Supplementary Software

Reporting Summary

### Source data

Source Data

## Data Availability

The data that support the findings of this study has been deposited in public data repositories, and/or is presented as Supplementary Information with the manuscript. Raw data files from the genome-wide CRISPR dropout screen are deposited in the GEO repository under accession code GSE209750. Raw RNA-Seq data have been deposited in the GEO repository under accession code GSE246452. Source data are provided with this paper.
