# Supplementary material for: Integrated multi-omics analyses identify anti-viral host factors and pathways controlling SARS-CoV-2 infection
Source: Nat Commun. 2024 Jan 2;15:109. doi: 10.1038/s41467-023-44175-1 (PMC10761986; doi:10.1038/s41467-023-44175-1)
Supplement: Supplementary file 1 — Supplementary Information [file 41467_2023_44175_MOESM1_ESM.pdf]

Supplementary information for

Integrated multi-omics analyses identify anti-viral host factors and pathways controlling SARS-CoV-2 infection

Jiakai Hou[1#], Yanjun Wei[2#], Jing Zou[3#], Roshni Jaffery[1], Long Sun[3], Shaoheng Liang[2,4,16], Ningbo Zheng[1], Ashley M. Guerrero[1], Nicholas A. Egan[1], Ritu Bohat[1], Si Chen[1], Caishang Zheng[2], Xiaobo Mao[5], S. Stephen Yi[6,7], Ken Chen[2], Daniel J. McGrail[8], Nidhi Sahni[2,9], Pei-Yong Shi[3,10,11,12,13,14*], Yiwen Chen[2,15*], Xuping Xie[3,14*], and Weiyi Peng[1*]

[1]Department of Biology and Biochemistry, University of Houston, Houston, TX.

[2]Department of Bioinformatics and Computational Biology, The University of Texas MD Anderson Cancer Center, Houston, TX.

[3]Department of Biochemistry & Molecular Biology, The University of Texas Medical Branch, Galveston, TX, USA.

[4]Department of Computer Science, Rice University, Houston, TX, USA.

[5]Neuroregeneration and Stem Cell Programs, Institute for Cell Engineering, Department of Neurology, Johns Hopkins University School of Medicine, Baltimore, MD, USA.

[6]Department of Oncology, Livestrong Cancer Institutes, and Department of Biomedical Engineering, The University of Texas at Austin, Austin, TX, USA

[7]Interdisciplinary Life Sciences Graduate Programs (ILSGP) and Oden Institute for Computational Engineering and Sciences (ICES), The University of Texas at Austin, Austin, TX, USA

[8]Center for Immunotherapy and Precision Immuno-Oncology, Cleveland Clinic, Cleveland, OH.

[9]Department of Epigenetics and Molecular Carcinogenesis, The University of Texas MD Anderson Cancer Center, Houston, TX, USA.

[10]Institute for Human Infections and Immunity, The University of Texas Medical Branch, Galveston, TX, USA.

[11]Sealy Institute for Vaccine Sciences, The University of Texas Medical Branch, Galveston, TX, USA.

[12]Sealy Center for Structural Biology & Molecular Biophysics, The University of Texas Medical Branch, Galveston, TX, USA.

[13]Institute for Translational Science, The University of Texas Medical Branch, Galveston, TX, USA.

[14]Sealy Institute for Drug Discovery, The University of Texas Medical Branch, Galveston, TX, USA.

[15]Quantitative Sciences Program, MD Anderson Cancer Center, UT Health Graduate School of Biomedical Sciences, Houston, TX, USA

[#]These authors contributed equally

[16]Current affiliation: Computational Biology Department, School of Computer Science, Carnegie Mellon University, Pittsburgh PA

***Corresponding Authors:** Weiyi Peng, University of Houston, 3455 Cullen Blvd., Suite 342 Houston, TX 77204-5001, Phone: 713-743-6941, E-mail: wpeng2@Central.uh.edu; Xuping Xie, The University of Texas Medical Branch, 301 University Blvd., Galveston TX 77550, Phone: 409-772-1725, E-mail: xuxie@UTMB.edu; Yiwen Chen, The University of Texas MD Anderson Cancer Center, 1400 Pressler Street, Houston, TX 77030, Phone: 713-745-9283, E-mail: ychen26@mdanderson.org; Pei-Yong Shi, The University of Texas Medical Branch, 301 University Blvd., Galveston TX 77550, Phone: 409-772-1725, E-mail: peshi@UTMB.edu.

Supplementary information includes

- **Supplementary Figure 1-13 and their related figure legends**

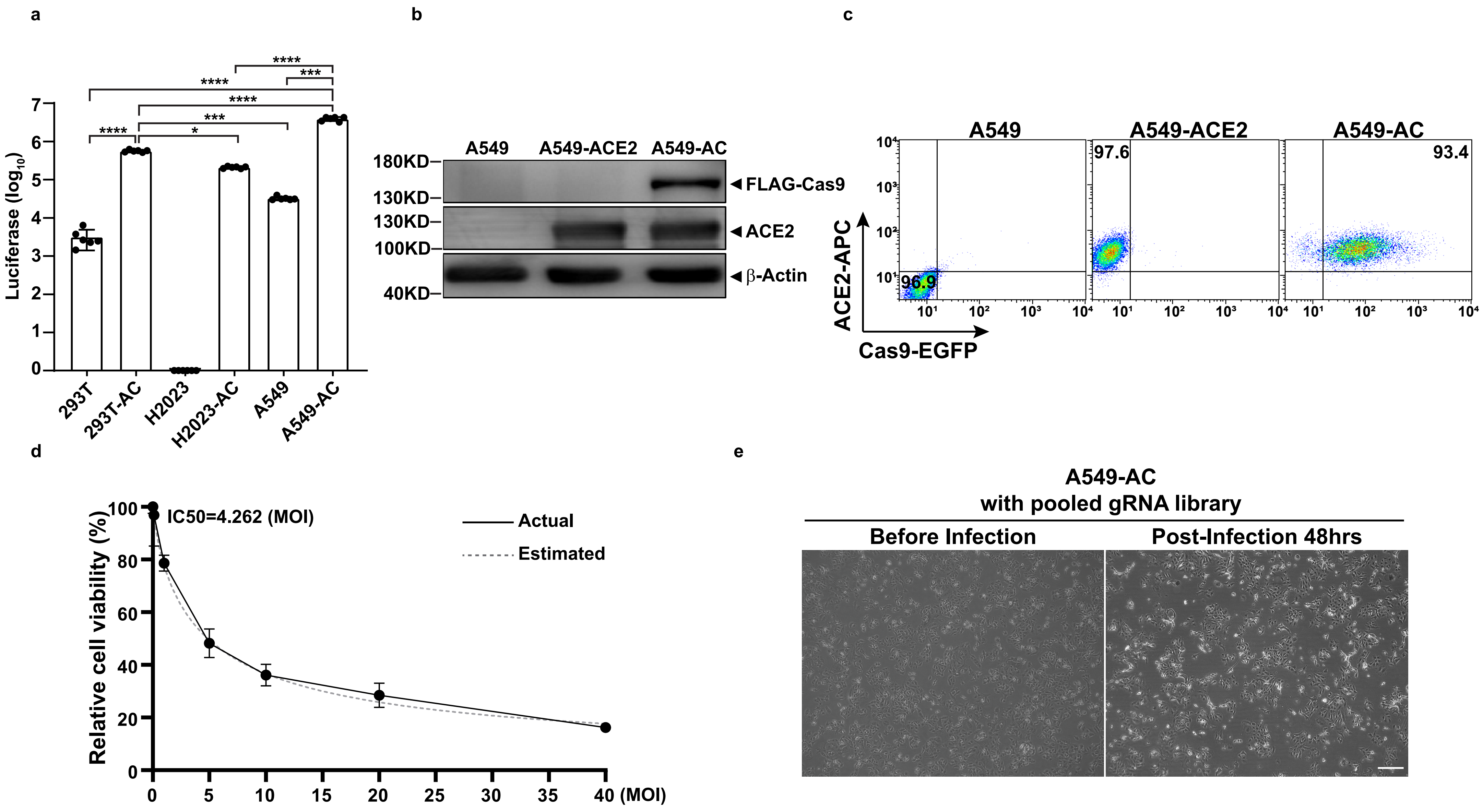
a
b
c
d
e
Luciferase (log10)
293T
293T-AC
H2023
H2023-AC
A549
A549-AC
****
***
*
180KD
130KD
100KD
40KD
A549
A549-ACE2
A549-AC
FLAG-Cas9
ACE2
β-Actin
ACE2-APC
Cas9-EGFP
96.9
97.6
93.4
Relative cell viability (%)
IC50=4.262 (MOI)
Actual
Estimated
(MOI)
A549-AC
with pooled gRNA library
Before Infection
Post-Infection 48hrs

Supplementary Figure 1. Establishment of genome-wide CRISPR/Cas9 dropout screens for SARS-CoV-2 infection (a) Evaluation of permissiveness of human epithelial cell lines for SARS-CoV-2 infection. Human epithelial cells were infected with recombinant SARS-CoV-2-Nluc at MOI=0.2 and luciferase signals were measured at 24-h post-infection. At least two independent experiments were performed. Data were analyzed using one-way ANOVA followed by Tukey's post hoc test were presented as mean values ± SD; n = 6 biologically independent samples. (b) Expression of ACE2 and Cas9 expression in A549-AC cells detected by western blot analysis. Anti-human ACE2 and anti-FLAG antibodies were used to determine the level of ACE and FLAG-tagged Cas9, respectively. (c) Expression levels of ACE2 and Cas9 in A549-AC cells detected by flow cytometry. The expression of GFP, a surrogate reporter for Cas9, was used to determine Cas9 expression. (d) Dose effect of SARS-CoV-2 on CPE. A549-AC cells were infected with different MOIs (ranging from 0.1 to 40) of recombinant SARS-CoV-2. 48 hours after infection, the viability of infected cells was measured. A four-parameter nonlinear regression method was used to generate the estimated dose-response curve and to calculate the MOI for 50% of cell lysis. (e) Representative images of cells before and after viral infection (scale bar 12.5μm, magnification 400×). Typical bright field images of pooled A549-AC cells with the gRNA library were illustrated before (left panel) and after (right panel) SARS-CoV-2 infection. Samples were triplicated in experiments. The comparisons with statistical significance were indicated. Source data and exact *p*-values are provided in a Source data file. *$p<0.05$; ***$p<0.001$; ****$p<0.0001$.

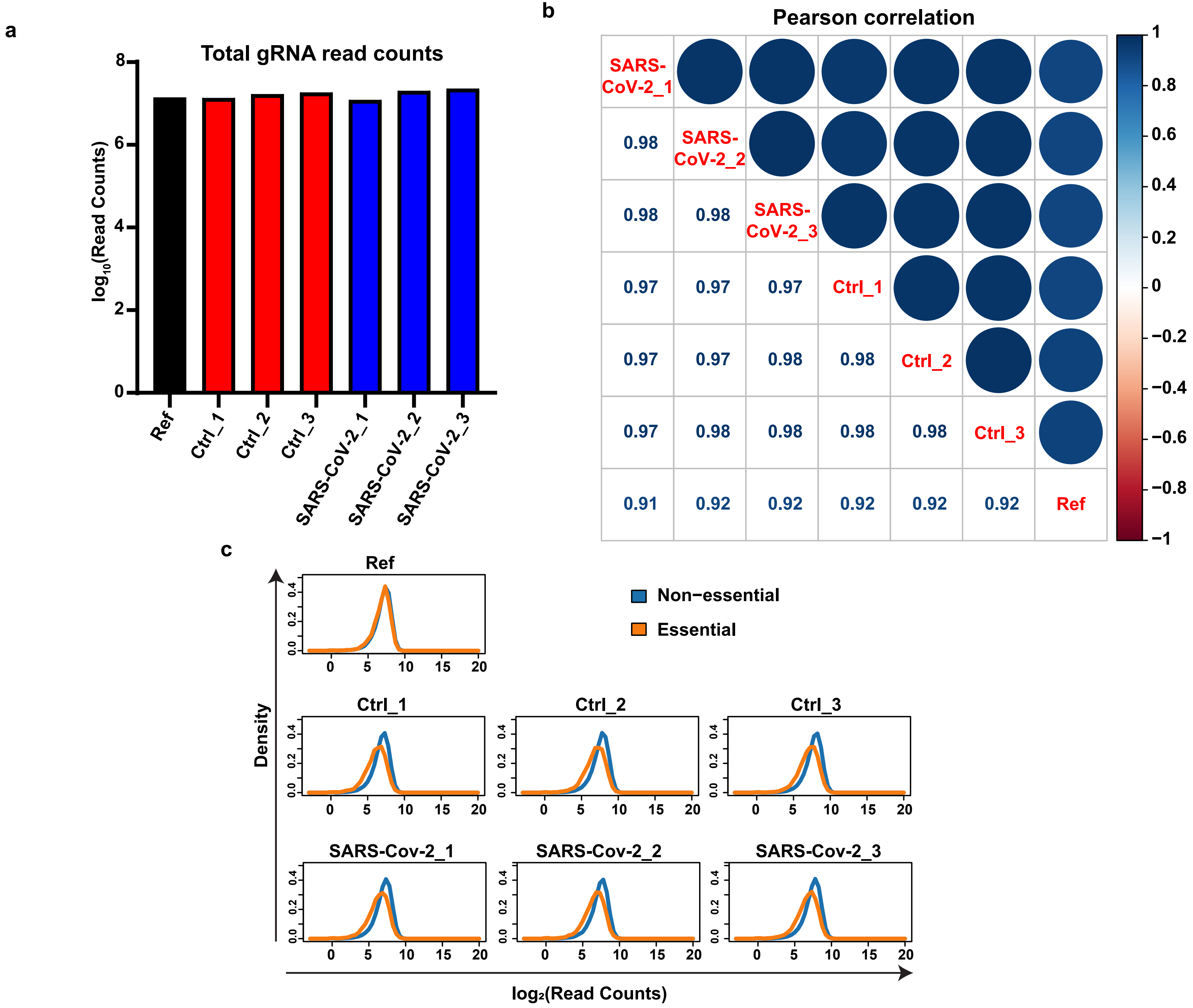
a
Total gRNA read counts
log10(Read Counts)
0
2
4
6
8
Ref
Ctrl_1
Ctrl_2
Ctrl_3
SARS-CoV-2_1
SARS-CoV-2_2
SARS-CoV-2_3
b
Pearson correlation
SARS-CoV-2_1
SARS-CoV-2_2
SARS-CoV-2_3
Ctrl_1
Ctrl_2
Ctrl_3
Ref
0.98
0.98 0.98
0.97 0.97 0.97
0.97 0.97 0.98 0.98
0.97 0.98 0.98 0.98 0.98
0.91 0.92 0.92 0.92 0.92 0.92
1
0.8
0.6
0.4
0.2
0
−0.2
−0.4
−0.6
−0.8
−1
c
Ref
Ctrl_1
Ctrl_2
Ctrl_3
SARS-Cov-2_1
SARS-Cov-2_2
SARS-Cov-2_3
Non−essential
Essential
Density
0.0
0.2
0.4
0
5
10
15
20
log2(Read Counts)

Supplementary Figure 2. Quality evaluation of results from the genome-wide CRISPR dropout screen. (a) Raw read counts of total gRNAs in samples collected from the genome-wide CRISPR dropout screen. (b) Correlations of gRNA abundance across experimental samples. Pairwise Pearson correlation analyses were performed between two distinct samples. (c) Distribution of gRNAs targeting essential and non-essential genes in the reference sample (upper panel), the control samples (middle panel), and the SARS-CoV-2 infected samples (bottom panel).

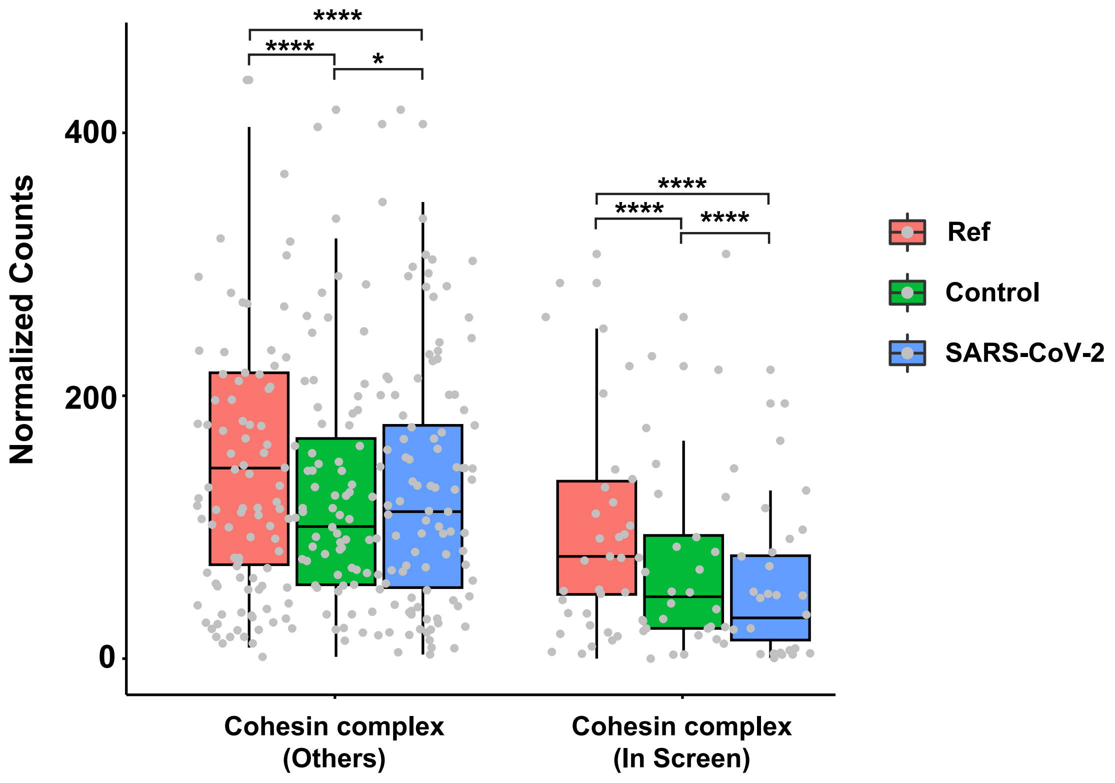
****
****
*
****
****
****
400
200
0
Normalized Counts
Cohesin complex
(Others)
Cohesin complex
(In Screen)
Ref
Control
SARS-CoV-2

Supplementary Figure 3. Changes in abundance of gRNAs targeting cohesin-related genes in the SARS-CoV-2 screen. Cohesin-related genes are into two categories: Inscreen (gRNAs targeting genes with statistically significant depletion in the SARS-CoV-2 group) and Others (gRNAs targeting genes without statistically significant depletion in the SARS-CoV-2 group). Boxplots of normalized gRNA counts of each category in three types of samples: the Ref samples, the control samples, and the SARS-CoV-2 samples were shown. Data were analyzed using Wilcoxon signed-rank test and presented as mean values ± SD; Source data and exact *p*-values are provided in a Source data file. *$p<0.05$; ***$p<0.001$; ****$p<0.0001$.

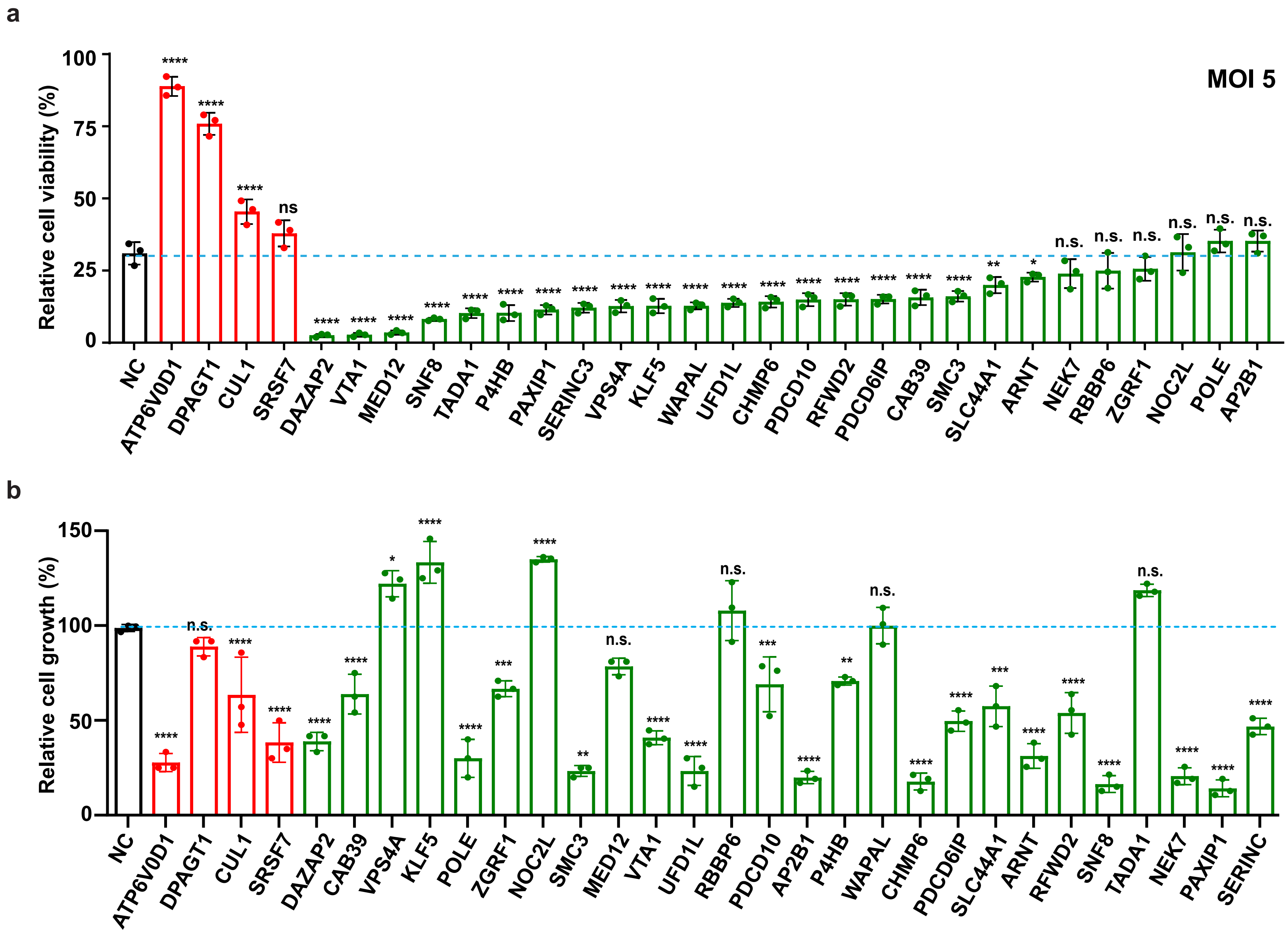
a
MOI 5
Relative cell viability (%)
100
75
50
25
0
NC
ATP6V0D1
DPAGT1
CUL1
SRSF7
DAZAP2
VTA1
MED12
SNF8
TADA1
P4HB
PAXIP1
SERINC3
VPS4A
KLF5
WAPAL
UFD1L
CHMP6
PDCD10
RFWD2
PDCD6IP
CAB39
SMC3
SLC44A1
ARNT
NEK7
RBBP6
ZGRF1
NOC2L
POLE
AP2B1
b
Relative cell growth (%)
150
100
50
0
NC
ATP6V0D1
DPAGT1
CUL1
SRSF7
DAZAP2
CAB39
VPS4A
KLF5
POLE
ZGRF1
NOC2L
SMC3
MED12
VTA1
UFD1L
RBBP6
PDCD10
AP2B1
P4HB
WAPAL
CHMP6
PDCD6IP
SLC44A1
ARNT
RFWD2
SNF8
TADA1
NEK7
PAXIP1
SERINC

Supplementary Figure 4. Phenotypes of knocking out putative host factors in lung epithelial cells. (a) Effects of knocking out putative host factors on CPE at the high MOI condition. A549-AC cell lines expressing related gRNAs were infected with SARS-CoV-2 at MOI=5. The cell viability was measured at 48 hours post-infection. (b) Effects of knocking out putative host factors on *in vitro* growth. Equal numbers of genetically modified A549-AC cells were seeded and cultured for 48 hours *in vitro*. The relative changes in cell numbers of A549-AC cells with gene-specific KO were calculated by normalizing with cell numbers of A549-AC cells expressing non-targeting gRNA. At least two independent experiments were performed. Data were analyzed using one-way ANOVA followed by Dunnett's post hoc test and were presented as mean values ± SD; n = 3 biologically independent samples. Source data and exact *p*-values are provided in a Source data file. *p<0.05; **p<0.01; ***p<0.001; ****p<0.0001. n.s., not significant.

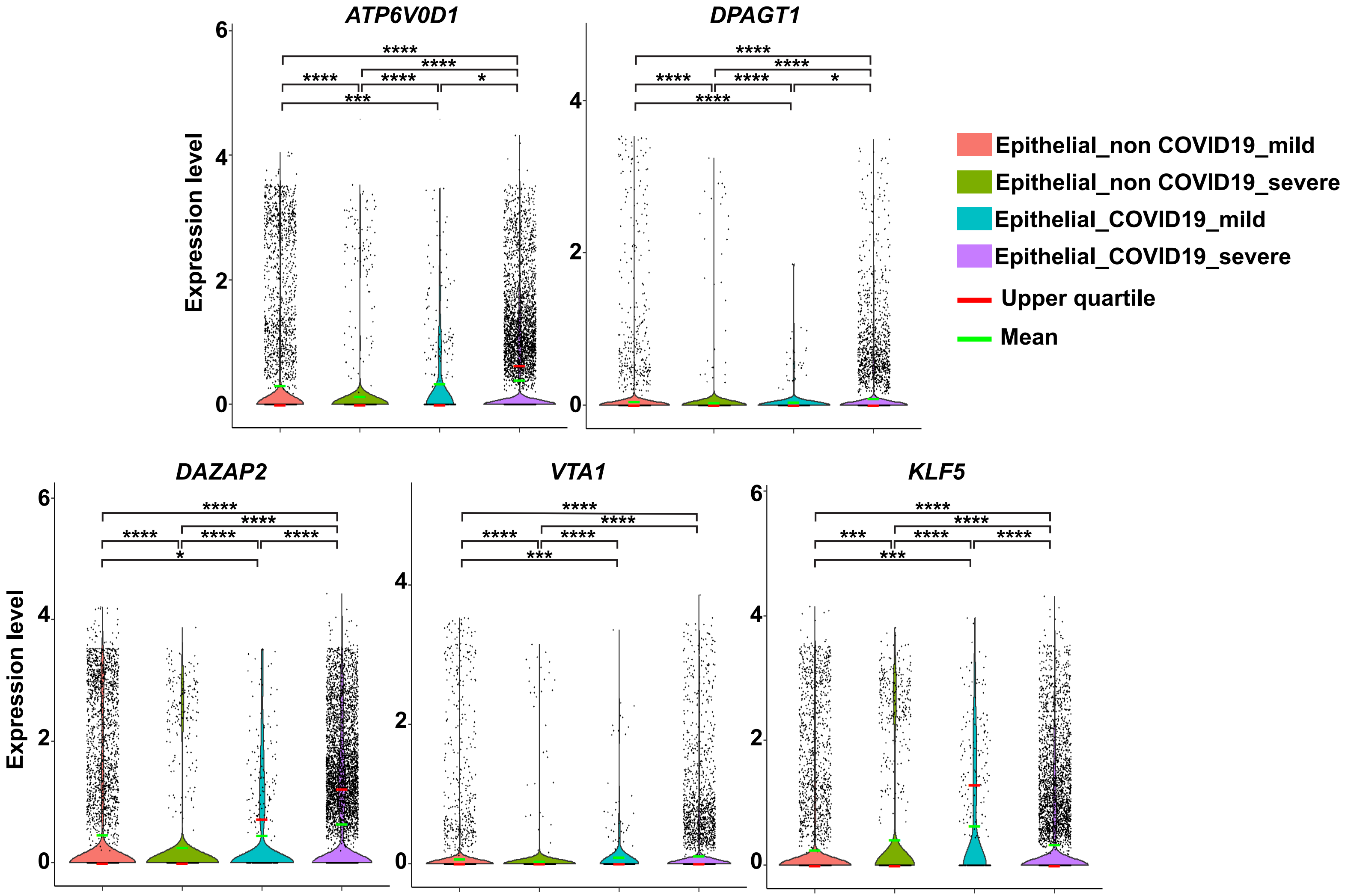
ATP6V0D1
DPAGT1
DAZAP2
VTA1
KLF5
Expression level
Epithelial_non COVID19_mild
Epithelial_non COVID19_severe
Epithelial_COVID19_mild
Epithelial_COVID19_severe
Upper quartile
Mean

Supplementary Figure 5. Comparisons of expression levels of identified host factors in lung epithelial cells from pneumonia patients. The mRNA expression levels of two pro-viral factors (*ATP6V0D1*and *DPAGT1*) and three anti-viral factors (*DAZAP2*, *VTA1*, and *KLF5*) in epithelial cells in bronchoalveolar lavage fluids were extracted from published datasets. Patients were stratified by their diagnosis (COVID-19 and non-COVID-19) and severity (mild and severe). Log-transformed mRNA counts of each host factor in lung epithelial cells from different patient groups were illustrated. The comparisons with statistical significance were indicated in the method. Source data and exact *p*-values are provided in a Source data file. *$p<0.05$; ***$p<0.001$; ****$p<0.0001$.

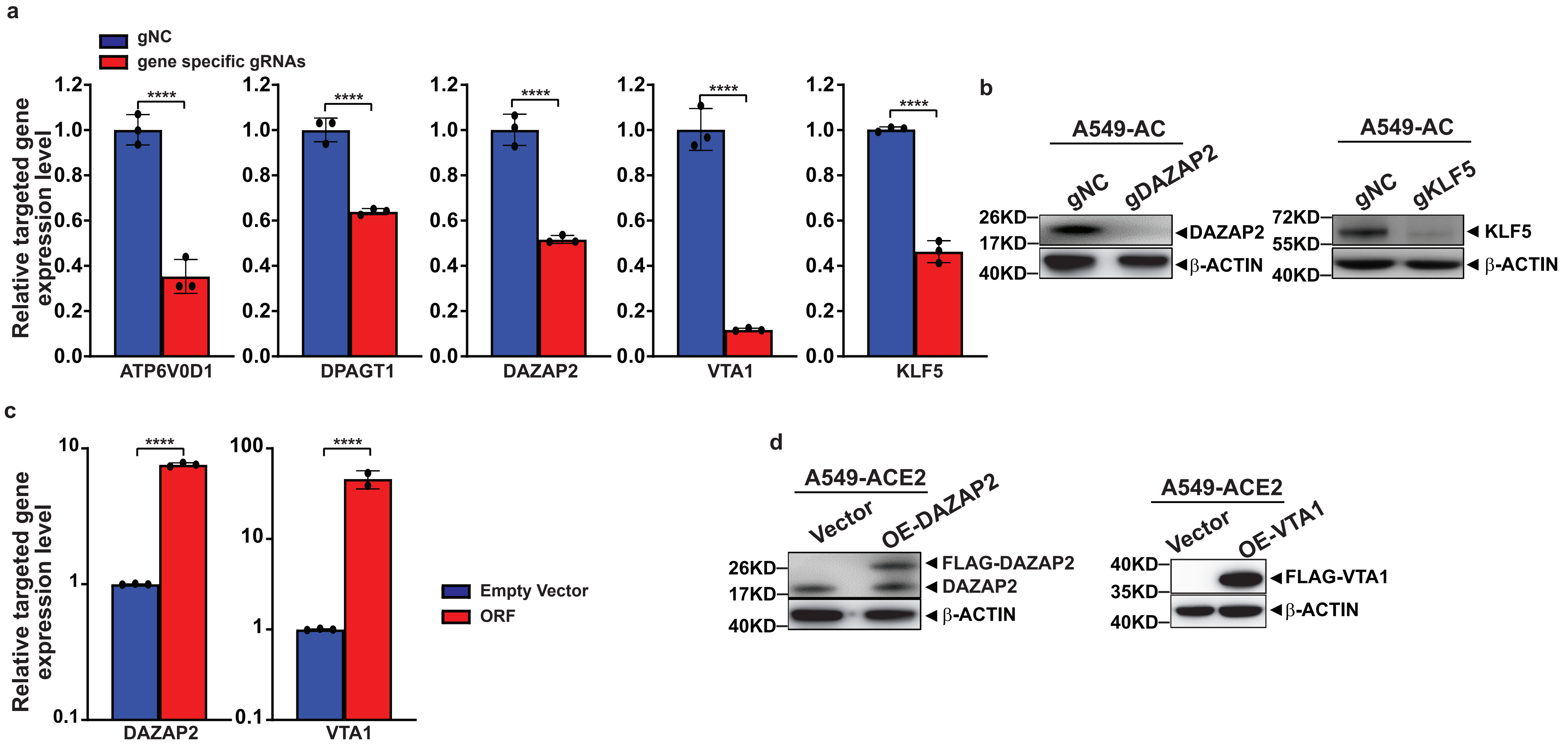
a
gNC
gene specific gRNAs
Relative targeted gene expression level
1.2
1.0
0.8
0.6
0.4
0.2
0.0
****
ATP6V0D1
DPAGT1
DAZAP2
VTA1
KLF5
b
A549-AC
gNC
gDAZAP2
26KD
17KD
40KD
DAZAP2
β-ACTIN
A549-AC
gNC
gKLF5
72KD
55KD
40KD
KLF5
β-ACTIN
c
Relative targeted gene expression level
10
1
0.1
100
****
DAZAP2
VTA1
Empty Vector
ORF
d
A549-ACE2
Vector
OE-DAZAP2
26KD
17KD
40KD
FLAG-DAZAP2
DAZAP2
β-ACTIN
A549-ACE2
Vector
OE-VTA1
40KD
35KD
40KD
FLAG-VTA1
β-ACTIN

Supplementary Figure 6. Successful gene-specific perturbations in A549-AC cells. (a) Inhibition of gene-of-interests in A549-AC cells by gene-specific gRNAs at mRNA level. (b) Inhibition of gene-of-interests in A549-AC cells by gene-specific gRNAs at protein level. (c) Increased mRNA expression of gene-of-interests in gene-specific overexpression (OE) A549-AC cells. (d) Increased protein expression of gene-of-interests in gene-specific OE A549-AC cells. The comparisons with statistical significance were indicated. At least two independent experiments were performed. Data were analyzed using unpaired T-test with two tails were presented as mean values ± SD; n = 3 biologically independent samples. Source data and exact *p*-values are provided in a Source data file. ***$p<0.001$; ****$p<0.0001$.

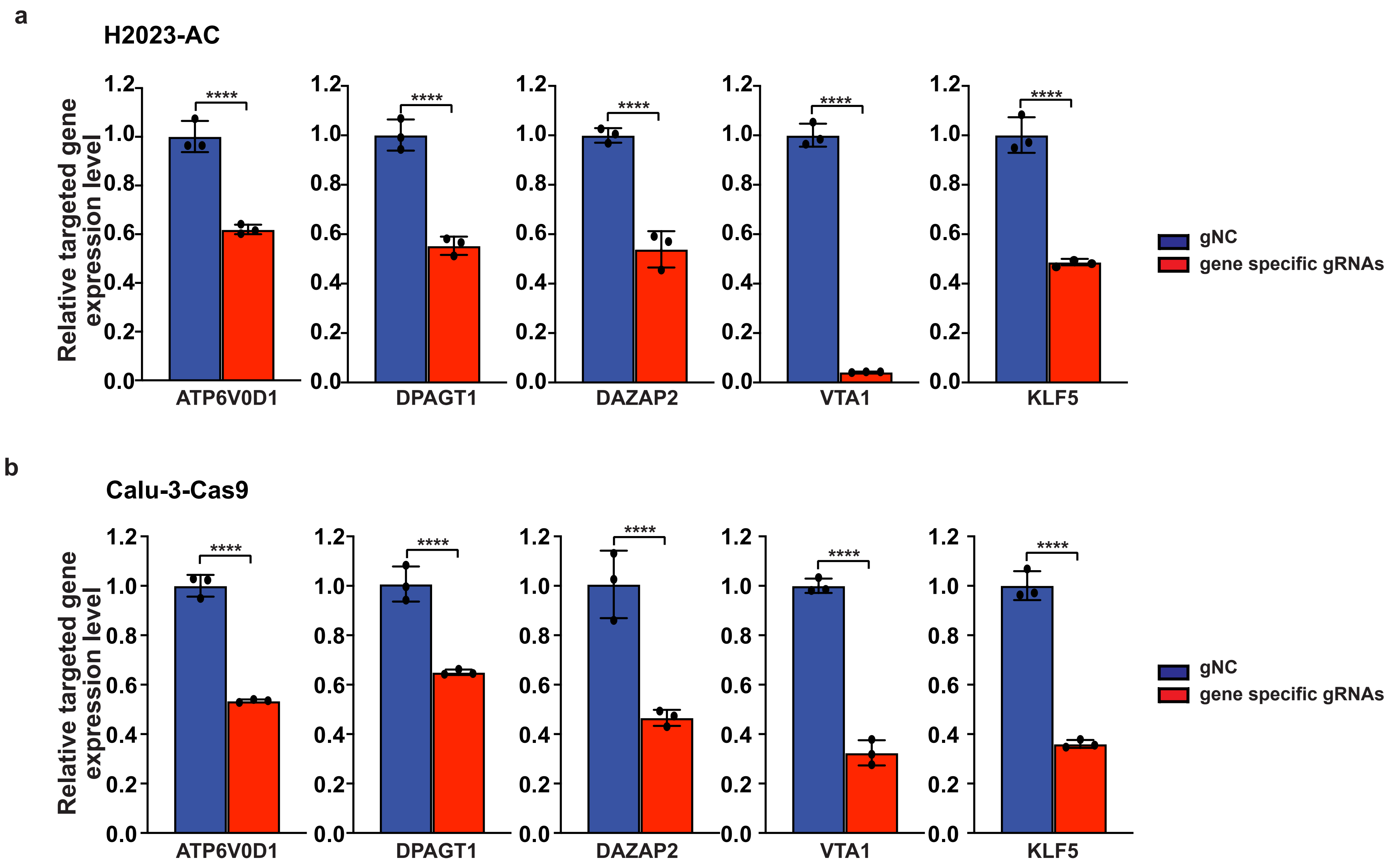
a
H2023-AC
Relative targeted gene expression level
1.2
1.0
0.8
0.6
0.4
0.2
0.0
****
ATP6V0D1
DPAGT1
DAZAP2
VTA1
KLF5
gNC
gene specific gRNAs
b
Calu-3-Cas9
Relative targeted gene expression level
****
ATP6V0D1
DPAGT1
DAZAP2
VTA1
KLF5
gNC
gene specific gRNAs

Supplementary Figure 7. Successful gene-specific perturbations in H2023-AC and Calu-3-Cas9 cells. Inhibition of gene-of-interests in H2023-AC cells (a) and Calu-3-Cas9 cells (b) by gene-specific gRNAs at mRNA level were determined by RT-PCR. The comparisons with statistical significance were indicated. At least two independent experiments were performed. Data were analyzed using unpaired T-test with two tails were presented as mean values ± SD; n = 3 biologically independent samples. Source data and exact *p*-values are provided in a Source data file. ***$p<0.001$; ****$p<0.0001$.

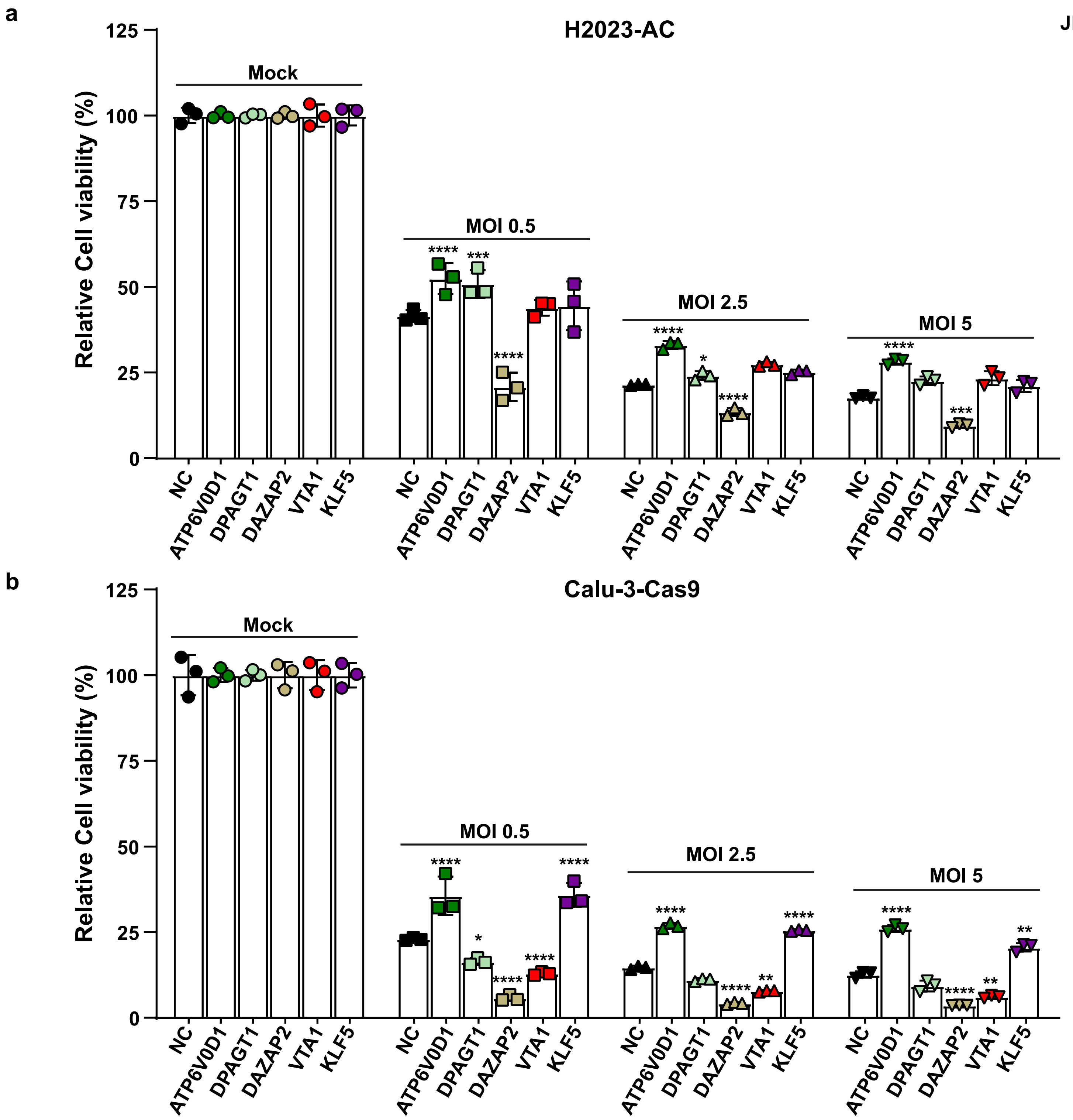
a
H2023-AC
b
Calu-3-Cas9
Relative Cell viability (%)
0
25
50
75
100
125
Mock
MOI 0.5
MOI 2.5
MOI 5
NC
ATP6V0D1
DPAGT1
DAZAP2
VTA1
KLF5

Supplementary Figure 8. Effects of perturbation of selected host factors on CPE induced by SARS-CoV-2 in H2023-AC and Calu-3-Cas9 cells. (a-b) Putative five host factors including two pro-viral factors (*ATP6V0D1, DPAGT1)* and three anti-viral factors (*DAZAP2, VTA1*, *KLF5)* were knocked out in H2023-AC and Calu-3-Cas9 cells. Genetically modified H2023-AC cells (a) and Calu-3-Cas9 cells (b) were infected with recombinant SARS-CoV-2 at MOI=0.5, 2.5, and 5 for 48 hours. H2023-AC and Calu-3-Cas9 cells expressing a non-targeting gRNA (NC) served as control cells. Data were normalized using the viability of corresponding cells at mock conditions. At least two independent experiments were performed. Data were analyzed using one-way ANOVA followed by Dunnett's post hoc test and were presented as mean values ± SD; n = 3 biologically independent samples. Source data and exact *p*-values are provided in a Source data file. *$p<0.05$; **$p<0.01$; ***$p<0.001$; ****$p<0.0001$.

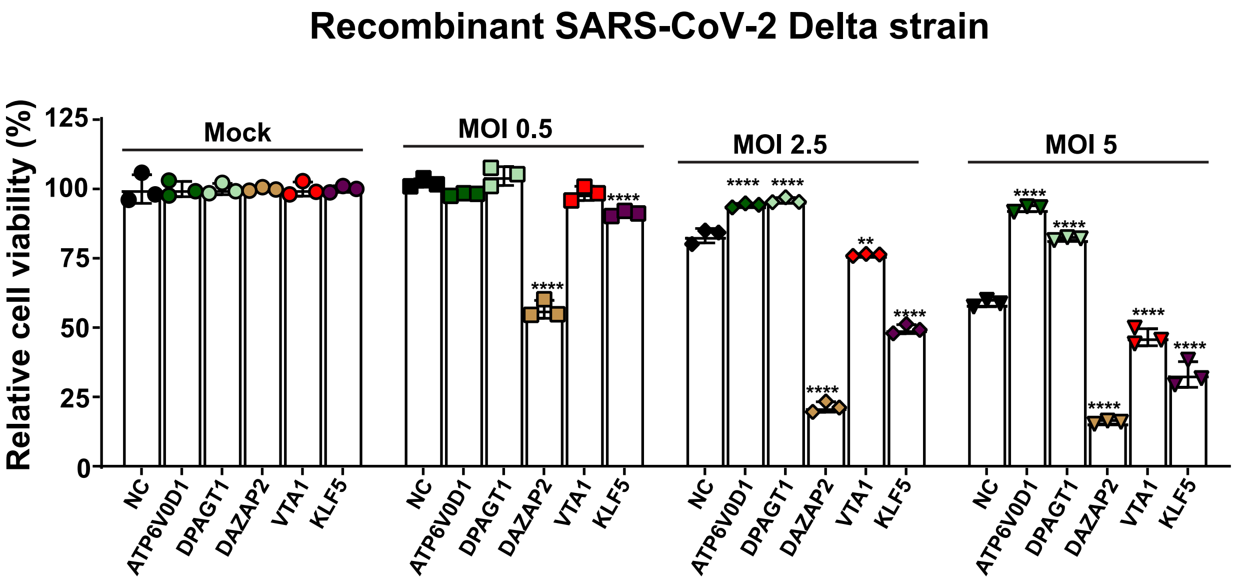
Recombinant SARS-CoV-2 Delta strain
Relative cell viability (%)
125
100
75
50
25
0
Mock
MOI 0.5
MOI 2.5
MOI 5
****
**
NC
ATP6V0D1
DPAGT1
DAZAP2
VTA1
KLF5

Supplementary Figure 9. Effects of perturbation of selected host factors on CPE induced by the Delta strain of SARS-CoV-2. A549-AC cells with or without genetic KO were infected with recombinant SARS-CoV-2 (Delta strain) at MOI=0.5, 2.5, and 5 for 48 hours. A549-AC cells expressing a non-targeting gRNA (NC) served as control cells. Data were normalized using the viability of corresponding cells at mock conditions. At least two independent experiments were performed. Data were analyzed using one-way ANOVA followed by Dunnett's post hoc test and were presented as mean values ± SD; n = 3 biologically independent samples. Source data and exact *p*-values are provided in a Source data file. ***p*<0.01; ****p*<0.001; *****p*<0.0001.

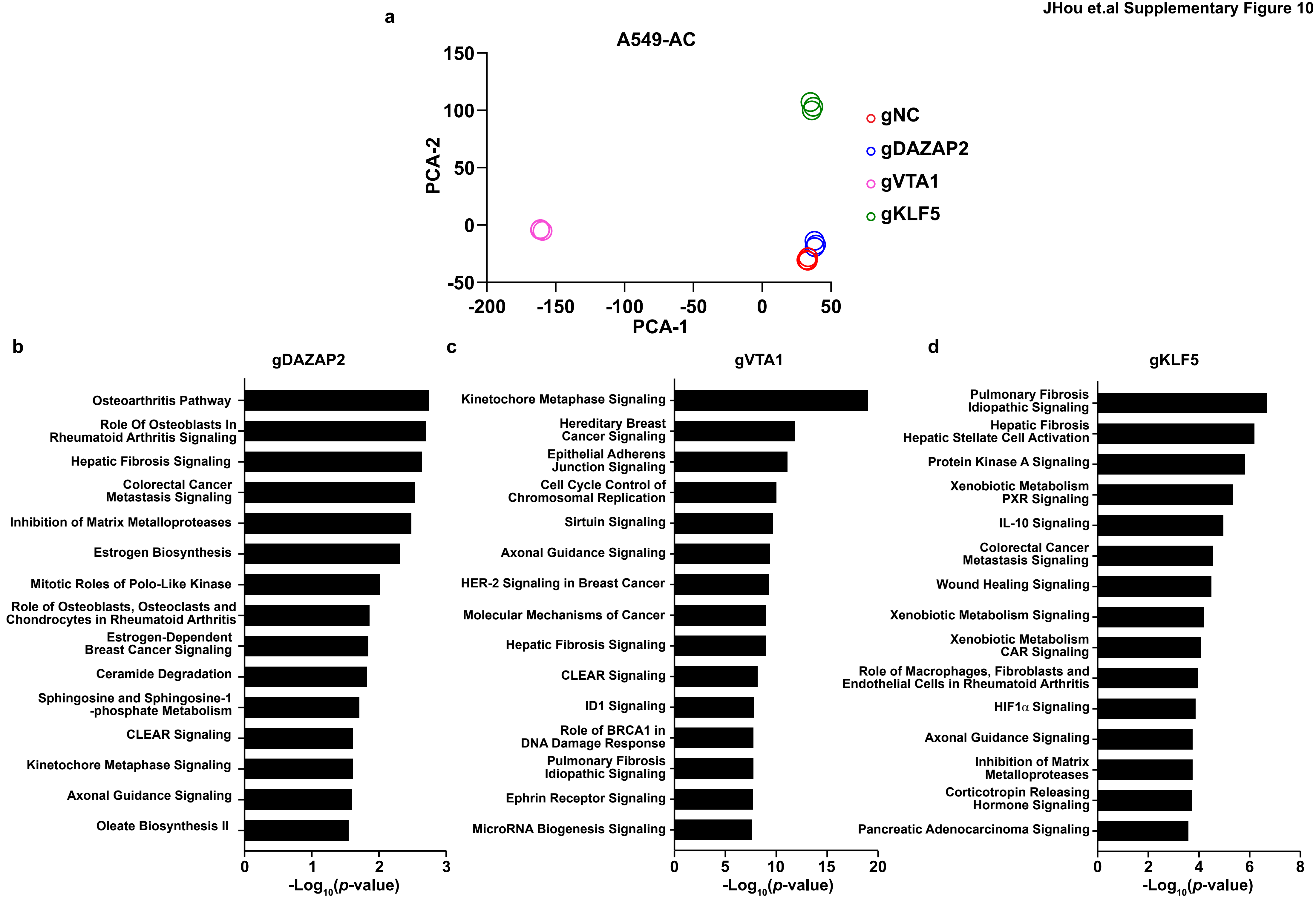
a
A549-AC
PCA-2
PCA-1
150
100
50
0
-50
-200
-150
-100
-50
0
50
gNC
gDAZAP2
gVTA1
gKLF5
b
gDAZAP2
Osteoarthritis Pathway
Role Of Osteoblasts In Rheumatoid Arthritis Signaling
Hepatic Fibrosis Signaling
Colorectal Cancer Metastasis Signaling
Inhibition of Matrix Metalloproteases
Estrogen Biosynthesis
Mitotic Roles of Polo-Like Kinase
Role of Osteoblasts, Osteoclasts and Chondrocytes in Rheumatoid Arthritis
Estrogen-Dependent Breast Cancer Signaling
Ceramide Degradation
Sphingosine and Sphingosine-1 -phosphate Metabolism
CLEAR Signaling
Kinetochore Metaphase Signaling
Axonal Guidance Signaling
Oleate Biosynthesis II
0
1
2
3
-Log10(p-value)
c
gVTA1
Kinetochore Metaphase Signaling
Hereditary Breast Cancer Signaling
Epithelial Adherens Junction Signaling
Cell Cycle Control of Chromosomal Replication
Sirtuin Signaling
Axonal Guidance Signaling
HER-2 Signaling in Breast Cancer
Molecular Mechanisms of Cancer
Hepatic Fibrosis Signaling
CLEAR Signaling
ID1 Signaling
Role of BRCA1 in DNA Damage Response
Pulmonary Fibrosis Idiopathic Signaling
Ephrin Receptor Signaling
MicroRNA Biogenesis Signaling
0
5
10
15
20
-Log10(p-value)
d
gKLF5
Pulmonary Fibrosis Idiopathic Signaling
Hepatic Fibrosis Hepatic Stellate Cell Activation
Protein Kinase A Signaling
Xenobiotic Metabolism PXR Signaling
IL-10 Signaling
Colorectal Cancer Metastasis Signaling
Wound Healing Signaling
Xenobiotic Metabolism Signaling
Xenobiotic Metabolism CAR Signaling
Role of Macrophages, Fibroblasts and Endothelial Cells in Rheumatoid Arthritis
HIF1α Signaling
Axonal Guidance Signaling
Inhibition of Matrix Metalloproteases
Corticotropin Releasing Hormone Signaling
Pancreatic Adenocarcinoma Signaling
0
2
4
6
8
-Log10(p-value)

Supplementary Figure 10. Molecular characterization of DAZAP2 /VTA1/KLF5-KO cells. (a) PCA analysis reveals distinct transcriptomic profiles among A549-AC with and without genetic KO. (b-d) IPA of results from the unique DEGs among DAZAP2-KO (b), VTA1-KO (c), and KLF5-KO (d) cells. The top 15 enriched canonical pathways displaying statistical significance were listed. *p*-values for each gene set were calculated by using a Right-Tailed Fisher's Exact Test. Source data and exact *p*-values are provided in a Source data file.

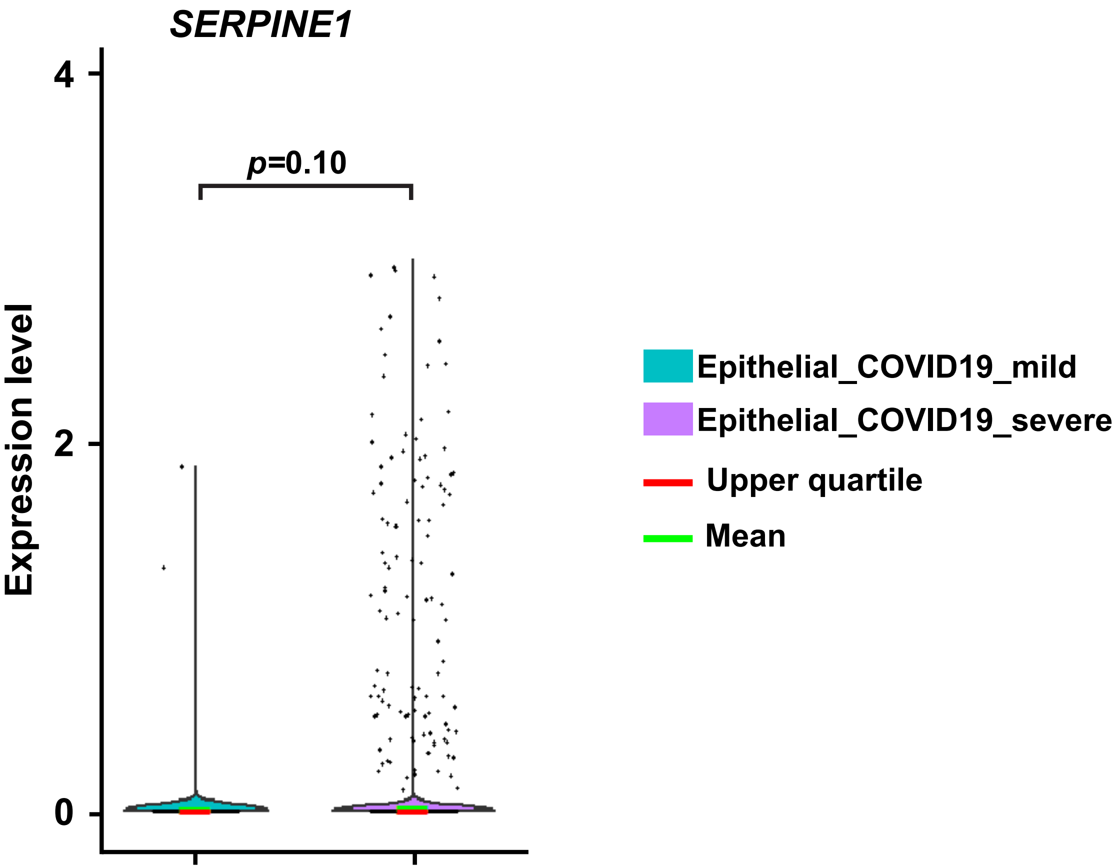
SERPINE1
Expression level
4
2
0
p=0.10
Epithelial_COVID19_mild
Epithelial_COVID19_severe
Upper quartile
Mean

Supplementary Figure 11. Levels of *SERPINE1* expression in lung epithelial cells from COVID-19 patients. The mRNA expression levels of *SERPINE1* in epithelial cells in bronchoalveolar lavage fluids were extracted from published scRNA-Seq datasets as described in Supplementary Figure 5. The box plot was made by using Log-transformed mRNA counts in lung epithelial cells from different patient groups. The comparisons with statistical significance were indicated in the method.

JHou et.al Supplementary Figure 12

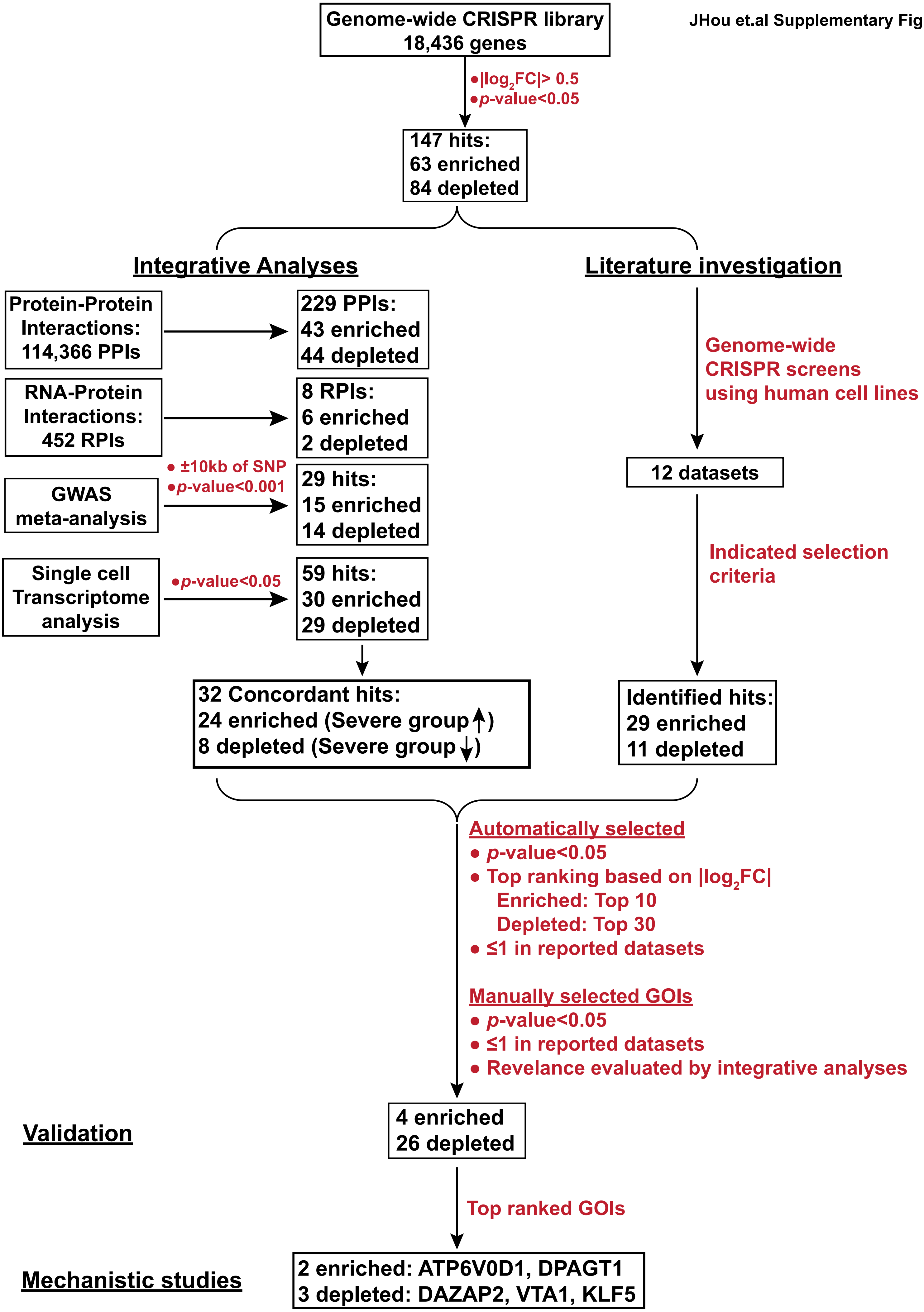

Supplementary Figure 12. Summary of experimental procedures including screens, integrative analysis, candidate selection, and validation.

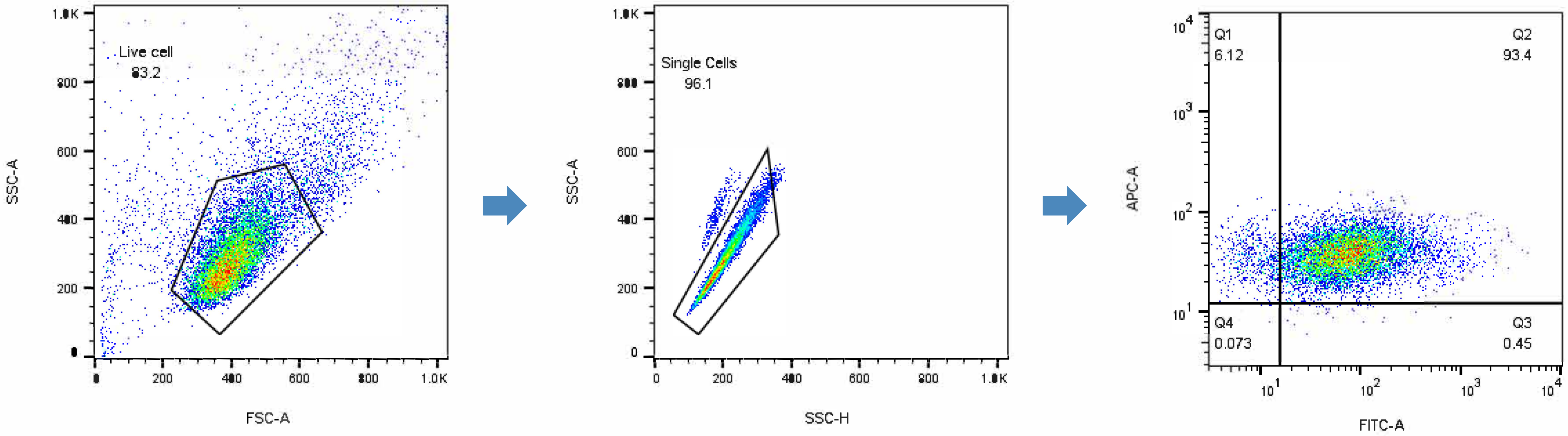

Supplementary Figure 13. Gating strategy of flow cytometry.
