# Supplementary material for: Integrated multi-omics analyses identify anti-viral host factors and pathways controlling SARS-CoV-2 infection
Source: Nat Commun. 2024 Jan 2;15:109. doi: 10.1038/s41467-023-44175-1 (PMC10761986; doi:10.1038/s41467-023-44175-1)
Supplement: Supplementary file 2 — Description of Additional Supplementary Files [file 41467_2023_44175_MOESM2_ESM.pdf]

Description of Additional Supplementary Files

Supplementary Data 1. List of pro-viral host factors identified from SARS-CoV-2 dropout screens. Pro-viral hits from genome-wide CRISPR screen were based on a fold change cut-off in gRNA abundance ($Log_2FC \geq 0.5$) and a statistically significant threshold of $p<0.05$.

Supplementary Data 2. List of anti-viral host factors identified from SARS-CoV-2 dropout screens. Anti-viral hits from genome-wide CRISPR screen were based on a fold change cut-off in gRNA abundance ($Log_2FC \leq -0.5$) and a statistically significant threshold of $p<0.05$.

Supplementary Data 3. Summary of published genome-wide gRNA SARS-CoV-2 screens. Publicly available datasets from genome-wide CRISPR screens based on the CPE of SARS-CoV-2 on human epithelial cells were extracted and used to evaluate the performance of identified hits. The criteria of each dataset for candidate selection were listed.

Supplementary Data 4. Performance of 30 identified host factors in two independent validation experiments. Results of 30 identified host hits from two rounds of independent validation experiments were listed.

Supplementary Data 5. List of DNA sequences to generate gRNAs targeting 30 identified host factors. Protospacer and flanking sequences for the plasmid constructions of gRNAs targeting 30 identified host factors were listed.

Supplementary Data 6. List of primers used in RT-PCR analysis. Primer sequences of identified host factors for RT-PCR validation were listed.

Supplementary Software. The supplementary software package includes two individual files and one folder.

1. The file named "README" provides the detailed information including "system requirements", "Installation", "Demo" and "Instructions for use".
2. The file named "BioinformaticsAnalysis.Screen-GWAS-Interactome.code.txt" provides a summary of customer codes for the bioinformatics analysis of CRISPR dropout screen, genome-wide association study (GWAS) and protein-protein/RNA interactions.

3. The folder named “scRNA-patient-epithelial” provides the data availability of scRNA-seq datasets and a summary of customer codes for the scRNA-seq analysis of gene expressions from patient airway epithelial cells.
