# Supplementary material for: Integrated multi-omics analyses identify anti-viral host factors and pathways controlling SARS-CoV-2 infection
Source: Nat Commun. 2024 Jan 2;15:109. doi: 10.1038/s41467-023-44175-1 (PMC10761986; doi:10.1038/s41467-023-44175-1)
Supplement: Supplementary file 9 — Supplementary Software [file 41467_2023_44175_MOESM9_ESM.zip › code summary/scRNA-patient-epithelial/supp-fig-4.nb.html]

Supplementary Figure 4


Code 

- Show All Code
- Hide All Code
- Download Rmd

# Supplementary Figure 4

## This notebook generates figures for Supplementary Figure 4

## Preparation

You will need to download raw data from: https://lambrechtslab.sites.vib.be/en/immune-atlas

The page requires login to download data, but any Google account is
good. After login, there will be multiple links for downloading. The
2069-Allcells.counts.rds is available in the “Clustering all cells”
section. The 2076-OnlineTMEM106B.rds is in the “TMEM106B” section.

## Data preparation


```
data <- readRDS("2076-OnlineTMEM106B.rds")
data@meta.data
```


```
obj <- readRDS("2069-Allcells.counts.rds")
meta.data <- data@meta.data[colnames(obj), ]
obj <- CreateSeuratObject(obj, meta.data = meta.data)
obj
```


```
An object of class Seurat 
33538 features across 65166 samples within 1 assay 
Active assay: RNA (33538 features, 0 variable features)
```


```
obj$Patient <- stringr::str_match(colnames(obj), "^(.+)_")[, 2]
table(obj$Patient, obj$PatientType)
```


```
           ICU  mild severe  Ward
  BAL001     0     0   1884     0
  BAL002     0  3775      0     0
  BAL003     0  2117      0     0
  BAL009     0  1212      0     0
  BAL010     0  6395      0     0
  BAL011     0   448      0     0
  BAL012     0     0    498     0
  BAL013     0     0   1950     0
  BAL014     0     0   1939     0
  BAL015     0     0   1989     0
  BAL016     0     0   1399     0
  BAL017     0  1128      0     0
  BAL018     0   504      0     0
  BAL019     0 11574      0     0
  BAL020     3     0   1405     0
  BAL021     0     0   2593     0
  BAL022    55     0   1427     0
  BAL023    71     0   1153     0
  BAL024     0     0   2267     0
  BAL025     0     0   1175     0
  BAL026   115     0   1996     0
  BAL027    40     0   1447     0
  BAL028     0  1183      0     0
  BAL029     0  1278      0     0
  BAL030     0   310      0     0
  BAL031     0     0   3795     0
  BAL032     0     0    582     0
  BAL033    31     0    974     0
  BAL034     0     0   1199     0
  BAL035     0     0   1144     0
  BAL036     0   230      0     0
  BAL037     0   896      0    18
  BAL038     0  1097      0     0
  BAL039     0     0   1017     0
  BAL040    10     0    843     0
```


```
obj$PatientType2 <- obj$PatientType
obj$PatientType2[obj$PatientType2 == 'ICU'] <- 'severe'
obj$PatientType2[obj$PatientType2 == 'Ward'] <- 'mild'
```


```
obj <- NormalizeData(obj, verbose = F)
```

## Generate figures


```
library(Seurat)
library(cowplot)
library(grid)
library(gridExtra)
library(ggplot2)
library(lattice)


median.stat <- function(x){
   out <- quantile(x, probs = c(0.5))
   names(out) <- c("ymed")
   return(out) 
}

median.stat75 <- function(x){
   out <- quantile(x, probs = c(0.75))
   names(out) <- c("ymed")
   return(out) 
}

`%ni%`<- Negate(`%in%`)
```


```
library(ggpubr)
```


```
Warning: 程辑包‘ggpubr’是用R版本4.2.1 来建造的
载入程辑包：‘ggpubr’

The following object is masked from ‘package:cowplot’:

    get_legend
```


```
goi <- read.table("positive-candidates.txt")$V1

data <- obj[, obj$Domain == 'Epithelial']

data$Domain_disease_patient_type <- paste(data$Domain, data$Disease, data$PatientType2, sep='_')
data$Domain_disease_patient_type <- factor(data$Domain_disease_patient_type, 
                                          levels = c("Epithelial_control_mild", "Epithelial_control_severe",
                                                     "Epithelial_COVID19_mild", "Epithelial_COVID19_severe"
                                                     ))
Idents(data) <- 'Domain_disease_patient_type'

# goi <- readxl::read_xlsx("../../../data/Covid19/wauters-cell-research/2021-12-22 Candidate  list-COVID.XLSX", sheet = 2)
#cnt = 0
for (g in goi) {
  if (g %in% rownames(data)){
    png(paste0('positive/', g, '.png'), height = 6, width = 4, units = "in", res = 200)

    my_comparisons <- list( c("Epithelial_control_mild", "Epithelial_control_severe"), 
                        c("Epithelial_control_severe", "Epithelial_COVID19_mild"), 
                        c("Epithelial_COVID19_mild", "Epithelial_COVID19_severe"),
                        c("Epithelial_control_mild", "Epithelial_COVID19_mild"), 
                        c("Epithelial_control_severe", "Epithelial_COVID19_severe"),
                        c("Epithelial_control_mild", "Epithelial_COVID19_severe"))

    feature = g

    data2 <- data.frame(label = Idents(data), expression = data@assays$RNA@data[g, ])
    ymax = max(data2$expression)

    my_comparisons_y <- (c(1.5, 1.05, 1.15, 1.25, 1.4, 1.7) + 0.3) / 1.3
    print(
      ggplot(data=data2, aes(x = label, y=expression, fill = label)) + 
        geom_violin() +
        geom_point(position = position_jitter(seed = 1, width = 0.2), size = 0.1) + ylim(-0.01, ymax * 1.72) + 
        stat_summary(fun.y = mean, geom='point', size = 5, colour = "green", shape = 95) + 
        stat_summary(fun.y = median.stat75, geom='point', size = 5, colour = "red", shape = 95) + 
        stat_compare_means(comparisons = my_comparisons, label.y = ymax * my_comparisons_y, method = "wilcox.test", size = 4) +
        stat_compare_means(label.y = ymax * 1.7, label.x = 1.3, size = 4) +
        theme_classic() + theme(legend.position = "none") + ggtitle(g) + theme(axis.text.x = element_text(angle = 45, hjust = 1))
    )
    dev.off()
  } else {
    print(paste(g, "not found."))
  }
  #cnt = cnt + 1
  #if (cnt > 5) break
}
```


```
Warning: `fun.y` is deprecated. Use `fun` instead.Warning: `fun.y` is deprecated. Use `fun` instead.
```


```
library(ggpubr)

goi <- read.table("negative-candidates.txt")$V1

data <- obj[, obj$Domain == 'Epithelial']

data$Domain_disease_patient_type <- paste(data$Domain, data$Disease, data$PatientType2, sep='_')
data$Domain_disease_patient_type <- factor(data$Domain_disease_patient_type, 
                                          levels = c("Epithelial_control_mild", "Epithelial_control_severe",
                                                     "Epithelial_COVID19_mild", "Epithelial_COVID19_severe"
                                                     ))
Idents(data) <- 'Domain_disease_patient_type'

for (g in goi) {
  if (g %in% rownames(data)){
    png(paste0('negative/', g, '.png'), height = 6, width = 4, units = "in", res = 200)

    my_comparisons <- list( c("Epithelial_control_mild", "Epithelial_control_severe"), 
                        c("Epithelial_control_severe", "Epithelial_COVID19_mild"), 
                        c("Epithelial_COVID19_mild", "Epithelial_COVID19_severe"),
                        c("Epithelial_control_mild", "Epithelial_COVID19_mild"), 
                        c("Epithelial_control_severe", "Epithelial_COVID19_severe"),
                        c("Epithelial_control_mild", "Epithelial_COVID19_severe"))

    feature = g

    data2 <- data.frame(label = Idents(data), expression = data@assays$RNA@data[g, ])
    ymax = max(data2$expression)

    my_comparisons_y <- (c(1.5, 1.05, 1.15, 1.25, 1.4, 1.7) + 0.3) / 1.3
    print(
      ggplot(data=data2, aes(x = label, y=expression, fill = label)) + 
        geom_violin() +
        geom_point(position = position_jitter(seed = 1, width = 0.2), size = 0.1) + ylim(-0.01, ymax * 1.72) + 
        stat_summary(fun.y = mean, geom='point', size = 5, colour = "green", shape = 95) + 
        stat_summary(fun.y = median.stat75, geom='point', size = 5, colour = "red", shape = 95) + 
        stat_compare_means(comparisons = my_comparisons, label.y = ymax * my_comparisons_y, method = "wilcox.test", size = 4) +
        stat_compare_means(label.y = ymax * 1.7, label.x = 1.3, size = 4) +
        theme_classic() + theme(legend.position = "none") + ggtitle(g) + theme(axis.text.x = element_text(angle = 45, hjust = 1))
    )
    dev.off()
  } else {
    print(paste(g, "not found."))
  }
  #cnt = cnt + 1
  #if (cnt > 5) break
}
```


```
Warning: `fun.y` is deprecated. Use `fun` instead.Warning: `fun.y` is deprecated. Use `fun` instead.
```


```
[1] "RFWD2 not found."
[1] "CCDC101 not found."
[1] "WAPAL not found."
[1] "UFD1L not found."
```

LS0tDQp0aXRsZTogIlN1cHBsZW1lbnRhcnkgRmlndXJlIDQiDQpvdXRwdXQ6IGh0bWxfbm90ZWJvb2sNCi0tLQ0KDQojIyBUaGlzIG5vdGVib29rIGdlbmVyYXRlcyBmaWd1cmVzIGZvciBTdXBwbGVtZW50YXJ5IEZpZ3VyZSA0DQoNCiMjIFByZXBhcmF0aW9uDQpZb3Ugd2lsbCBuZWVkIHRvIGRvd25sb2FkIHJhdyBkYXRhIGZyb206IGh0dHBzOi8vbGFtYnJlY2h0c2xhYi5zaXRlcy52aWIuYmUvZW4vaW1tdW5lLWF0bGFzDQoNClRoZSBwYWdlIHJlcXVpcmVzIGxvZ2luIHRvIGRvd25sb2FkIGRhdGEsIGJ1dCBhbnkgR29vZ2xlIGFjY291bnQgaXMgZ29vZC4gQWZ0ZXIgbG9naW4sIHRoZXJlIHdpbGwgYmUgbXVsdGlwbGUgbGlua3MgZm9yIGRvd25sb2FkaW5nLiBUaGUgMjA2OS1BbGxjZWxscy5jb3VudHMucmRzIGlzIGF2YWlsYWJsZSBpbiB0aGUgIkNsdXN0ZXJpbmcgYWxsIGNlbGxzIiBzZWN0aW9uLiBUaGUgMjA3Ni1PbmxpbmVUTUVNMTA2Qi5yZHMgaXMgaW4gdGhlICJUTUVNMTA2QiIgc2VjdGlvbi4NCg0KIyMgRGF0YSBwcmVwYXJhdGlvbg0KDQpgYGB7cn0NCmRhdGEgPC0gcmVhZFJEUygiMjA3Ni1PbmxpbmVUTUVNMTA2Qi5yZHMiKQ0KZGF0YUBtZXRhLmRhdGENCg0Kb2JqIDwtIHJlYWRSRFMoIjIwNjktQWxsY2VsbHMuY291bnRzLnJkcyIpDQptZXRhLmRhdGEgPC0gZGF0YUBtZXRhLmRhdGFbY29sbmFtZXMob2JqKSwgXQ0Kb2JqIDwtIENyZWF0ZVNldXJhdE9iamVjdChvYmosIG1ldGEuZGF0YSA9IG1ldGEuZGF0YSkNCm9iag0KYGBgDQpgYGB7cn0NCm9iaiRQYXRpZW50IDwtIHN0cmluZ3I6OnN0cl9tYXRjaChjb2xuYW1lcyhvYmopLCAiXiguKylfIilbLCAyXQ0KdGFibGUob2JqJFBhdGllbnQsIG9iaiRQYXRpZW50VHlwZSkNCm9iaiRQYXRpZW50VHlwZTIgPC0gb2JqJFBhdGllbnRUeXBlDQpvYmokUGF0aWVudFR5cGUyW29iaiRQYXRpZW50VHlwZTIgPT0gJ0lDVSddIDwtICdzZXZlcmUnDQpvYmokUGF0aWVudFR5cGUyW29iaiRQYXRpZW50VHlwZTIgPT0gJ1dhcmQnXSA8LSAnbWlsZCcNCmBgYA0KDQoNCmBgYHtyfQ0Kb2JqIDwtIE5vcm1hbGl6ZURhdGEob2JqLCB2ZXJib3NlID0gRikNCmBgYA0KDQojIyBHZW5lcmF0ZSBmaWd1cmVzDQoNCmBgYHtyfQ0KbGlicmFyeShTZXVyYXQpDQpsaWJyYXJ5KGNvd3Bsb3QpDQpsaWJyYXJ5KGdyaWQpDQpsaWJyYXJ5KGdyaWRFeHRyYSkNCmxpYnJhcnkoZ2dwbG90MikNCmxpYnJhcnkobGF0dGljZSkNCg0KDQptZWRpYW4uc3RhdCA8LSBmdW5jdGlvbih4KXsNCiAgIG91dCA8LSBxdWFudGlsZSh4LCBwcm9icyA9IGMoMC41KSkNCiAgIG5hbWVzKG91dCkgPC0gYygieW1lZCIpDQogICByZXR1cm4ob3V0KSANCn0NCg0KbWVkaWFuLnN0YXQ3NSA8LSBmdW5jdGlvbih4KXsNCiAgIG91dCA8LSBxdWFudGlsZSh4LCBwcm9icyA9IGMoMC43NSkpDQogICBuYW1lcyhvdXQpIDwtIGMoInltZWQiKQ0KICAgcmV0dXJuKG91dCkgDQp9DQoNCmAlbmklYDwtIE5lZ2F0ZShgJWluJWApDQpgYGANCg0KDQpgYGB7cn0NCmxpYnJhcnkoZ2dwdWJyKQ0KDQoNCmdvaSA8LSByZWFkLnRhYmxlKCJwb3NpdGl2ZS1jYW5kaWRhdGVzLnR4dCIpJFYxDQoNCmRhdGEgPC0gb2JqWywgb2JqJERvbWFpbiA9PSAnRXBpdGhlbGlhbCddDQoNCmRhdGEkRG9tYWluX2Rpc2Vhc2VfcGF0aWVudF90eXBlIDwtIHBhc3RlKGRhdGEkRG9tYWluLCBkYXRhJERpc2Vhc2UsIGRhdGEkUGF0aWVudFR5cGUyLCBzZXA9J18nKQ0KZGF0YSREb21haW5fZGlzZWFzZV9wYXRpZW50X3R5cGUgPC0gZmFjdG9yKGRhdGEkRG9tYWluX2Rpc2Vhc2VfcGF0aWVudF90eXBlLCANCiAgICAgICAgICAgICAgICAgICAgICAgICAgICAgICAgICAgICAgICAgIGxldmVscyA9IGMoIkVwaXRoZWxpYWxfY29udHJvbF9taWxkIiwgIkVwaXRoZWxpYWxfY29udHJvbF9zZXZlcmUiLA0KICAgICAgICAgICAgICAgICAgICAgICAgICAgICAgICAgICAgICAgICAgICAgICAgICAgICAiRXBpdGhlbGlhbF9DT1ZJRDE5X21pbGQiLCAiRXBpdGhlbGlhbF9DT1ZJRDE5X3NldmVyZSINCiAgICAgICAgICAgICAgICAgICAgICAgICAgICAgICAgICAgICAgICAgICAgICAgICAgICAgKSkNCklkZW50cyhkYXRhKSA8LSAnRG9tYWluX2Rpc2Vhc2VfcGF0aWVudF90eXBlJw0KDQojIGdvaSA8LSByZWFkeGw6OnJlYWRfeGxzeCgiLi4vLi4vLi4vZGF0YS9Db3ZpZDE5L3dhdXRlcnMtY2VsbC1yZXNlYXJjaC8yMDIxLTEyLTIyIENhbmRpZGF0ZSAgbGlzdC1DT1ZJRC5YTFNYIiwgc2hlZXQgPSAyKQ0KI2NudCA9IDANCmZvciAoZyBpbiBnb2kpIHsNCiAgaWYgKGcgJWluJSByb3duYW1lcyhkYXRhKSl7DQogICAgcG5nKHBhc3RlMCgncG9zaXRpdmUvJywgZywgJy5wbmcnKSwgaGVpZ2h0ID0gNiwgd2lkdGggPSA0LCB1bml0cyA9ICJpbiIsIHJlcyA9IDIwMCkNCg0KICAgIG15X2NvbXBhcmlzb25zIDwtIGxpc3QoIGMoIkVwaXRoZWxpYWxfY29udHJvbF9taWxkIiwgIkVwaXRoZWxpYWxfY29udHJvbF9zZXZlcmUiKSwgDQogICAgICAgICAgICAgICAgICAgICAgICBjKCJFcGl0aGVsaWFsX2NvbnRyb2xfc2V2ZXJlIiwgIkVwaXRoZWxpYWxfQ09WSUQxOV9taWxkIiksIA0KICAgICAgICAgICAgICAgICAgICAgICAgYygiRXBpdGhlbGlhbF9DT1ZJRDE5X21pbGQiLCAiRXBpdGhlbGlhbF9DT1ZJRDE5X3NldmVyZSIpLA0KICAgICAgICAgICAgICAgICAgICAgICAgYygiRXBpdGhlbGlhbF9jb250cm9sX21pbGQiLCAiRXBpdGhlbGlhbF9DT1ZJRDE5X21pbGQiKSwgDQogICAgICAgICAgICAgICAgICAgICAgICBjKCJFcGl0aGVsaWFsX2NvbnRyb2xfc2V2ZXJlIiwgIkVwaXRoZWxpYWxfQ09WSUQxOV9zZXZlcmUiKSwNCiAgICAgICAgICAgICAgICAgICAgICAgIGMoIkVwaXRoZWxpYWxfY29udHJvbF9taWxkIiwgIkVwaXRoZWxpYWxfQ09WSUQxOV9zZXZlcmUiKSkNCg0KICAgIGZlYXR1cmUgPSBnDQoNCiAgICBkYXRhMiA8LSBkYXRhLmZyYW1lKGxhYmVsID0gSWRlbnRzKGRhdGEpLCBleHByZXNzaW9uID0gZGF0YUBhc3NheXMkUk5BQGRhdGFbZywgXSkNCiAgICB5bWF4ID0gbWF4KGRhdGEyJGV4cHJlc3Npb24pDQoNCg0KICAgIG15X2NvbXBhcmlzb25zX3kgPC0gKGMoMS41LCAxLjA1LCAxLjE1LCAxLjI1LCAxLjQsIDEuNykgKyAwLjMpIC8gMS4zDQogICAgcHJpbnQoDQogICAgICBnZ3Bsb3QoZGF0YT1kYXRhMiwgYWVzKHggPSBsYWJlbCwgeT1leHByZXNzaW9uLCBmaWxsID0gbGFiZWwpKSArIA0KICAgICAgICBnZW9tX3Zpb2xpbigpICsNCiAgICAgICAgZ2VvbV9wb2ludChwb3NpdGlvbiA9IHBvc2l0aW9uX2ppdHRlcihzZWVkID0gMSwgd2lkdGggPSAwLjIpLCBzaXplID0gMC4xKSArIHlsaW0oLTAuMDEsIHltYXggKiAxLjcyKSArIA0KICAgICAgICBzdGF0X3N1bW1hcnkoZnVuLnkgPSBtZWFuLCBnZW9tPSdwb2ludCcsIHNpemUgPSA1LCBjb2xvdXIgPSAiZ3JlZW4iLCBzaGFwZSA9IDk1KSArIA0KICAgICAgICBzdGF0X3N1bW1hcnkoZnVuLnkgPSBtZWRpYW4uc3RhdDc1LCBnZW9tPSdwb2ludCcsIHNpemUgPSA1LCBjb2xvdXIgPSAicmVkIiwgc2hhcGUgPSA5NSkgKyANCiAgICAgICAgc3RhdF9jb21wYXJlX21lYW5zKGNvbXBhcmlzb25zID0gbXlfY29tcGFyaXNvbnMsIGxhYmVsLnkgPSB5bWF4ICogbXlfY29tcGFyaXNvbnNfeSwgbWV0aG9kID0gIndpbGNveC50ZXN0Iiwgc2l6ZSA9IDQpICsNCiAgICAgICAgc3RhdF9jb21wYXJlX21lYW5zKGxhYmVsLnkgPSB5bWF4ICogMS43LCBsYWJlbC54ID0gMS4zLCBzaXplID0gNCkgKw0KICAgICAgICB0aGVtZV9jbGFzc2ljKCkgKyB0aGVtZShsZWdlbmQucG9zaXRpb24gPSAibm9uZSIpICsgZ2d0aXRsZShnKSArIHRoZW1lKGF4aXMudGV4dC54ID0gZWxlbWVudF90ZXh0KGFuZ2xlID0gNDUsIGhqdXN0ID0gMSkpDQogICAgKQ0KICAgIGRldi5vZmYoKQ0KICB9IGVsc2Ugew0KICAgIHByaW50KHBhc3RlKGcsICJub3QgZm91bmQuIikpDQogIH0NCiAgI2NudCA9IGNudCArIDENCiAgI2lmIChjbnQgPiA1KSBicmVhaw0KfQ0KYGBgDQoNCmBgYHtyfQ0KbGlicmFyeShnZ3B1YnIpDQoNCmdvaSA8LSByZWFkLnRhYmxlKCJuZWdhdGl2ZS1jYW5kaWRhdGVzLnR4dCIpJFYxDQoNCmRhdGEgPC0gb2JqWywgb2JqJERvbWFpbiA9PSAnRXBpdGhlbGlhbCddDQoNCmRhdGEkRG9tYWluX2Rpc2Vhc2VfcGF0aWVudF90eXBlIDwtIHBhc3RlKGRhdGEkRG9tYWluLCBkYXRhJERpc2Vhc2UsIGRhdGEkUGF0aWVudFR5cGUyLCBzZXA9J18nKQ0KZGF0YSREb21haW5fZGlzZWFzZV9wYXRpZW50X3R5cGUgPC0gZmFjdG9yKGRhdGEkRG9tYWluX2Rpc2Vhc2VfcGF0aWVudF90eXBlLCANCiAgICAgICAgICAgICAgICAgICAgICAgICAgICAgICAgICAgICAgICAgIGxldmVscyA9IGMoIkVwaXRoZWxpYWxfY29udHJvbF9taWxkIiwgIkVwaXRoZWxpYWxfY29udHJvbF9zZXZlcmUiLA0KICAgICAgICAgICAgICAgICAgICAgICAgICAgICAgICAgICAgICAgICAgICAgICAgICAgICAiRXBpdGhlbGlhbF9DT1ZJRDE5X21pbGQiLCAiRXBpdGhlbGlhbF9DT1ZJRDE5X3NldmVyZSINCiAgICAgICAgICAgICAgICAgICAgICAgICAgICAgICAgICAgICAgICAgICAgICAgICAgICAgKSkNCklkZW50cyhkYXRhKSA8LSAnRG9tYWluX2Rpc2Vhc2VfcGF0aWVudF90eXBlJw0KDQpmb3IgKGcgaW4gZ29pKSB7DQogIGlmIChnICVpbiUgcm93bmFtZXMoZGF0YSkpew0KICAgIHBuZyhwYXN0ZTAoJ25lZ2F0aXZlLycsIGcsICcucG5nJyksIGhlaWdodCA9IDYsIHdpZHRoID0gNCwgdW5pdHMgPSAiaW4iLCByZXMgPSAyMDApDQoNCiAgICBteV9jb21wYXJpc29ucyA8LSBsaXN0KCBjKCJFcGl0aGVsaWFsX2NvbnRyb2xfbWlsZCIsICJFcGl0aGVsaWFsX2NvbnRyb2xfc2V2ZXJlIiksIA0KICAgICAgICAgICAgICAgICAgICAgICAgYygiRXBpdGhlbGlhbF9jb250cm9sX3NldmVyZSIsICJFcGl0aGVsaWFsX0NPVklEMTlfbWlsZCIpLCANCiAgICAgICAgICAgICAgICAgICAgICAgIGMoIkVwaXRoZWxpYWxfQ09WSUQxOV9taWxkIiwgIkVwaXRoZWxpYWxfQ09WSUQxOV9zZXZlcmUiKSwNCiAgICAgICAgICAgICAgICAgICAgICAgIGMoIkVwaXRoZWxpYWxfY29udHJvbF9taWxkIiwgIkVwaXRoZWxpYWxfQ09WSUQxOV9taWxkIiksIA0KICAgICAgICAgICAgICAgICAgICAgICAgYygiRXBpdGhlbGlhbF9jb250cm9sX3NldmVyZSIsICJFcGl0aGVsaWFsX0NPVklEMTlfc2V2ZXJlIiksDQogICAgICAgICAgICAgICAgICAgICAgICBjKCJFcGl0aGVsaWFsX2NvbnRyb2xfbWlsZCIsICJFcGl0aGVsaWFsX0NPVklEMTlfc2V2ZXJlIikpDQoNCiAgICBmZWF0dXJlID0gZw0KDQogICAgZGF0YTIgPC0gZGF0YS5mcmFtZShsYWJlbCA9IElkZW50cyhkYXRhKSwgZXhwcmVzc2lvbiA9IGRhdGFAYXNzYXlzJFJOQUBkYXRhW2csIF0pDQogICAgeW1heCA9IG1heChkYXRhMiRleHByZXNzaW9uKQ0KDQoNCiAgICBteV9jb21wYXJpc29uc195IDwtIChjKDEuNSwgMS4wNSwgMS4xNSwgMS4yNSwgMS40LCAxLjcpICsgMC4zKSAvIDEuMw0KICAgIHByaW50KA0KICAgICAgZ2dwbG90KGRhdGE9ZGF0YTIsIGFlcyh4ID0gbGFiZWwsIHk9ZXhwcmVzc2lvbiwgZmlsbCA9IGxhYmVsKSkgKyANCiAgICAgICAgZ2VvbV92aW9saW4oKSArDQogICAgICAgIGdlb21fcG9pbnQocG9zaXRpb24gPSBwb3NpdGlvbl9qaXR0ZXIoc2VlZCA9IDEsIHdpZHRoID0gMC4yKSwgc2l6ZSA9IDAuMSkgKyB5bGltKC0wLjAxLCB5bWF4ICogMS43MikgKyANCiAgICAgICAgc3RhdF9zdW1tYXJ5KGZ1bi55ID0gbWVhbiwgZ2VvbT0ncG9pbnQnLCBzaXplID0gNSwgY29sb3VyID0gImdyZWVuIiwgc2hhcGUgPSA5NSkgKyANCiAgICAgICAgc3RhdF9zdW1tYXJ5KGZ1bi55ID0gbWVkaWFuLnN0YXQ3NSwgZ2VvbT0ncG9pbnQnLCBzaXplID0gNSwgY29sb3VyID0gInJlZCIsIHNoYXBlID0gOTUpICsgDQogICAgICAgIHN0YXRfY29tcGFyZV9tZWFucyhjb21wYXJpc29ucyA9IG15X2NvbXBhcmlzb25zLCBsYWJlbC55ID0geW1heCAqIG15X2NvbXBhcmlzb25zX3ksIG1ldGhvZCA9ICJ3aWxjb3gudGVzdCIsIHNpemUgPSA0KSArDQogICAgICAgIHN0YXRfY29tcGFyZV9tZWFucyhsYWJlbC55ID0geW1heCAqIDEuNywgbGFiZWwueCA9IDEuMywgc2l6ZSA9IDQpICsNCiAgICAgICAgdGhlbWVfY2xhc3NpYygpICsgdGhlbWUobGVnZW5kLnBvc2l0aW9uID0gIm5vbmUiKSArIGdndGl0bGUoZykgKyB0aGVtZShheGlzLnRleHQueCA9IGVsZW1lbnRfdGV4dChhbmdsZSA9IDQ1LCBoanVzdCA9IDEpKQ0KICAgICkNCiAgICBkZXYub2ZmKCkNCiAgfSBlbHNlIHsNCiAgICBwcmludChwYXN0ZShnLCAibm90IGZvdW5kLiIpKQ0KICB9DQogICNjbnQgPSBjbnQgKyAxDQogICNpZiAoY250ID4gNSkgYnJlYWsNCn0NCmBgYA0KDQo=
