# Supplementary material for: Integrated multi-omics analyses identify anti-viral host factors and pathways controlling SARS-CoV-2 infection
Source: Nat Commun. 2024 Jan 2;15:109. doi: 10.1038/s41467-023-44175-1 (PMC10761986; doi:10.1038/s41467-023-44175-1)
Supplement: Supplementary file 9 — Supplementary Software [file 41467_2023_44175_MOESM9_ESM.zip › code summary/scRNA-patient-epithelial/figure-2d-source-data.html]

Figure 2d source data generation


Code 

- Show All Code
- Hide All Code
- Download Rmd

# Figure 2d source data generation

## Preparation

You will need to download raw data from: https://lambrechtslab.sites.vib.be/en/immune-atlas

The page requires login to download data, but any Google account is
good. After login, there will be multiple links for downloading. The
2069-Allcells.counts.rds is available in the “Clustering all cells”
section. The 2076-OnlineTMEM106B.rds is in the “TMEM106B” section.


```
library(Seurat)
```


```
Registered S3 methods overwritten by 'htmltools':
  method               from         
  print.html           tools:rstudio
  print.shiny.tag      tools:rstudio
  print.shiny.tag.list tools:rstudio
Registered S3 method overwritten by 'data.table':
  method           from
  print.data.table     
Registered S3 method overwritten by 'htmlwidgets':
  method           from         
  print.htmlwidget tools:rstudio
Attaching SeuratObject
Attaching sp
```


```
data <- readRDS("2076-OnlineTMEM106B.rds")
data@meta.data
```


```
obj <- readRDS("2069-Allcells.counts.rds")
meta.data <- data@meta.data[colnames(obj), ]
obj <- CreateSeuratObject(obj, meta.data = meta.data)
obj
```


```
An object of class Seurat 
33538 features across 65166 samples within 1 assay 
Active assay: RNA (33538 features, 0 variable features)
```


```
obj$Patient <- stringr::str_match(colnames(obj), "^(.+)_")[, 2]
table(obj$Patient, obj$PatientType)
```


```
           ICU  mild severe  Ward
  BAL001     0     0   1884     0
  BAL002     0  3775      0     0
  BAL003     0  2117      0     0
  BAL009     0  1212      0     0
  BAL010     0  6395      0     0
  BAL011     0   448      0     0
  BAL012     0     0    498     0
  BAL013     0     0   1950     0
  BAL014     0     0   1939     0
  BAL015     0     0   1989     0
  BAL016     0     0   1399     0
  BAL017     0  1128      0     0
  BAL018     0   504      0     0
  BAL019     0 11574      0     0
  BAL020     3     0   1405     0
  BAL021     0     0   2593     0
  BAL022    55     0   1427     0
  BAL023    71     0   1153     0
  BAL024     0     0   2267     0
  BAL025     0     0   1175     0
  BAL026   115     0   1996     0
  BAL027    40     0   1447     0
  BAL028     0  1183      0     0
  BAL029     0  1278      0     0
  BAL030     0   310      0     0
  BAL031     0     0   3795     0
  BAL032     0     0    582     0
  BAL033    31     0    974     0
  BAL034     0     0   1199     0
  BAL035     0     0   1144     0
  BAL036     0   230      0     0
  BAL037     0   896      0    18
  BAL038     0  1097      0     0
  BAL039     0     0   1017     0
  BAL040    10     0    843     0
```


```
obj$PatientType2 <- obj$PatientType
obj$PatientType2[obj$PatientType2 == 'ICU'] <- 'severe'
obj$PatientType2[obj$PatientType2 == 'Ward'] <- 'mild'
```


```
obj <- NormalizeData(obj, verbose = F)
```


```
library(Seurat)
library(cowplot)
library(grid)
library(gridExtra)
library(ggplot2)
library(lattice)


median.stat <- function(x){
   out <- quantile(x, probs = c(0.5))
   names(out) <- c("ymed")
   return(out) 
}

median.stat75 <- function(x){
   out <- quantile(x, probs = c(0.75))
   names(out) <- c("ymed")
   return(out) 
}

`%ni%`<- Negate(`%in%`)
```

## Generate DEG tables


```
data <- obj
goi <- read.table("negative-candidates.txt")$V1
#cnt = 0

data$Domain_disease_patient_type <- paste(data$Domain, data$Disease, data$PatientType2, sep='_')
data$Domain_disease_patient_type <- factor(data$Domain_disease_patient_type, 
                                          levels = c("Epithelial_control_mild", "Epithelial_control_severe",
                                                     "Epithelial_COVID19_mild", "Epithelial_COVID19_severe"
                                                     ))
Idents(data) <- 'Domain_disease_patient_type'

    my_comparisons <- list( c("Epithelial_control_mild", "Epithelial_control_severe"), 
                        c("Epithelial_control_severe", "Epithelial_COVID19_mild"), 
                        c("Epithelial_COVID19_mild", "Epithelial_COVID19_severe"),
                        c("Epithelial_control_mild", "Epithelial_COVID19_mild"), 
                        c("Epithelial_control_severe", "Epithelial_COVID19_severe"),
                        c("Epithelial_control_mild", "Epithelial_COVID19_severe"))

df <- data.frame(nrows = goi)
rownames(df) <- goi

for (cmp in my_comparisons) {
  df[[paste0(cmp[1], '_vs_', cmp[2], '_p')]] <- NA
  df[[paste0(cmp[1], '_vs_', cmp[2], '_p_adj')]] <- NA
  df[[paste0(cmp[1], '_vs_', cmp[2], '_logfc')]] <- NA
  
  mutual_features <- intersect(goi, rownames(data))
  
  deg <- FindMarkers(data, cmp[1], cmp[2], features = mutual_features, slot = "data",
                        min.cells.group = 0, 
                        min.cells.feature = 0,
                        min.pct = -1,
                        logfc.threshold = -1,
                        only.pos = FALSE)
  
  df[mutual_features, paste0(cmp[1], '_vs_', cmp[2], '_p')] <- deg[mutual_features, 'p_val']
  df[mutual_features, paste0(cmp[1], '_vs_', cmp[2], '_p_adj')] <- deg[mutual_features, 'p_val_adj']
  df[mutual_features, paste0(cmp[1], '_vs_', cmp[2], '_logfc')] <- deg[mutual_features, 'avg_log2FC']
}
```


```
  |                                                  | 0 % ~calculating  
  |+                                                 | 1 % ~03s          
  |++                                                | 2 % ~02s          
  |++                                                | 4 % ~02s          
  |+++                                               | 5 % ~02s          
  |++++                                              | 6 % ~02s          
  |++++                                              | 8 % ~02s          
  |+++++                                             | 9 % ~02s          
  |+++++                                             | 10% ~02s          
  |++++++                                            | 11% ~01s          
  |+++++++                                           | 12% ~01s          
  |+++++++                                           | 14% ~01s          
  |++++++++                                          | 15% ~01s          
  |+++++++++                                         | 16% ~01s          
  |+++++++++                                         | 18% ~01s          
  |++++++++++                                        | 19% ~01s          
  |++++++++++                                        | 20% ~01s          
  |+++++++++++                                       | 21% ~01s          
  |++++++++++++                                      | 22% ~01s          
  |++++++++++++                                      | 24% ~01s          
  |+++++++++++++                                     | 25% ~01s          
  |++++++++++++++                                    | 26% ~01s          
  |++++++++++++++                                    | 28% ~01s          
  |+++++++++++++++                                   | 29% ~01s          
  |+++++++++++++++                                   | 30% ~01s          
  |++++++++++++++++                                  | 31% ~01s          
  |+++++++++++++++++                                 | 32% ~01s          
  |+++++++++++++++++                                 | 34% ~01s          
  |++++++++++++++++++                                | 35% ~01s          
  |+++++++++++++++++++                               | 36% ~01s          
  |+++++++++++++++++++                               | 38% ~01s          
  |++++++++++++++++++++                              | 39% ~01s          
  |++++++++++++++++++++                              | 40% ~01s          
  |+++++++++++++++++++++                             | 41% ~01s          
  |++++++++++++++++++++++                            | 42% ~01s          
  |++++++++++++++++++++++                            | 44% ~01s          
  |+++++++++++++++++++++++                           | 45% ~01s          
  |++++++++++++++++++++++++                          | 46% ~01s          
  |++++++++++++++++++++++++                          | 48% ~01s          
  |+++++++++++++++++++++++++                         | 49% ~01s          
  |+++++++++++++++++++++++++                         | 50% ~01s          
  |++++++++++++++++++++++++++                        | 51% ~01s          
  |+++++++++++++++++++++++++++                       | 52% ~01s          
  |+++++++++++++++++++++++++++                       | 54% ~01s          
  |++++++++++++++++++++++++++++                      | 55% ~01s          
  |+++++++++++++++++++++++++++++                     | 56% ~01s          
  |+++++++++++++++++++++++++++++                     | 58% ~01s          
  |++++++++++++++++++++++++++++++                    | 59% ~01s          
  |++++++++++++++++++++++++++++++                    | 60% ~01s          
  |+++++++++++++++++++++++++++++++                   | 61% ~01s          
  |++++++++++++++++++++++++++++++++                  | 62% ~01s          
  |++++++++++++++++++++++++++++++++                  | 64% ~01s          
  |+++++++++++++++++++++++++++++++++                 | 65% ~01s          
  |++++++++++++++++++++++++++++++++++                | 66% ~00s          
  |++++++++++++++++++++++++++++++++++                | 68% ~00s          
  |+++++++++++++++++++++++++++++++++++               | 69% ~00s          
  |+++++++++++++++++++++++++++++++++++               | 70% ~00s          
  |++++++++++++++++++++++++++++++++++++              | 71% ~00s          
  |+++++++++++++++++++++++++++++++++++++             | 72% ~00s          
  |+++++++++++++++++++++++++++++++++++++             | 74% ~00s          
  |++++++++++++++++++++++++++++++++++++++            | 75% ~00s          
  |+++++++++++++++++++++++++++++++++++++++           | 76% ~00s          
  |+++++++++++++++++++++++++++++++++++++++           | 78% ~00s          
  |++++++++++++++++++++++++++++++++++++++++          | 79% ~00s          
  |++++++++++++++++++++++++++++++++++++++++          | 80% ~00s          
  |+++++++++++++++++++++++++++++++++++++++++         | 81% ~00s          
  |++++++++++++++++++++++++++++++++++++++++++        | 82% ~00s          
  |++++++++++++++++++++++++++++++++++++++++++        | 84% ~00s          
  |+++++++++++++++++++++++++++++++++++++++++++       | 85% ~00s          
  |++++++++++++++++++++++++++++++++++++++++++++      | 86% ~00s          
  |++++++++++++++++++++++++++++++++++++++++++++      | 88% ~00s          
  |+++++++++++++++++++++++++++++++++++++++++++++     | 89% ~00s          
  |+++++++++++++++++++++++++++++++++++++++++++++     | 90% ~00s          
  |++++++++++++++++++++++++++++++++++++++++++++++    | 91% ~00s          
  |+++++++++++++++++++++++++++++++++++++++++++++++   | 92% ~00s          
  |+++++++++++++++++++++++++++++++++++++++++++++++   | 94% ~00s          
  |++++++++++++++++++++++++++++++++++++++++++++++++  | 95% ~00s          
  |+++++++++++++++++++++++++++++++++++++++++++++++++ | 96% ~00s          
  |+++++++++++++++++++++++++++++++++++++++++++++++++ | 98% ~00s          
  |++++++++++++++++++++++++++++++++++++++++++++++++++| 99% ~00s          
  |++++++++++++++++++++++++++++++++++++++++++++++++++| 100% elapsed=02s  

  |                                                  | 0 % ~calculating  
  |+                                                 | 1 % ~00s          
  |++                                                | 2 % ~00s          
  |++                                                | 4 % ~01s          
  |+++                                               | 5 % ~01s          
  |++++                                              | 6 % ~01s          
  |++++                                              | 8 % ~00s          
  |+++++                                             | 9 % ~01s          
  |+++++                                             | 10% ~01s          
  |++++++                                            | 11% ~01s          
  |+++++++                                           | 12% ~01s          
  |+++++++                                           | 14% ~01s          
  |++++++++                                          | 15% ~01s          
  |+++++++++                                         | 16% ~01s          
  |+++++++++                                         | 18% ~01s          
  |++++++++++                                        | 19% ~01s          
  |++++++++++                                        | 20% ~00s          
  |+++++++++++                                       | 21% ~01s          
  |++++++++++++                                      | 22% ~00s          
  |++++++++++++                                      | 24% ~00s          
  |+++++++++++++                                     | 25% ~00s          
  |++++++++++++++                                    | 26% ~00s          
  |++++++++++++++                                    | 28% ~00s          
  |+++++++++++++++                                   | 29% ~01s          
  |+++++++++++++++                                   | 30% ~01s          
  |++++++++++++++++                                  | 31% ~01s          
  |+++++++++++++++++                                 | 32% ~01s          
  |+++++++++++++++++                                 | 34% ~01s          
  |++++++++++++++++++                                | 35% ~01s          
  |+++++++++++++++++++                               | 36% ~00s          
  |+++++++++++++++++++                               | 38% ~00s          
  |++++++++++++++++++++                              | 39% ~00s          
  |++++++++++++++++++++                              | 40% ~00s          
  |+++++++++++++++++++++                             | 41% ~00s          
  |++++++++++++++++++++++                            | 42% ~00s          
  |++++++++++++++++++++++                            | 44% ~00s          
  |+++++++++++++++++++++++                           | 45% ~00s          
  |++++++++++++++++++++++++                          | 46% ~00s          
  |++++++++++++++++++++++++                          | 48% ~00s          
  |+++++++++++++++++++++++++                         | 49% ~00s          
  |+++++++++++++++++++++++++                         | 50% ~00s          
  |++++++++++++++++++++++++++                        | 51% ~00s          
  |+++++++++++++++++++++++++++                       | 52% ~00s          
  |+++++++++++++++++++++++++++                       | 54% ~00s          
  |++++++++++++++++++++++++++++                      | 55% ~00s          
  |+++++++++++++++++++++++++++++                     | 56% ~00s          
  |+++++++++++++++++++++++++++++                     | 58% ~00s          
  |++++++++++++++++++++++++++++++                    | 59% ~00s          
  |++++++++++++++++++++++++++++++                    | 60% ~00s          
  |+++++++++++++++++++++++++++++++                   | 61% ~00s          
  |++++++++++++++++++++++++++++++++                  | 62% ~00s          
  |++++++++++++++++++++++++++++++++                  | 64% ~00s          
  |+++++++++++++++++++++++++++++++++                 | 65% ~00s          
  |++++++++++++++++++++++++++++++++++                | 66% ~00s          
  |++++++++++++++++++++++++++++++++++                | 68% ~00s          
  |+++++++++++++++++++++++++++++++++++               | 69% ~00s          
  |+++++++++++++++++++++++++++++++++++               | 70% ~00s          
  |++++++++++++++++++++++++++++++++++++              | 71% ~00s          
  |+++++++++++++++++++++++++++++++++++++             | 72% ~00s          
  |+++++++++++++++++++++++++++++++++++++             | 74% ~00s          
  |++++++++++++++++++++++++++++++++++++++            | 75% ~00s          
  |+++++++++++++++++++++++++++++++++++++++           | 76% ~00s          
  |+++++++++++++++++++++++++++++++++++++++           | 78% ~00s          
  |++++++++++++++++++++++++++++++++++++++++          | 79% ~00s          
  |++++++++++++++++++++++++++++++++++++++++          | 80% ~00s          
  |+++++++++++++++++++++++++++++++++++++++++         | 81% ~00s          
  |++++++++++++++++++++++++++++++++++++++++++        | 82% ~00s          
  |++++++++++++++++++++++++++++++++++++++++++        | 84% ~00s          
  |+++++++++++++++++++++++++++++++++++++++++++       | 85% ~00s          
  |++++++++++++++++++++++++++++++++++++++++++++      | 86% ~00s          
  |++++++++++++++++++++++++++++++++++++++++++++      | 88% ~00s          
  |+++++++++++++++++++++++++++++++++++++++++++++     | 89% ~00s          
  |+++++++++++++++++++++++++++++++++++++++++++++     | 90% ~00s          
  |++++++++++++++++++++++++++++++++++++++++++++++    | 91% ~00s          
  |+++++++++++++++++++++++++++++++++++++++++++++++   | 92% ~00s          
  |+++++++++++++++++++++++++++++++++++++++++++++++   | 94% ~00s          
  |++++++++++++++++++++++++++++++++++++++++++++++++  | 95% ~00s          
  |+++++++++++++++++++++++++++++++++++++++++++++++++ | 96% ~00s          
  |+++++++++++++++++++++++++++++++++++++++++++++++++ | 98% ~00s          
  |++++++++++++++++++++++++++++++++++++++++++++++++++| 99% ~00s          
  |++++++++++++++++++++++++++++++++++++++++++++++++++| 100% elapsed=01s  

  |                                                  | 0 % ~calculating  
  |+                                                 | 1 % ~02s          
  |++                                                | 2 % ~02s          
  |++                                                | 4 % ~02s          
  |+++                                               | 5 % ~02s          
  |++++                                              | 6 % ~02s          
  |++++                                              | 8 % ~02s          
  |+++++                                             | 9 % ~02s          
  |+++++                                             | 10% ~02s          
  |++++++                                            | 11% ~02s          
  |+++++++                                           | 12% ~02s          
  |+++++++                                           | 14% ~02s          
  |++++++++                                          | 15% ~01s          
  |+++++++++                                         | 16% ~01s          
  |+++++++++                                         | 18% ~01s          
  |++++++++++                                        | 19% ~01s          
  |++++++++++                                        | 20% ~01s          
  |+++++++++++                                       | 21% ~01s          
  |++++++++++++                                      | 22% ~01s          
  |++++++++++++                                      | 24% ~01s          
  |+++++++++++++                                     | 25% ~01s          
  |++++++++++++++                                    | 26% ~01s          
  |++++++++++++++                                    | 28% ~01s          
  |+++++++++++++++                                   | 29% ~01s          
  |+++++++++++++++                                   | 30% ~01s          
  |++++++++++++++++                                  | 31% ~01s          
  |+++++++++++++++++                                 | 32% ~01s          
  |+++++++++++++++++                                 | 34% ~01s          
  |++++++++++++++++++                                | 35% ~01s          
  |+++++++++++++++++++                               | 36% ~01s          
  |+++++++++++++++++++                               | 38% ~01s          
  |++++++++++++++++++++                              | 39% ~01s          
  |++++++++++++++++++++                              | 40% ~01s          
  |+++++++++++++++++++++                             | 41% ~01s          
  |++++++++++++++++++++++                            | 42% ~01s          
  |++++++++++++++++++++++                            | 44% ~01s          
  |+++++++++++++++++++++++                           | 45% ~01s          
  |++++++++++++++++++++++++                          | 46% ~01s          
  |++++++++++++++++++++++++                          | 48% ~01s          
  |+++++++++++++++++++++++++                         | 49% ~01s          
  |+++++++++++++++++++++++++                         | 50% ~01s          
  |++++++++++++++++++++++++++                        | 51% ~01s          
  |+++++++++++++++++++++++++++                       | 52% ~01s          
  |+++++++++++++++++++++++++++                       | 54% ~01s          
  |++++++++++++++++++++++++++++                      | 55% ~01s          
  |+++++++++++++++++++++++++++++                     | 56% ~01s          
  |+++++++++++++++++++++++++++++                     | 58% ~01s          
  |++++++++++++++++++++++++++++++                    | 59% ~01s          
  |++++++++++++++++++++++++++++++                    | 60% ~01s          
  |+++++++++++++++++++++++++++++++                   | 61% ~01s          
  |++++++++++++++++++++++++++++++++                  | 62% ~01s          
  |++++++++++++++++++++++++++++++++                  | 64% ~01s          
  |+++++++++++++++++++++++++++++++++                 | 65% ~01s          
  |++++++++++++++++++++++++++++++++++                | 66% ~01s          
  |++++++++++++++++++++++++++++++++++                | 68% ~01s          
  |+++++++++++++++++++++++++++++++++++               | 69% ~01s          
  |+++++++++++++++++++++++++++++++++++               | 70% ~01s          
  |++++++++++++++++++++++++++++++++++++              | 71% ~01s          
  |+++++++++++++++++++++++++++++++++++++             | 72% ~00s          
  |+++++++++++++++++++++++++++++++++++++             | 74% ~00s          
  |++++++++++++++++++++++++++++++++++++++            | 75% ~00s          
  |+++++++++++++++++++++++++++++++++++++++           | 76% ~00s          
  |+++++++++++++++++++++++++++++++++++++++           | 78% ~00s          
  |++++++++++++++++++++++++++++++++++++++++          | 79% ~00s          
  |++++++++++++++++++++++++++++++++++++++++          | 80% ~00s          
  |+++++++++++++++++++++++++++++++++++++++++         | 81% ~00s          
  |++++++++++++++++++++++++++++++++++++++++++        | 82% ~00s          
  |++++++++++++++++++++++++++++++++++++++++++        | 84% ~00s          
  |+++++++++++++++++++++++++++++++++++++++++++       | 85% ~00s          
  |++++++++++++++++++++++++++++++++++++++++++++      | 86% ~00s          
  |++++++++++++++++++++++++++++++++++++++++++++      | 88% ~00s          
  |+++++++++++++++++++++++++++++++++++++++++++++     | 89% ~00s          
  |+++++++++++++++++++++++++++++++++++++++++++++     | 90% ~00s          
  |++++++++++++++++++++++++++++++++++++++++++++++    | 91% ~00s          
  |+++++++++++++++++++++++++++++++++++++++++++++++   | 92% ~00s          
  |+++++++++++++++++++++++++++++++++++++++++++++++   | 94% ~00s          
  |++++++++++++++++++++++++++++++++++++++++++++++++  | 95% ~00s          
  |+++++++++++++++++++++++++++++++++++++++++++++++++ | 96% ~00s          
  |+++++++++++++++++++++++++++++++++++++++++++++++++ | 98% ~00s          
  |++++++++++++++++++++++++++++++++++++++++++++++++++| 99% ~00s          
  |++++++++++++++++++++++++++++++++++++++++++++++++++| 100% elapsed=02s  

  |                                                  | 0 % ~calculating  
  |+                                                 | 1 % ~02s          
  |++                                                | 2 % ~01s          
  |++                                                | 4 % ~01s          
  |+++                                               | 5 % ~02s          
  |++++                                              | 6 % ~02s          
  |++++                                              | 8 % ~02s          
  |+++++                                             | 9 % ~01s          
  |+++++                                             | 10% ~01s          
  |++++++                                            | 11% ~01s          
  |+++++++                                           | 12% ~01s          
  |+++++++                                           | 14% ~01s          
  |++++++++                                          | 15% ~01s          
  |+++++++++                                         | 16% ~01s          
  |+++++++++                                         | 18% ~01s          
  |++++++++++                                        | 19% ~01s          
  |++++++++++                                        | 20% ~01s          
  |+++++++++++                                       | 21% ~01s          
  |++++++++++++                                      | 22% ~01s          
  |++++++++++++                                      | 24% ~01s          
  |+++++++++++++                                     | 25% ~01s          
  |++++++++++++++                                    | 26% ~01s          
  |++++++++++++++                                    | 28% ~01s          
  |+++++++++++++++                                   | 29% ~01s          
  |+++++++++++++++                                   | 30% ~01s          
  |++++++++++++++++                                  | 31% ~01s          
  |+++++++++++++++++                                 | 32% ~01s          
  |+++++++++++++++++                                 | 34% ~01s          
  |++++++++++++++++++                                | 35% ~01s          
  |+++++++++++++++++++                               | 36% ~01s          
  |+++++++++++++++++++                               | 38% ~01s          
  |++++++++++++++++++++                              | 39% ~01s          
  |++++++++++++++++++++                              | 40% ~01s          
  |+++++++++++++++++++++                             | 41% ~01s          
  |++++++++++++++++++++++                            | 42% ~01s          
  |++++++++++++++++++++++                            | 44% ~01s          
  |+++++++++++++++++++++++                           | 45% ~01s          
  |++++++++++++++++++++++++                          | 46% ~01s          
  |++++++++++++++++++++++++                          | 48% ~01s          
  |+++++++++++++++++++++++++                         | 49% ~01s          
  |+++++++++++++++++++++++++                         | 50% ~01s          
  |++++++++++++++++++++++++++                        | 51% ~01s          
  |+++++++++++++++++++++++++++                       | 52% ~01s          
  |+++++++++++++++++++++++++++                       | 54% ~01s          
  |++++++++++++++++++++++++++++                      | 55% ~01s          
  |+++++++++++++++++++++++++++++                     | 56% ~01s          
  |+++++++++++++++++++++++++++++                     | 58% ~01s          
  |++++++++++++++++++++++++++++++                    | 59% ~01s          
  |++++++++++++++++++++++++++++++                    | 60% ~01s          
  |+++++++++++++++++++++++++++++++                   | 61% ~01s          
  |++++++++++++++++++++++++++++++++                  | 62% ~01s          
  |++++++++++++++++++++++++++++++++                  | 64% ~01s          
  |+++++++++++++++++++++++++++++++++                 | 65% ~01s          
  |++++++++++++++++++++++++++++++++++                | 66% ~00s          
  |++++++++++++++++++++++++++++++++++                | 68% ~00s          
  |+++++++++++++++++++++++++++++++++++               | 69% ~00s          
  |+++++++++++++++++++++++++++++++++++               | 70% ~00s          
  |++++++++++++++++++++++++++++++++++++              | 71% ~00s          
  |+++++++++++++++++++++++++++++++++++++             | 72% ~00s          
  |+++++++++++++++++++++++++++++++++++++             | 74% ~00s          
  |++++++++++++++++++++++++++++++++++++++            | 75% ~00s          
  |+++++++++++++++++++++++++++++++++++++++           | 76% ~00s          
  |+++++++++++++++++++++++++++++++++++++++           | 78% ~00s          
  |++++++++++++++++++++++++++++++++++++++++          | 79% ~00s          
  |++++++++++++++++++++++++++++++++++++++++          | 80% ~00s          
  |+++++++++++++++++++++++++++++++++++++++++         | 81% ~00s          
  |++++++++++++++++++++++++++++++++++++++++++        | 82% ~00s          
  |++++++++++++++++++++++++++++++++++++++++++        | 84% ~00s          
  |+++++++++++++++++++++++++++++++++++++++++++       | 85% ~00s          
  |++++++++++++++++++++++++++++++++++++++++++++      | 86% ~00s          
  |++++++++++++++++++++++++++++++++++++++++++++      | 88% ~00s          
  |+++++++++++++++++++++++++++++++++++++++++++++     | 89% ~00s          
  |+++++++++++++++++++++++++++++++++++++++++++++     | 90% ~00s          
  |++++++++++++++++++++++++++++++++++++++++++++++    | 91% ~00s          
  |+++++++++++++++++++++++++++++++++++++++++++++++   | 92% ~00s          
  |+++++++++++++++++++++++++++++++++++++++++++++++   | 94% ~00s          
  |++++++++++++++++++++++++++++++++++++++++++++++++  | 95% ~00s          
  |+++++++++++++++++++++++++++++++++++++++++++++++++ | 96% ~00s          
  |+++++++++++++++++++++++++++++++++++++++++++++++++ | 98% ~00s          
  |++++++++++++++++++++++++++++++++++++++++++++++++++| 99% ~00s          
  |++++++++++++++++++++++++++++++++++++++++++++++++++| 100% elapsed=01s  

  |                                                  | 0 % ~calculating  
  |+                                                 | 1 % ~02s          
  |++                                                | 2 % ~02s          
  |++                                                | 4 % ~02s          
  |+++                                               | 5 % ~02s          
  |++++                                              | 6 % ~02s          
  |++++                                              | 8 % ~02s          
  |+++++                                             | 9 % ~02s          
  |+++++                                             | 10% ~02s          
  |++++++                                            | 11% ~02s          
  |+++++++                                           | 12% ~02s          
  |+++++++                                           | 14% ~02s          
  |++++++++                                          | 15% ~02s          
  |+++++++++                                         | 16% ~02s          
  |+++++++++                                         | 18% ~02s          
  |++++++++++                                        | 19% ~02s          
  |++++++++++                                        | 20% ~02s          
  |+++++++++++                                       | 21% ~02s          
  |++++++++++++                                      | 22% ~02s          
  |++++++++++++                                      | 24% ~02s          
  |+++++++++++++                                     | 25% ~02s          
  |++++++++++++++                                    | 26% ~02s          
  |++++++++++++++                                    | 28% ~02s          
  |+++++++++++++++                                   | 29% ~01s          
  |+++++++++++++++                                   | 30% ~01s          
  |++++++++++++++++                                  | 31% ~01s          
  |+++++++++++++++++                                 | 32% ~01s          
  |+++++++++++++++++                                 | 34% ~01s          
  |++++++++++++++++++                                | 35% ~01s          
  |+++++++++++++++++++                               | 36% ~01s          
  |+++++++++++++++++++                               | 38% ~01s          
  |++++++++++++++++++++                              | 39% ~01s          
  |++++++++++++++++++++                              | 40% ~01s          
  |+++++++++++++++++++++                             | 41% ~01s          
  |++++++++++++++++++++++                            | 42% ~01s          
  |++++++++++++++++++++++                            | 44% ~01s          
  |+++++++++++++++++++++++                           | 45% ~01s          
  |++++++++++++++++++++++++                          | 46% ~01s          
  |++++++++++++++++++++++++                          | 48% ~01s          
  |+++++++++++++++++++++++++                         | 49% ~01s          
  |+++++++++++++++++++++++++                         | 50% ~01s          
  |++++++++++++++++++++++++++                        | 51% ~01s          
  |+++++++++++++++++++++++++++                       | 52% ~01s          
  |+++++++++++++++++++++++++++                       | 54% ~01s          
  |++++++++++++++++++++++++++++                      | 55% ~01s          
  |+++++++++++++++++++++++++++++                     | 56% ~01s          
  |+++++++++++++++++++++++++++++                     | 58% ~01s          
  |++++++++++++++++++++++++++++++                    | 59% ~01s          
  |++++++++++++++++++++++++++++++                    | 60% ~01s          
  |+++++++++++++++++++++++++++++++                   | 61% ~01s          
  |++++++++++++++++++++++++++++++++                  | 62% ~01s          
  |++++++++++++++++++++++++++++++++                  | 64% ~01s          
  |+++++++++++++++++++++++++++++++++                 | 65% ~01s          
  |++++++++++++++++++++++++++++++++++                | 66% ~01s          
  |++++++++++++++++++++++++++++++++++                | 68% ~01s          
  |+++++++++++++++++++++++++++++++++++               | 69% ~01s          
  |+++++++++++++++++++++++++++++++++++               | 70% ~01s          
  |++++++++++++++++++++++++++++++++++++              | 71% ~01s          
  |+++++++++++++++++++++++++++++++++++++             | 72% ~01s          
  |+++++++++++++++++++++++++++++++++++++             | 74% ~01s          
  |++++++++++++++++++++++++++++++++++++++            | 75% ~00s          
  |+++++++++++++++++++++++++++++++++++++++           | 76% ~00s          
  |+++++++++++++++++++++++++++++++++++++++           | 78% ~00s          
  |++++++++++++++++++++++++++++++++++++++++          | 79% ~00s          
  |++++++++++++++++++++++++++++++++++++++++          | 80% ~00s          
  |+++++++++++++++++++++++++++++++++++++++++         | 81% ~00s          
  |++++++++++++++++++++++++++++++++++++++++++        | 82% ~00s          
  |++++++++++++++++++++++++++++++++++++++++++        | 84% ~00s          
  |+++++++++++++++++++++++++++++++++++++++++++       | 85% ~00s          
  |++++++++++++++++++++++++++++++++++++++++++++      | 86% ~00s          
  |++++++++++++++++++++++++++++++++++++++++++++      | 88% ~00s          
  |+++++++++++++++++++++++++++++++++++++++++++++     | 89% ~00s          
  |+++++++++++++++++++++++++++++++++++++++++++++     | 90% ~00s          
  |++++++++++++++++++++++++++++++++++++++++++++++    | 91% ~00s          
  |+++++++++++++++++++++++++++++++++++++++++++++++   | 92% ~00s          
  |+++++++++++++++++++++++++++++++++++++++++++++++   | 94% ~00s          
  |++++++++++++++++++++++++++++++++++++++++++++++++  | 95% ~00s          
  |+++++++++++++++++++++++++++++++++++++++++++++++++ | 96% ~00s          
  |+++++++++++++++++++++++++++++++++++++++++++++++++ | 98% ~00s          
  |++++++++++++++++++++++++++++++++++++++++++++++++++| 99% ~00s          
  |++++++++++++++++++++++++++++++++++++++++++++++++++| 100% elapsed=02s  

  |                                                  | 0 % ~calculating  
  |+                                                 | 1 % ~02s          
  |++                                                | 2 % ~02s          
  |++                                                | 4 % ~03s          
  |+++                                               | 5 % ~03s          
  |++++                                              | 6 % ~03s          
  |++++                                              | 8 % ~03s          
  |+++++                                             | 9 % ~03s          
  |+++++                                             | 10% ~03s          
  |++++++                                            | 11% ~02s          
  |+++++++                                           | 12% ~02s          
  |+++++++                                           | 14% ~02s          
  |++++++++                                          | 15% ~02s          
  |+++++++++                                         | 16% ~02s          
  |+++++++++                                         | 18% ~02s          
  |++++++++++                                        | 19% ~02s          
  |++++++++++                                        | 20% ~02s          
  |+++++++++++                                       | 21% ~02s          
  |++++++++++++                                      | 22% ~02s          
  |++++++++++++                                      | 24% ~02s          
  |+++++++++++++                                     | 25% ~02s          
  |++++++++++++++                                    | 26% ~02s          
  |++++++++++++++                                    | 28% ~02s          
  |+++++++++++++++                                   | 29% ~02s          
  |+++++++++++++++                                   | 30% ~02s          
  |++++++++++++++++                                  | 31% ~02s          
  |+++++++++++++++++                                 | 32% ~02s          
  |+++++++++++++++++                                 | 34% ~02s          
  |++++++++++++++++++                                | 35% ~02s          
  |+++++++++++++++++++                               | 36% ~02s          
  |+++++++++++++++++++                               | 38% ~02s          
  |++++++++++++++++++++                              | 39% ~02s          
  |++++++++++++++++++++                              | 40% ~02s          
  |+++++++++++++++++++++                             | 41% ~02s          
  |++++++++++++++++++++++                            | 42% ~02s          
  |++++++++++++++++++++++                            | 44% ~02s          
  |+++++++++++++++++++++++                           | 45% ~01s          
  |++++++++++++++++++++++++                          | 46% ~01s          
  |++++++++++++++++++++++++                          | 48% ~01s          
  |+++++++++++++++++++++++++                         | 49% ~01s          
  |+++++++++++++++++++++++++                         | 50% ~01s          
  |++++++++++++++++++++++++++                        | 51% ~01s          
  |+++++++++++++++++++++++++++                       | 52% ~01s          
  |+++++++++++++++++++++++++++                       | 54% ~01s          
  |++++++++++++++++++++++++++++                      | 55% ~01s          
  |+++++++++++++++++++++++++++++                     | 56% ~01s          
  |+++++++++++++++++++++++++++++                     | 58% ~01s          
  |++++++++++++++++++++++++++++++                    | 59% ~01s          
  |++++++++++++++++++++++++++++++                    | 60% ~01s          
  |+++++++++++++++++++++++++++++++                   | 61% ~01s          
  |++++++++++++++++++++++++++++++++                  | 62% ~01s          
  |++++++++++++++++++++++++++++++++                  | 64% ~01s          
  |+++++++++++++++++++++++++++++++++                 | 65% ~01s          
  |++++++++++++++++++++++++++++++++++                | 66% ~01s          
  |++++++++++++++++++++++++++++++++++                | 68% ~01s          
  |+++++++++++++++++++++++++++++++++++               | 69% ~01s          
  |+++++++++++++++++++++++++++++++++++               | 70% ~01s          
  |++++++++++++++++++++++++++++++++++++              | 71% ~01s          
  |+++++++++++++++++++++++++++++++++++++             | 72% ~01s          
  |+++++++++++++++++++++++++++++++++++++             | 74% ~01s          
  |++++++++++++++++++++++++++++++++++++++            | 75% ~01s          
  |+++++++++++++++++++++++++++++++++++++++           | 76% ~01s          
  |+++++++++++++++++++++++++++++++++++++++           | 78% ~01s          
  |++++++++++++++++++++++++++++++++++++++++          | 79% ~01s          
  |++++++++++++++++++++++++++++++++++++++++          | 80% ~01s          
  |+++++++++++++++++++++++++++++++++++++++++         | 81% ~01s          
  |++++++++++++++++++++++++++++++++++++++++++        | 82% ~00s          
  |++++++++++++++++++++++++++++++++++++++++++        | 84% ~00s          
  |+++++++++++++++++++++++++++++++++++++++++++       | 85% ~00s          
  |++++++++++++++++++++++++++++++++++++++++++++      | 86% ~00s          
  |++++++++++++++++++++++++++++++++++++++++++++      | 88% ~00s          
  |+++++++++++++++++++++++++++++++++++++++++++++     | 89% ~00s          
  |+++++++++++++++++++++++++++++++++++++++++++++     | 90% ~00s          
  |++++++++++++++++++++++++++++++++++++++++++++++    | 91% ~00s          
  |+++++++++++++++++++++++++++++++++++++++++++++++   | 92% ~00s          
  |+++++++++++++++++++++++++++++++++++++++++++++++   | 94% ~00s          
  |++++++++++++++++++++++++++++++++++++++++++++++++  | 95% ~00s          
  |+++++++++++++++++++++++++++++++++++++++++++++++++ | 96% ~00s          
  |+++++++++++++++++++++++++++++++++++++++++++++++++ | 98% ~00s          
  |++++++++++++++++++++++++++++++++++++++++++++++++++| 99% ~00s          
  |++++++++++++++++++++++++++++++++++++++++++++++++++| 100% elapsed=03s
```


```
write.csv(df, "epithelial-severity-de-negative-targets.csv")
```


```
goi <- read.table("positive-candidates.txt")$V1
#cnt = 0

data$Domain_disease_patient_type <- paste(data$Domain, data$Disease, data$PatientType2, sep='_')
data$Domain_disease_patient_type <- factor(data$Domain_disease_patient_type, 
                                          levels = c("Epithelial_control_mild", "Epithelial_control_severe",
                                                     "Epithelial_COVID19_mild", "Epithelial_COVID19_severe"
                                                     ))
Idents(data) <- 'Domain_disease_patient_type'

    my_comparisons <- list( c("Epithelial_control_mild", "Epithelial_control_severe"), 
                        c("Epithelial_control_severe", "Epithelial_COVID19_mild"), 
                        c("Epithelial_COVID19_mild", "Epithelial_COVID19_severe"),
                        c("Epithelial_control_mild", "Epithelial_COVID19_mild"), 
                        c("Epithelial_control_severe", "Epithelial_COVID19_severe"),
                        c("Epithelial_control_mild", "Epithelial_COVID19_severe"))

df <- data.frame(nrows = goi)
rownames(df) <- goi

for (cmp in my_comparisons) {
  df[[paste0(cmp[1], '_vs_', cmp[2], '_p')]] <- NA
  df[[paste0(cmp[1], '_vs_', cmp[2], '_p_adj')]] <- NA
  df[[paste0(cmp[1], '_vs_', cmp[2], '_logfc')]] <- NA
  
  mutual_features <- intersect(goi, rownames(data))
  
  deg <- FindMarkers(data, cmp[1], cmp[2], features = mutual_features, slot = "data",
                        min.cells.group = 0, 
                        min.cells.feature = 0,
                        min.pct = -1,
                        logfc.threshold = -1,
                        only.pos = FALSE)
  
  df[mutual_features, paste0(cmp[1], '_vs_', cmp[2], '_p')] <- deg[mutual_features, 'p_val']
  df[mutual_features, paste0(cmp[1], '_vs_', cmp[2], '_p_adj')] <- deg[mutual_features, 'p_val_adj']
  df[mutual_features, paste0(cmp[1], '_vs_', cmp[2], '_logfc')] <- deg[mutual_features, 'avg_log2FC']
}
```


```
  |                                                  | 0 % ~calculating  
  |+                                                 | 2 % ~02s          
  |++                                                | 3 % ~02s          
  |+++                                               | 5 % ~01s          
  |++++                                              | 6 % ~01s          
  |++++                                              | 8 % ~01s          
  |+++++                                             | 10% ~02s          
  |++++++                                            | 11% ~01s          
  |+++++++                                           | 13% ~01s          
  |++++++++                                          | 14% ~01s          
  |++++++++                                          | 16% ~01s          
  |+++++++++                                         | 17% ~01s          
  |++++++++++                                        | 19% ~01s          
  |+++++++++++                                       | 21% ~01s          
  |++++++++++++                                      | 22% ~01s          
  |++++++++++++                                      | 24% ~01s          
  |+++++++++++++                                     | 25% ~01s          
  |++++++++++++++                                    | 27% ~01s          
  |+++++++++++++++                                   | 29% ~01s          
  |++++++++++++++++                                  | 30% ~01s          
  |++++++++++++++++                                  | 32% ~01s          
  |+++++++++++++++++                                 | 33% ~01s          
  |++++++++++++++++++                                | 35% ~01s          
  |+++++++++++++++++++                               | 37% ~01s          
  |++++++++++++++++++++                              | 38% ~01s          
  |++++++++++++++++++++                              | 40% ~01s          
  |+++++++++++++++++++++                             | 41% ~01s          
  |++++++++++++++++++++++                            | 43% ~01s          
  |+++++++++++++++++++++++                           | 44% ~01s          
  |++++++++++++++++++++++++                          | 46% ~01s          
  |++++++++++++++++++++++++                          | 48% ~01s          
  |+++++++++++++++++++++++++                         | 49% ~01s          
  |++++++++++++++++++++++++++                        | 51% ~01s          
  |+++++++++++++++++++++++++++                       | 52% ~01s          
  |+++++++++++++++++++++++++++                       | 54% ~01s          
  |++++++++++++++++++++++++++++                      | 56% ~01s          
  |+++++++++++++++++++++++++++++                     | 57% ~01s          
  |++++++++++++++++++++++++++++++                    | 59% ~01s          
  |+++++++++++++++++++++++++++++++                   | 60% ~01s          
  |+++++++++++++++++++++++++++++++                   | 62% ~01s          
  |++++++++++++++++++++++++++++++++                  | 63% ~01s          
  |+++++++++++++++++++++++++++++++++                 | 65% ~01s          
  |++++++++++++++++++++++++++++++++++                | 67% ~00s          
  |+++++++++++++++++++++++++++++++++++               | 68% ~00s          
  |+++++++++++++++++++++++++++++++++++               | 70% ~00s          
  |++++++++++++++++++++++++++++++++++++              | 71% ~00s          
  |+++++++++++++++++++++++++++++++++++++             | 73% ~00s          
  |++++++++++++++++++++++++++++++++++++++            | 75% ~00s          
  |+++++++++++++++++++++++++++++++++++++++           | 76% ~00s          
  |+++++++++++++++++++++++++++++++++++++++           | 78% ~00s          
  |++++++++++++++++++++++++++++++++++++++++          | 79% ~00s          
  |+++++++++++++++++++++++++++++++++++++++++         | 81% ~00s          
  |++++++++++++++++++++++++++++++++++++++++++        | 83% ~00s          
  |+++++++++++++++++++++++++++++++++++++++++++       | 84% ~00s          
  |+++++++++++++++++++++++++++++++++++++++++++       | 86% ~00s          
  |++++++++++++++++++++++++++++++++++++++++++++      | 87% ~00s          
  |+++++++++++++++++++++++++++++++++++++++++++++     | 89% ~00s          
  |++++++++++++++++++++++++++++++++++++++++++++++    | 90% ~00s          
  |+++++++++++++++++++++++++++++++++++++++++++++++   | 92% ~00s          
  |+++++++++++++++++++++++++++++++++++++++++++++++   | 94% ~00s          
  |++++++++++++++++++++++++++++++++++++++++++++++++  | 95% ~00s          
  |+++++++++++++++++++++++++++++++++++++++++++++++++ | 97% ~00s          
  |++++++++++++++++++++++++++++++++++++++++++++++++++| 98% ~00s          
  |++++++++++++++++++++++++++++++++++++++++++++++++++| 100% elapsed=01s  

  |                                                  | 0 % ~calculating  
  |+                                                 | 2 % ~00s          
  |++                                                | 3 % ~01s          
  |+++                                               | 5 % ~00s          
  |++++                                              | 6 % ~00s          
  |++++                                              | 8 % ~01s          
  |+++++                                             | 10% ~00s          
  |++++++                                            | 11% ~00s          
  |+++++++                                           | 13% ~00s          
  |++++++++                                          | 14% ~00s          
  |++++++++                                          | 16% ~00s          
  |+++++++++                                         | 17% ~00s          
  |++++++++++                                        | 19% ~00s          
  |+++++++++++                                       | 21% ~00s          
  |++++++++++++                                      | 22% ~00s          
  |++++++++++++                                      | 24% ~00s          
  |+++++++++++++                                     | 25% ~00s          
  |++++++++++++++                                    | 27% ~00s          
  |+++++++++++++++                                   | 29% ~00s          
  |++++++++++++++++                                  | 30% ~00s          
  |++++++++++++++++                                  | 32% ~00s          
  |+++++++++++++++++                                 | 33% ~00s          
  |++++++++++++++++++                                | 35% ~00s          
  |+++++++++++++++++++                               | 37% ~00s          
  |++++++++++++++++++++                              | 38% ~00s          
  |++++++++++++++++++++                              | 40% ~00s          
  |+++++++++++++++++++++                             | 41% ~00s          
  |++++++++++++++++++++++                            | 43% ~00s          
  |+++++++++++++++++++++++                           | 44% ~00s          
  |++++++++++++++++++++++++                          | 46% ~00s          
  |++++++++++++++++++++++++                          | 48% ~00s          
  |+++++++++++++++++++++++++                         | 49% ~00s          
  |++++++++++++++++++++++++++                        | 51% ~00s          
  |+++++++++++++++++++++++++++                       | 52% ~00s          
  |+++++++++++++++++++++++++++                       | 54% ~00s          
  |++++++++++++++++++++++++++++                      | 56% ~00s          
  |+++++++++++++++++++++++++++++                     | 57% ~00s          
  |++++++++++++++++++++++++++++++                    | 59% ~00s          
  |+++++++++++++++++++++++++++++++                   | 60% ~00s          
  |+++++++++++++++++++++++++++++++                   | 62% ~00s          
  |++++++++++++++++++++++++++++++++                  | 63% ~00s          
  |+++++++++++++++++++++++++++++++++                 | 65% ~00s          
  |++++++++++++++++++++++++++++++++++                | 67% ~00s          
  |+++++++++++++++++++++++++++++++++++               | 68% ~00s          
  |+++++++++++++++++++++++++++++++++++               | 70% ~00s          
  |++++++++++++++++++++++++++++++++++++              | 71% ~00s          
  |+++++++++++++++++++++++++++++++++++++             | 73% ~00s          
  |++++++++++++++++++++++++++++++++++++++            | 75% ~00s          
  |+++++++++++++++++++++++++++++++++++++++           | 76% ~00s          
  |+++++++++++++++++++++++++++++++++++++++           | 78% ~00s          
  |++++++++++++++++++++++++++++++++++++++++          | 79% ~00s          
  |+++++++++++++++++++++++++++++++++++++++++         | 81% ~00s          
  |++++++++++++++++++++++++++++++++++++++++++        | 83% ~00s          
  |+++++++++++++++++++++++++++++++++++++++++++       | 84% ~00s          
  |+++++++++++++++++++++++++++++++++++++++++++       | 86% ~00s          
  |++++++++++++++++++++++++++++++++++++++++++++      | 87% ~00s          
  |+++++++++++++++++++++++++++++++++++++++++++++     | 89% ~00s          
  |++++++++++++++++++++++++++++++++++++++++++++++    | 90% ~00s          
  |+++++++++++++++++++++++++++++++++++++++++++++++   | 92% ~00s          
  |+++++++++++++++++++++++++++++++++++++++++++++++   | 94% ~00s          
  |++++++++++++++++++++++++++++++++++++++++++++++++  | 95% ~00s          
  |+++++++++++++++++++++++++++++++++++++++++++++++++ | 97% ~00s          
  |++++++++++++++++++++++++++++++++++++++++++++++++++| 98% ~00s          
  |++++++++++++++++++++++++++++++++++++++++++++++++++| 100% elapsed=01s  

  |                                                  | 0 % ~calculating  
  |+                                                 | 2 % ~01s          
  |++                                                | 3 % ~02s          
  |+++                                               | 5 % ~01s          
  |++++                                              | 6 % ~01s          
  |++++                                              | 8 % ~01s          
  |+++++                                             | 10% ~01s          
  |++++++                                            | 11% ~01s          
  |+++++++                                           | 13% ~01s          
  |++++++++                                          | 14% ~01s          
  |++++++++                                          | 16% ~01s          
  |+++++++++                                         | 17% ~01s          
  |++++++++++                                        | 19% ~01s          
  |+++++++++++                                       | 21% ~01s          
  |++++++++++++                                      | 22% ~01s          
  |++++++++++++                                      | 24% ~01s          
  |+++++++++++++                                     | 25% ~01s          
  |++++++++++++++                                    | 27% ~01s          
  |+++++++++++++++                                   | 29% ~01s          
  |++++++++++++++++                                  | 30% ~01s          
  |++++++++++++++++                                  | 32% ~01s          
  |+++++++++++++++++                                 | 33% ~01s          
  |++++++++++++++++++                                | 35% ~01s          
  |+++++++++++++++++++                               | 37% ~01s          
  |++++++++++++++++++++                              | 38% ~01s          
  |++++++++++++++++++++                              | 40% ~01s          
  |+++++++++++++++++++++                             | 41% ~01s          
  |++++++++++++++++++++++                            | 43% ~01s          
  |+++++++++++++++++++++++                           | 44% ~01s          
  |++++++++++++++++++++++++                          | 46% ~01s          
  |++++++++++++++++++++++++                          | 48% ~01s          
  |+++++++++++++++++++++++++                         | 49% ~01s          
  |++++++++++++++++++++++++++                        | 51% ~01s          
  |+++++++++++++++++++++++++++                       | 52% ~01s          
  |+++++++++++++++++++++++++++                       | 54% ~01s          
  |++++++++++++++++++++++++++++                      | 56% ~01s          
  |+++++++++++++++++++++++++++++                     | 57% ~01s          
  |++++++++++++++++++++++++++++++                    | 59% ~01s          
  |+++++++++++++++++++++++++++++++                   | 60% ~01s          
  |+++++++++++++++++++++++++++++++                   | 62% ~01s          
  |++++++++++++++++++++++++++++++++                  | 63% ~01s          
  |+++++++++++++++++++++++++++++++++                 | 65% ~01s          
  |++++++++++++++++++++++++++++++++++                | 67% ~01s          
  |+++++++++++++++++++++++++++++++++++               | 68% ~01s          
  |+++++++++++++++++++++++++++++++++++               | 70% ~00s          
  |++++++++++++++++++++++++++++++++++++              | 71% ~00s          
  |+++++++++++++++++++++++++++++++++++++             | 73% ~00s          
  |++++++++++++++++++++++++++++++++++++++            | 75% ~00s          
  |+++++++++++++++++++++++++++++++++++++++           | 76% ~00s          
  |+++++++++++++++++++++++++++++++++++++++           | 78% ~00s          
  |++++++++++++++++++++++++++++++++++++++++          | 79% ~00s          
  |+++++++++++++++++++++++++++++++++++++++++         | 81% ~00s          
  |++++++++++++++++++++++++++++++++++++++++++        | 83% ~00s          
  |+++++++++++++++++++++++++++++++++++++++++++       | 84% ~00s          
  |+++++++++++++++++++++++++++++++++++++++++++       | 86% ~00s          
  |++++++++++++++++++++++++++++++++++++++++++++      | 87% ~00s          
  |+++++++++++++++++++++++++++++++++++++++++++++     | 89% ~00s          
  |++++++++++++++++++++++++++++++++++++++++++++++    | 90% ~00s          
  |+++++++++++++++++++++++++++++++++++++++++++++++   | 92% ~00s          
  |+++++++++++++++++++++++++++++++++++++++++++++++   | 94% ~00s          
  |++++++++++++++++++++++++++++++++++++++++++++++++  | 95% ~00s          
  |+++++++++++++++++++++++++++++++++++++++++++++++++ | 97% ~00s          
  |++++++++++++++++++++++++++++++++++++++++++++++++++| 98% ~00s          
  |++++++++++++++++++++++++++++++++++++++++++++++++++| 100% elapsed=02s  

  |                                                  | 0 % ~calculating  
  |+                                                 | 2 % ~01s          
  |++                                                | 3 % ~01s          
  |+++                                               | 5 % ~01s          
  |++++                                              | 6 % ~01s          
  |++++                                              | 8 % ~01s          
  |+++++                                             | 10% ~01s          
  |++++++                                            | 11% ~01s          
  |+++++++                                           | 13% ~01s          
  |++++++++                                          | 14% ~01s          
  |++++++++                                          | 16% ~01s          
  |+++++++++                                         | 17% ~01s          
  |++++++++++                                        | 19% ~01s          
  |+++++++++++                                       | 21% ~01s          
  |++++++++++++                                      | 22% ~01s          
  |++++++++++++                                      | 24% ~01s          
  |+++++++++++++                                     | 25% ~01s          
  |++++++++++++++                                    | 27% ~01s          
  |+++++++++++++++                                   | 29% ~01s          
  |++++++++++++++++                                  | 30% ~01s          
  |++++++++++++++++                                  | 32% ~01s          
  |+++++++++++++++++                                 | 33% ~01s          
  |++++++++++++++++++                                | 35% ~01s          
  |+++++++++++++++++++                               | 37% ~01s          
  |++++++++++++++++++++                              | 38% ~01s          
  |++++++++++++++++++++                              | 40% ~01s          
  |+++++++++++++++++++++                             | 41% ~01s          
  |++++++++++++++++++++++                            | 43% ~01s          
  |+++++++++++++++++++++++                           | 44% ~01s          
  |++++++++++++++++++++++++                          | 46% ~01s          
  |++++++++++++++++++++++++                          | 48% ~01s          
  |+++++++++++++++++++++++++                         | 49% ~01s          
  |++++++++++++++++++++++++++                        | 51% ~01s          
  |+++++++++++++++++++++++++++                       | 52% ~01s          
  |+++++++++++++++++++++++++++                       | 54% ~01s          
  |++++++++++++++++++++++++++++                      | 56% ~01s          
  |+++++++++++++++++++++++++++++                     | 57% ~01s          
  |++++++++++++++++++++++++++++++                    | 59% ~01s          
  |+++++++++++++++++++++++++++++++                   | 60% ~00s          
  |+++++++++++++++++++++++++++++++                   | 62% ~00s          
  |++++++++++++++++++++++++++++++++                  | 63% ~00s          
  |+++++++++++++++++++++++++++++++++                 | 65% ~00s          
  |++++++++++++++++++++++++++++++++++                | 67% ~00s          
  |+++++++++++++++++++++++++++++++++++               | 68% ~00s          
  |+++++++++++++++++++++++++++++++++++               | 70% ~00s          
  |++++++++++++++++++++++++++++++++++++              | 71% ~00s          
  |+++++++++++++++++++++++++++++++++++++             | 73% ~00s          
  |++++++++++++++++++++++++++++++++++++++            | 75% ~00s          
  |+++++++++++++++++++++++++++++++++++++++           | 76% ~00s          
  |+++++++++++++++++++++++++++++++++++++++           | 78% ~00s          
  |++++++++++++++++++++++++++++++++++++++++          | 79% ~00s          
  |+++++++++++++++++++++++++++++++++++++++++         | 81% ~00s          
  |++++++++++++++++++++++++++++++++++++++++++        | 83% ~00s          
  |+++++++++++++++++++++++++++++++++++++++++++       | 84% ~00s          
  |+++++++++++++++++++++++++++++++++++++++++++       | 86% ~00s          
  |++++++++++++++++++++++++++++++++++++++++++++      | 87% ~00s          
  |+++++++++++++++++++++++++++++++++++++++++++++     | 89% ~00s          
  |++++++++++++++++++++++++++++++++++++++++++++++    | 90% ~00s          
  |+++++++++++++++++++++++++++++++++++++++++++++++   | 92% ~00s          
  |+++++++++++++++++++++++++++++++++++++++++++++++   | 94% ~00s          
  |++++++++++++++++++++++++++++++++++++++++++++++++  | 95% ~00s          
  |+++++++++++++++++++++++++++++++++++++++++++++++++ | 97% ~00s          
  |++++++++++++++++++++++++++++++++++++++++++++++++++| 98% ~00s          
  |++++++++++++++++++++++++++++++++++++++++++++++++++| 100% elapsed=01s  

  |                                                  | 0 % ~calculating  
  |+                                                 | 2 % ~01s          
  |++                                                | 3 % ~01s          
  |+++                                               | 5 % ~01s          
  |++++                                              | 6 % ~01s          
  |++++                                              | 8 % ~01s          
  |+++++                                             | 10% ~01s          
  |++++++                                            | 11% ~01s          
  |+++++++                                           | 13% ~01s          
  |++++++++                                          | 14% ~01s          
  |++++++++                                          | 16% ~01s          
  |+++++++++                                         | 17% ~01s          
  |++++++++++                                        | 19% ~01s          
  |+++++++++++                                       | 21% ~01s          
  |++++++++++++                                      | 22% ~01s          
  |++++++++++++                                      | 24% ~01s          
  |+++++++++++++                                     | 25% ~01s          
  |++++++++++++++                                    | 27% ~01s          
  |+++++++++++++++                                   | 29% ~01s          
  |++++++++++++++++                                  | 30% ~01s          
  |++++++++++++++++                                  | 32% ~01s          
  |+++++++++++++++++                                 | 33% ~01s          
  |++++++++++++++++++                                | 35% ~01s          
  |+++++++++++++++++++                               | 37% ~01s          
  |++++++++++++++++++++                              | 38% ~01s          
  |++++++++++++++++++++                              | 40% ~01s          
  |+++++++++++++++++++++                             | 41% ~01s          
  |++++++++++++++++++++++                            | 43% ~01s          
  |+++++++++++++++++++++++                           | 44% ~01s          
  |++++++++++++++++++++++++                          | 46% ~01s          
  |++++++++++++++++++++++++                          | 48% ~01s          
  |+++++++++++++++++++++++++                         | 49% ~01s          
  |++++++++++++++++++++++++++                        | 51% ~01s          
  |+++++++++++++++++++++++++++                       | 52% ~01s          
  |+++++++++++++++++++++++++++                       | 54% ~01s          
  |++++++++++++++++++++++++++++                      | 56% ~01s          
  |+++++++++++++++++++++++++++++                     | 57% ~01s          
  |++++++++++++++++++++++++++++++                    | 59% ~01s          
  |+++++++++++++++++++++++++++++++                   | 60% ~01s          
  |+++++++++++++++++++++++++++++++                   | 62% ~01s          
  |++++++++++++++++++++++++++++++++                  | 63% ~01s          
  |+++++++++++++++++++++++++++++++++                 | 65% ~01s          
  |++++++++++++++++++++++++++++++++++                | 67% ~01s          
  |+++++++++++++++++++++++++++++++++++               | 68% ~01s          
  |+++++++++++++++++++++++++++++++++++               | 70% ~00s          
  |++++++++++++++++++++++++++++++++++++              | 71% ~00s          
  |+++++++++++++++++++++++++++++++++++++             | 73% ~00s          
  |++++++++++++++++++++++++++++++++++++++            | 75% ~00s          
  |+++++++++++++++++++++++++++++++++++++++           | 76% ~00s          
  |+++++++++++++++++++++++++++++++++++++++           | 78% ~00s          
  |++++++++++++++++++++++++++++++++++++++++          | 79% ~00s          
  |+++++++++++++++++++++++++++++++++++++++++         | 81% ~00s          
  |++++++++++++++++++++++++++++++++++++++++++        | 83% ~00s          
  |+++++++++++++++++++++++++++++++++++++++++++       | 84% ~00s          
  |+++++++++++++++++++++++++++++++++++++++++++       | 86% ~00s          
  |++++++++++++++++++++++++++++++++++++++++++++      | 87% ~00s          
  |+++++++++++++++++++++++++++++++++++++++++++++     | 89% ~00s          
  |++++++++++++++++++++++++++++++++++++++++++++++    | 90% ~00s          
  |+++++++++++++++++++++++++++++++++++++++++++++++   | 92% ~00s          
  |+++++++++++++++++++++++++++++++++++++++++++++++   | 94% ~00s          
  |++++++++++++++++++++++++++++++++++++++++++++++++  | 95% ~00s          
  |+++++++++++++++++++++++++++++++++++++++++++++++++ | 97% ~00s          
  |++++++++++++++++++++++++++++++++++++++++++++++++++| 98% ~00s          
  |++++++++++++++++++++++++++++++++++++++++++++++++++| 100% elapsed=02s  

  |                                                  | 0 % ~calculating  
  |+                                                 | 2 % ~02s          
  |++                                                | 3 % ~02s          
  |+++                                               | 5 % ~02s          
  |++++                                              | 6 % ~02s          
  |++++                                              | 8 % ~02s          
  |+++++                                             | 10% ~02s          
  |++++++                                            | 11% ~02s          
  |+++++++                                           | 13% ~02s          
  |++++++++                                          | 14% ~02s          
  |++++++++                                          | 16% ~02s          
  |+++++++++                                         | 17% ~02s          
  |++++++++++                                        | 19% ~02s          
  |+++++++++++                                       | 21% ~02s          
  |++++++++++++                                      | 22% ~02s          
  |++++++++++++                                      | 24% ~02s          
  |+++++++++++++                                     | 25% ~02s          
  |++++++++++++++                                    | 27% ~02s          
  |+++++++++++++++                                   | 29% ~02s          
  |++++++++++++++++                                  | 30% ~02s          
  |++++++++++++++++                                  | 32% ~02s          
  |+++++++++++++++++                                 | 33% ~01s          
  |++++++++++++++++++                                | 35% ~01s          
  |+++++++++++++++++++                               | 37% ~01s          
  |++++++++++++++++++++                              | 38% ~01s          
  |++++++++++++++++++++                              | 40% ~01s          
  |+++++++++++++++++++++                             | 41% ~01s          
  |++++++++++++++++++++++                            | 43% ~01s          
  |+++++++++++++++++++++++                           | 44% ~01s          
  |++++++++++++++++++++++++                          | 46% ~01s          
  |++++++++++++++++++++++++                          | 48% ~01s          
  |+++++++++++++++++++++++++                         | 49% ~01s          
  |++++++++++++++++++++++++++                        | 51% ~01s          
  |+++++++++++++++++++++++++++                       | 52% ~01s          
  |+++++++++++++++++++++++++++                       | 54% ~01s          
  |++++++++++++++++++++++++++++                      | 56% ~01s          
  |+++++++++++++++++++++++++++++                     | 57% ~01s          
  |++++++++++++++++++++++++++++++                    | 59% ~01s          
  |+++++++++++++++++++++++++++++++                   | 60% ~01s          
  |+++++++++++++++++++++++++++++++                   | 62% ~01s          
  |++++++++++++++++++++++++++++++++                  | 63% ~01s          
  |+++++++++++++++++++++++++++++++++                 | 65% ~01s          
  |++++++++++++++++++++++++++++++++++                | 67% ~01s          
  |+++++++++++++++++++++++++++++++++++               | 68% ~01s          
  |+++++++++++++++++++++++++++++++++++               | 70% ~01s          
  |++++++++++++++++++++++++++++++++++++              | 71% ~01s          
  |+++++++++++++++++++++++++++++++++++++             | 73% ~01s          
  |++++++++++++++++++++++++++++++++++++++            | 75% ~01s          
  |+++++++++++++++++++++++++++++++++++++++           | 76% ~01s          
  |+++++++++++++++++++++++++++++++++++++++           | 78% ~01s          
  |++++++++++++++++++++++++++++++++++++++++          | 79% ~00s          
  |+++++++++++++++++++++++++++++++++++++++++         | 81% ~00s          
  |++++++++++++++++++++++++++++++++++++++++++        | 83% ~00s          
  |+++++++++++++++++++++++++++++++++++++++++++       | 84% ~00s          
  |+++++++++++++++++++++++++++++++++++++++++++       | 86% ~00s          
  |++++++++++++++++++++++++++++++++++++++++++++      | 87% ~00s          
  |+++++++++++++++++++++++++++++++++++++++++++++     | 89% ~00s          
  |++++++++++++++++++++++++++++++++++++++++++++++    | 90% ~00s          
  |+++++++++++++++++++++++++++++++++++++++++++++++   | 92% ~00s          
  |+++++++++++++++++++++++++++++++++++++++++++++++   | 94% ~00s          
  |++++++++++++++++++++++++++++++++++++++++++++++++  | 95% ~00s          
  |+++++++++++++++++++++++++++++++++++++++++++++++++ | 97% ~00s          
  |++++++++++++++++++++++++++++++++++++++++++++++++++| 98% ~00s          
  |++++++++++++++++++++++++++++++++++++++++++++++++++| 100% elapsed=02s
```


```
write.csv(df, "epithelial-severity-de-positive-targets.csv")
```

LS0tDQp0aXRsZTogIkZpZ3VyZSAyZCBzb3VyY2UgZGF0YSBnZW5lcmF0aW9uIg0Kb3V0cHV0OiBodG1sX25vdGVib29rDQotLS0NCg0KIyMgUHJlcGFyYXRpb24NCllvdSB3aWxsIG5lZWQgdG8gZG93bmxvYWQgcmF3IGRhdGEgZnJvbTogaHR0cHM6Ly9sYW1icmVjaHRzbGFiLnNpdGVzLnZpYi5iZS9lbi9pbW11bmUtYXRsYXMNCg0KVGhlIHBhZ2UgcmVxdWlyZXMgbG9naW4gdG8gZG93bmxvYWQgZGF0YSwgYnV0IGFueSBHb29nbGUgYWNjb3VudCBpcyBnb29kLiBBZnRlciBsb2dpbiwgdGhlcmUgd2lsbCBiZSBtdWx0aXBsZSBsaW5rcyBmb3IgZG93bmxvYWRpbmcuIFRoZSAyMDY5LUFsbGNlbGxzLmNvdW50cy5yZHMgaXMgYXZhaWxhYmxlIGluIHRoZSAiQ2x1c3RlcmluZyBhbGwgY2VsbHMiIHNlY3Rpb24uIFRoZSAyMDc2LU9ubGluZVRNRU0xMDZCLnJkcyBpcyBpbiB0aGUgIlRNRU0xMDZCIiBzZWN0aW9uLg0KDQpgYGB7cn0NCmxpYnJhcnkoU2V1cmF0KQ0KZGF0YSA8LSByZWFkUkRTKCIyMDc2LU9ubGluZVRNRU0xMDZCLnJkcyIpDQpkYXRhQG1ldGEuZGF0YQ0KDQpvYmogPC0gcmVhZFJEUygiMjA2OS1BbGxjZWxscy5jb3VudHMucmRzIikNCm1ldGEuZGF0YSA8LSBkYXRhQG1ldGEuZGF0YVtjb2xuYW1lcyhvYmopLCBdDQpvYmogPC0gQ3JlYXRlU2V1cmF0T2JqZWN0KG9iaiwgbWV0YS5kYXRhID0gbWV0YS5kYXRhKQ0Kb2JqDQpgYGANCmBgYHtyfQ0Kb2JqJFBhdGllbnQgPC0gc3RyaW5ncjo6c3RyX21hdGNoKGNvbG5hbWVzKG9iaiksICJeKC4rKV8iKVssIDJdDQp0YWJsZShvYmokUGF0aWVudCwgb2JqJFBhdGllbnRUeXBlKQ0Kb2JqJFBhdGllbnRUeXBlMiA8LSBvYmokUGF0aWVudFR5cGUNCm9iaiRQYXRpZW50VHlwZTJbb2JqJFBhdGllbnRUeXBlMiA9PSAnSUNVJ10gPC0gJ3NldmVyZScNCm9iaiRQYXRpZW50VHlwZTJbb2JqJFBhdGllbnRUeXBlMiA9PSAnV2FyZCddIDwtICdtaWxkJw0KYGBgDQoNCmBgYHtyfQ0Kb2JqIDwtIE5vcm1hbGl6ZURhdGEob2JqLCB2ZXJib3NlID0gRikNCmBgYA0KDQoNCg0KYGBge3J9DQpsaWJyYXJ5KFNldXJhdCkNCmxpYnJhcnkoY293cGxvdCkNCmxpYnJhcnkoZ3JpZCkNCmxpYnJhcnkoZ3JpZEV4dHJhKQ0KbGlicmFyeShnZ3Bsb3QyKQ0KbGlicmFyeShsYXR0aWNlKQ0KDQoNCm1lZGlhbi5zdGF0IDwtIGZ1bmN0aW9uKHgpew0KICAgb3V0IDwtIHF1YW50aWxlKHgsIHByb2JzID0gYygwLjUpKQ0KICAgbmFtZXMob3V0KSA8LSBjKCJ5bWVkIikNCiAgIHJldHVybihvdXQpIA0KfQ0KDQptZWRpYW4uc3RhdDc1IDwtIGZ1bmN0aW9uKHgpew0KICAgb3V0IDwtIHF1YW50aWxlKHgsIHByb2JzID0gYygwLjc1KSkNCiAgIG5hbWVzKG91dCkgPC0gYygieW1lZCIpDQogICByZXR1cm4ob3V0KSANCn0NCg0KYCVuaSVgPC0gTmVnYXRlKGAlaW4lYCkNCmBgYA0KDQoNCiMjIEdlbmVyYXRlIERFRyB0YWJsZXMNCg0KYGBge3J9DQpkYXRhIDwtIG9iag0KZ29pIDwtIHJlYWQudGFibGUoIm5lZ2F0aXZlLWNhbmRpZGF0ZXMudHh0IikkVjENCiNjbnQgPSAwDQoNCmRhdGEkRG9tYWluX2Rpc2Vhc2VfcGF0aWVudF90eXBlIDwtIHBhc3RlKGRhdGEkRG9tYWluLCBkYXRhJERpc2Vhc2UsIGRhdGEkUGF0aWVudFR5cGUyLCBzZXA9J18nKQ0KZGF0YSREb21haW5fZGlzZWFzZV9wYXRpZW50X3R5cGUgPC0gZmFjdG9yKGRhdGEkRG9tYWluX2Rpc2Vhc2VfcGF0aWVudF90eXBlLCANCiAgICAgICAgICAgICAgICAgICAgICAgICAgICAgICAgICAgICAgICAgIGxldmVscyA9IGMoIkVwaXRoZWxpYWxfY29udHJvbF9taWxkIiwgIkVwaXRoZWxpYWxfY29udHJvbF9zZXZlcmUiLA0KICAgICAgICAgICAgICAgICAgICAgICAgICAgICAgICAgICAgICAgICAgICAgICAgICAgICAiRXBpdGhlbGlhbF9DT1ZJRDE5X21pbGQiLCAiRXBpdGhlbGlhbF9DT1ZJRDE5X3NldmVyZSINCiAgICAgICAgICAgICAgICAgICAgICAgICAgICAgICAgICAgICAgICAgICAgICAgICAgICAgKSkNCklkZW50cyhkYXRhKSA8LSAnRG9tYWluX2Rpc2Vhc2VfcGF0aWVudF90eXBlJw0KDQogICAgbXlfY29tcGFyaXNvbnMgPC0gbGlzdCggYygiRXBpdGhlbGlhbF9jb250cm9sX21pbGQiLCAiRXBpdGhlbGlhbF9jb250cm9sX3NldmVyZSIpLCANCiAgICAgICAgICAgICAgICAgICAgICAgIGMoIkVwaXRoZWxpYWxfY29udHJvbF9zZXZlcmUiLCAiRXBpdGhlbGlhbF9DT1ZJRDE5X21pbGQiKSwgDQogICAgICAgICAgICAgICAgICAgICAgICBjKCJFcGl0aGVsaWFsX0NPVklEMTlfbWlsZCIsICJFcGl0aGVsaWFsX0NPVklEMTlfc2V2ZXJlIiksDQogICAgICAgICAgICAgICAgICAgICAgICBjKCJFcGl0aGVsaWFsX2NvbnRyb2xfbWlsZCIsICJFcGl0aGVsaWFsX0NPVklEMTlfbWlsZCIpLCANCiAgICAgICAgICAgICAgICAgICAgICAgIGMoIkVwaXRoZWxpYWxfY29udHJvbF9zZXZlcmUiLCAiRXBpdGhlbGlhbF9DT1ZJRDE5X3NldmVyZSIpLA0KICAgICAgICAgICAgICAgICAgICAgICAgYygiRXBpdGhlbGlhbF9jb250cm9sX21pbGQiLCAiRXBpdGhlbGlhbF9DT1ZJRDE5X3NldmVyZSIpKQ0KDQpkZiA8LSBkYXRhLmZyYW1lKG5yb3dzID0gZ29pKQ0Kcm93bmFtZXMoZGYpIDwtIGdvaQ0KDQpmb3IgKGNtcCBpbiBteV9jb21wYXJpc29ucykgew0KICBkZltbcGFzdGUwKGNtcFsxXSwgJ192c18nLCBjbXBbMl0sICdfcCcpXV0gPC0gTkENCiAgZGZbW3Bhc3RlMChjbXBbMV0sICdfdnNfJywgY21wWzJdLCAnX3BfYWRqJyldXSA8LSBOQQ0KICBkZltbcGFzdGUwKGNtcFsxXSwgJ192c18nLCBjbXBbMl0sICdfbG9nZmMnKV1dIDwtIE5BDQogIA0KICBtdXR1YWxfZmVhdHVyZXMgPC0gaW50ZXJzZWN0KGdvaSwgcm93bmFtZXMoZGF0YSkpDQogIA0KICBkZWcgPC0gRmluZE1hcmtlcnMoZGF0YSwgY21wWzFdLCBjbXBbMl0sIGZlYXR1cmVzID0gbXV0dWFsX2ZlYXR1cmVzLCBzbG90ID0gImRhdGEiLA0KICAgICAgICAgICAgICAgICAgICAgICAgbWluLmNlbGxzLmdyb3VwID0gMCwgDQogICAgICAgICAgICAgICAgICAgICAgICBtaW4uY2VsbHMuZmVhdHVyZSA9IDAsDQogICAgICAgICAgICAgICAgICAgICAgICBtaW4ucGN0ID0gLTEsDQogICAgICAgICAgICAgICAgICAgICAgICBsb2dmYy50aHJlc2hvbGQgPSAtMSwNCiAgICAgICAgICAgICAgICAgICAgICAgIG9ubHkucG9zID0gRkFMU0UpDQogIA0KICBkZlttdXR1YWxfZmVhdHVyZXMsIHBhc3RlMChjbXBbMV0sICdfdnNfJywgY21wWzJdLCAnX3AnKV0gPC0gZGVnW211dHVhbF9mZWF0dXJlcywgJ3BfdmFsJ10NCiAgZGZbbXV0dWFsX2ZlYXR1cmVzLCBwYXN0ZTAoY21wWzFdLCAnX3ZzXycsIGNtcFsyXSwgJ19wX2FkaicpXSA8LSBkZWdbbXV0dWFsX2ZlYXR1cmVzLCAncF92YWxfYWRqJ10NCiAgZGZbbXV0dWFsX2ZlYXR1cmVzLCBwYXN0ZTAoY21wWzFdLCAnX3ZzXycsIGNtcFsyXSwgJ19sb2dmYycpXSA8LSBkZWdbbXV0dWFsX2ZlYXR1cmVzLCAnYXZnX2xvZzJGQyddDQp9DQoNCndyaXRlLmNzdihkZiwgImVwaXRoZWxpYWwtc2V2ZXJpdHktZGUtbmVnYXRpdmUtdGFyZ2V0cy5jc3YiKQ0KDQpgYGANCg0KYGBge3J9DQpnb2kgPC0gcmVhZC50YWJsZSgicG9zaXRpdmUtY2FuZGlkYXRlcy50eHQiKSRWMQ0KI2NudCA9IDANCg0KZGF0YSREb21haW5fZGlzZWFzZV9wYXRpZW50X3R5cGUgPC0gcGFzdGUoZGF0YSREb21haW4sIGRhdGEkRGlzZWFzZSwgZGF0YSRQYXRpZW50VHlwZTIsIHNlcD0nXycpDQpkYXRhJERvbWFpbl9kaXNlYXNlX3BhdGllbnRfdHlwZSA8LSBmYWN0b3IoZGF0YSREb21haW5fZGlzZWFzZV9wYXRpZW50X3R5cGUsIA0KICAgICAgICAgICAgICAgICAgICAgICAgICAgICAgICAgICAgICAgICAgbGV2ZWxzID0gYygiRXBpdGhlbGlhbF9jb250cm9sX21pbGQiLCAiRXBpdGhlbGlhbF9jb250cm9sX3NldmVyZSIsDQogICAgICAgICAgICAgICAgICAgICAgICAgICAgICAgICAgICAgICAgICAgICAgICAgICAgICJFcGl0aGVsaWFsX0NPVklEMTlfbWlsZCIsICJFcGl0aGVsaWFsX0NPVklEMTlfc2V2ZXJlIg0KICAgICAgICAgICAgICAgICAgICAgICAgICAgICAgICAgICAgICAgICAgICAgICAgICAgICApKQ0KSWRlbnRzKGRhdGEpIDwtICdEb21haW5fZGlzZWFzZV9wYXRpZW50X3R5cGUnDQoNCiAgICBteV9jb21wYXJpc29ucyA8LSBsaXN0KCBjKCJFcGl0aGVsaWFsX2NvbnRyb2xfbWlsZCIsICJFcGl0aGVsaWFsX2NvbnRyb2xfc2V2ZXJlIiksIA0KICAgICAgICAgICAgICAgICAgICAgICAgYygiRXBpdGhlbGlhbF9jb250cm9sX3NldmVyZSIsICJFcGl0aGVsaWFsX0NPVklEMTlfbWlsZCIpLCANCiAgICAgICAgICAgICAgICAgICAgICAgIGMoIkVwaXRoZWxpYWxfQ09WSUQxOV9taWxkIiwgIkVwaXRoZWxpYWxfQ09WSUQxOV9zZXZlcmUiKSwNCiAgICAgICAgICAgICAgICAgICAgICAgIGMoIkVwaXRoZWxpYWxfY29udHJvbF9taWxkIiwgIkVwaXRoZWxpYWxfQ09WSUQxOV9taWxkIiksIA0KICAgICAgICAgICAgICAgICAgICAgICAgYygiRXBpdGhlbGlhbF9jb250cm9sX3NldmVyZSIsICJFcGl0aGVsaWFsX0NPVklEMTlfc2V2ZXJlIiksDQogICAgICAgICAgICAgICAgICAgICAgICBjKCJFcGl0aGVsaWFsX2NvbnRyb2xfbWlsZCIsICJFcGl0aGVsaWFsX0NPVklEMTlfc2V2ZXJlIikpDQoNCmRmIDwtIGRhdGEuZnJhbWUobnJvd3MgPSBnb2kpDQpyb3duYW1lcyhkZikgPC0gZ29pDQoNCmZvciAoY21wIGluIG15X2NvbXBhcmlzb25zKSB7DQogIGRmW1twYXN0ZTAoY21wWzFdLCAnX3ZzXycsIGNtcFsyXSwgJ19wJyldXSA8LSBOQQ0KICBkZltbcGFzdGUwKGNtcFsxXSwgJ192c18nLCBjbXBbMl0sICdfcF9hZGonKV1dIDwtIE5BDQogIGRmW1twYXN0ZTAoY21wWzFdLCAnX3ZzXycsIGNtcFsyXSwgJ19sb2dmYycpXV0gPC0gTkENCiAgDQogIG11dHVhbF9mZWF0dXJlcyA8LSBpbnRlcnNlY3QoZ29pLCByb3duYW1lcyhkYXRhKSkNCiAgDQogIGRlZyA8LSBGaW5kTWFya2VycyhkYXRhLCBjbXBbMV0sIGNtcFsyXSwgZmVhdHVyZXMgPSBtdXR1YWxfZmVhdHVyZXMsIHNsb3QgPSAiZGF0YSIsDQogICAgICAgICAgICAgICAgICAgICAgICBtaW4uY2VsbHMuZ3JvdXAgPSAwLCANCiAgICAgICAgICAgICAgICAgICAgICAgIG1pbi5jZWxscy5mZWF0dXJlID0gMCwNCiAgICAgICAgICAgICAgICAgICAgICAgIG1pbi5wY3QgPSAtMSwNCiAgICAgICAgICAgICAgICAgICAgICAgIGxvZ2ZjLnRocmVzaG9sZCA9IC0xLA0KICAgICAgICAgICAgICAgICAgICAgICAgb25seS5wb3MgPSBGQUxTRSkNCiAgDQogIGRmW211dHVhbF9mZWF0dXJlcywgcGFzdGUwKGNtcFsxXSwgJ192c18nLCBjbXBbMl0sICdfcCcpXSA8LSBkZWdbbXV0dWFsX2ZlYXR1cmVzLCAncF92YWwnXQ0KICBkZlttdXR1YWxfZmVhdHVyZXMsIHBhc3RlMChjbXBbMV0sICdfdnNfJywgY21wWzJdLCAnX3BfYWRqJyldIDwtIGRlZ1ttdXR1YWxfZmVhdHVyZXMsICdwX3ZhbF9hZGonXQ0KICBkZlttdXR1YWxfZmVhdHVyZXMsIHBhc3RlMChjbXBbMV0sICdfdnNfJywgY21wWzJdLCAnX2xvZ2ZjJyldIDwtIGRlZ1ttdXR1YWxfZmVhdHVyZXMsICdhdmdfbG9nMkZDJ10NCn0NCg0Kd3JpdGUuY3N2KGRmLCAiZXBpdGhlbGlhbC1zZXZlcml0eS1kZS1wb3NpdGl2ZS10YXJnZXRzLmNzdiIpDQpgYGANCg==
