# Supplementary material for: Integrated multi-omics analyses identify anti-viral host factors and pathways controlling SARS-CoV-2 infection
Source: Nat Commun. 2024 Jan 2;15:109. doi: 10.1038/s41467-023-44175-1 (PMC10761986; doi:10.1038/s41467-023-44175-1)
Supplement: Supplementary file 10 — Reporting Summary [file 41467_2023_44175_MOESM10_ESM.pdf]

nature portfolio

Corresponding author(s): Weiyi Peng

Last updated by author(s): Oct 17, 2023

Reporting Summary

Nature Portfolio wishes to improve the reproducibility of the work that we publish. This form provides structure for consistency and transparency in reporting. For further information on Nature Portfolio policies, see our Editorial Policies and the Editorial Policy Checklist.

Statistics

For all statistical analyses, confirm that the following items are present in the figure legend, table legend, main text, or Methods section.

n/a	Confirmed	
☐	☒	The exact sample size (n) for each experimental group/condition, given as a discrete number and unit of measurement
☐	☒	A statement on whether measurements were taken from distinct samples or whether the same sample was measured repeatedly
☐	☒	The statistical test(s) used AND whether they are one- or two-sided<br>*Only common tests should be described solely by name; describe more complex techniques in the Methods section.*
☐	☒	A description of all covariates tested
☐	☒	A description of any assumptions or corrections, such as tests of normality and adjustment for multiple comparisons
☐	☒	A full description of the statistical parameters including central tendency (e.g. means) or other basic estimates (e.g. regression coefficient) AND variation (e.g. standard deviation) or associated estimates of uncertainty (e.g. confidence intervals)
☐	☒	For null hypothesis testing, the test statistic (e.g. F, t, r) with confidence intervals, effect sizes, degrees of freedom and P value noted<br>*Give P values as exact values whenever suitable.*
☒	☐	For Bayesian analysis, information on the choice of priors and Markov chain Monte Carlo settings
☒	☐	For hierarchical and complex designs, identification of the appropriate level for tests and full reporting of outcomes
☐	☒	Estimates of effect sizes (e.g. Cohen's d, Pearson's r), indicating how they were calculated

Our web collection on statistics for biologists contains articles on many of the points above.

Software and code

Policy information about availability of computer code

Data collection	The quality and concentration of all PCR products were determined by the Qubit ssDNA high sensitivity assay kit (#Q10212; ThermoFisher) and the bioanalyzer High Sensitivity DNA Kit (#5067-4626 2100; Agilent, Santa Clara, CA) respectively. Samples were then sequenced by Illumina NextSeq 500 at the MD Anderson Cancer Center Advanced Technology Genomics Core. RNA-Seq libraries were prepared and subjected to paired-end sequencing by NovoGene. ABI 7900 Real-Time PCR System was used for qPCR. The intensity of protein bands was detected the ChemiDoc Imaging System(Bio-Rad). The COVID-19 GWAS meta-analyses results (release 6) for “Hospitalized covid vs. population” and “Very severe respiratory confirmed covid vs. population” were downloaded from the COVID19 Host Genetics Initiative (https://www.covid19hg.org/). The protein-protein interaction (PPI) data were downloaded from the BioGRID database (Release 4.4.205). The host-viral protein-RNA interactome (RPI) datasets were extracted from published literatures.
Data analysis	Data analysis was performed using GraphPad Prism v8, Image Lab software, MAGeCK (v0.5.9.4), R (4.1.3), Cytoscape (3.8.2), PhenoGram Plot, QIAGEN Ingenuity Pathway Analysis (Version: 90348151)

For manuscripts utilizing custom algorithms or software that are central to the research but not yet described in published literature, software must be made available to editors and reviewers. We strongly encourage code deposition in a community repository (e.g. GitHub). See the Nature Portfolio guidelines for submitting code & software for further information.

Data

Policy information about availability of data

All manuscripts must include a data availability statement. This statement should provide the following information, where applicable:

- Accession codes, unique identifiers, or web links for publicly available datasets
- A description of any restrictions on data availability
- For clinical datasets or third party data, please ensure that the statement adheres to our policy

The data that support the findings of this study will be provided by corresponding authors upon reasonable request. All raw sequencing data will be deposited in the GEO (GSE209750) upon acceptance of the manuscript. Please use the token:shefgyqwljkxdav to access the dataset.

Human research participants

Policy information about studies involving human research participants and Sex and Gender in Research.

Reporting on sex and gender	NA.
Population characteristics	NA.
Recruitment	NA.
Ethics oversight	NA.

Note that full information on the approval of the study protocol must also be provided in the manuscript.

Field-specific reporting

Please select the one below that is the best fit for your research. If you are not sure, read the appropriate sections before making your selection.

☒ Life sciences ☐ Behavioural & social sciences ☐ Ecological, evolutionary & environmental sciences

For a reference copy of the document with all sections, see nature.com/documents/nr-reporting-summary-flat.pdf

Life sciences study design

All studies must disclose on these points even when the disclosure is negative.

Sample size	For all cell based experiments, n>=3 biologically independent replicates derived from different wells per group were used. These sample size settings are often reported in previous publications to control the technical variations and get confident data.
Data exclusions	NA.
Replication	All experiments were performed at least two independent biological repeats and/or with sufficient cells per group. All the data from different replications are consistent.
Randomization	All experiments are conducted in a blind, random manner where it is possible.
Blinding	All experiments are conducted in a blind, random manner where it is possible.

Reporting for specific materials, systems and methods

We require information from authors about some types of materials, experimental systems and methods used in many studies. Here, indicate whether each material, system or method listed is relevant to your study. If you are not sure if a list item applies to your research, read the appropriate section before selecting a response.

Materials & experimental systems

n/a	Involved in the study
☐	☒ Antibodies
☐	☒ Eukaryotic cell lines
☒	☐ Palaeontology and archaeology
☒	☐ Animals and other organisms
☒	☐ Clinical data
☒	☐ Dual use research of concern

Methods

n/a	Involved in the study
☒	☐ ChIP-seq
☐	☒ Flow cytometry
☒	☐ MRI-based neuroimaging

Antibodies

Antibodies used: The antibodies targeting beta-actin (8H10D10, #3700), SERPINE1 (E3I5H, #49536) was purchased from the Cell Signaling Technology (Danvers, MA), human ACE2 (AF933 and FAB9332R) were purchased from R&D Systems, DAZAP2 (G-4, sc-515182), GAPDH (0411, sc-47724) were purchased from Santa Cruz Biotechnology (Dallas, TX), KLF5 (21017-1-AP) was purchased from Proteintech Group (Rosemont, IL) and the monoclonal ANTI-FLAG antibody (M2, #F3165) was purchased from MilliporeSigma. HRP conjugated secondary antibodies anti-rabbit IgG (#7047) and anti-mouse IgG (#7076) were purchased from the Cell Signaling Technology (Danvers, MA).

Validation: All the antibodies used in this study are commercial available, and were validated by the manufacturer (available on their websites)

Eukaryotic cell lines

Policy information about cell lines and Sex and Gender in Research

Cell line source(s): A549, H2023, Calu-3, and HEK293T cell lines were obtained from the American Type Culture Collection (ATCC, Bethesda, MD).

Authentication: All cell lines were authenticated by short tandem repeat fingerprinting or the expression of tagged markers used for genetic modification.

Mycoplasma contamination: The mycoplasma detection kit (#13100-01, SouthernBiotech, Birmingham, AL) was used to routinely monitor for mycoplasma contamination of cultured cells.

Commonly misidentified lines (See ICLAC register): No commonly mis-identified cell lines were used in this study.

Flow Cytometry

Plots

Confirm that:

☒ The axis labels state the marker and fluorochrome used (e.g. CD4-FITC).

☒ The axis scales are clearly visible. Include numbers along axes only for bottom left plot of group (a 'group' is an analysis of identical markers).

☒ All plots are contour plots with outliers or pseudocolor plots.

☒ A numerical value for number of cells or percentage (with statistics) is provided.

Methodology

Sample preparation: Cells were trypsinized and washed with pre-chilled PBS and then resuspended into PBS with 2% FBS. Cells were labeled with ACE2 antibody conjugated with Alexa Fluor® 647 for 30 mins at 4 degree and then washed by pre-chilled PBS. Cells stained with isotype IgG were used as the negative control.

Instrument: BD LSRFortessa™ X-20 Cell Analyzer

Software: FLOWJo_V10.8.1

Cell population abundance: No sorting steps.

Gating strategy: Cells were gated by FSC/SSC to remove the debris and then performed the doublet discrimination by using SSC-H and SSC-A. GFP and APC signals were detected in the single cell population.

☒ Tick this box to confirm that a figure exemplifying the gating strategy is provided in the Supplementary Information.
