# Supplementary figures and images for: Integrated multi-omics analyses identify anti-viral host factors and pathways controlling SARS-CoV-2 infection
Source: Nat Commun. 2024 Jan 2;15:109. doi: 10.1038/s41467-023-44175-1 (PMC10761986; doi:10.1038/s41467-023-44175-1)

Figure 5

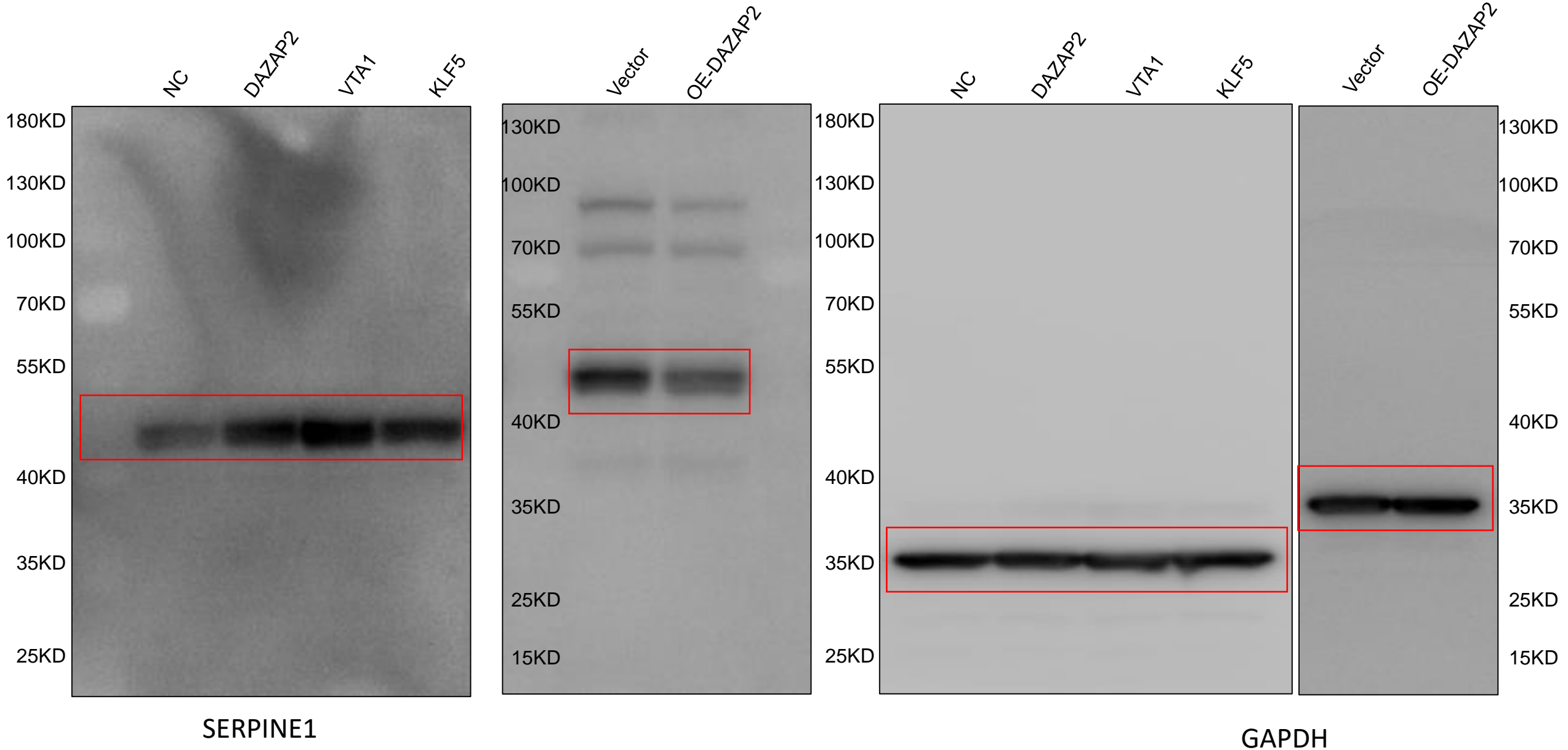

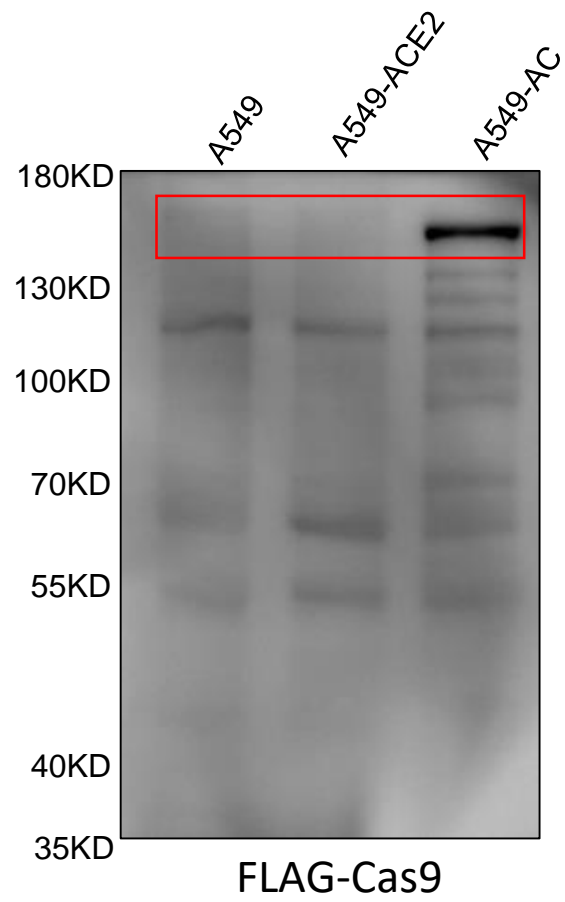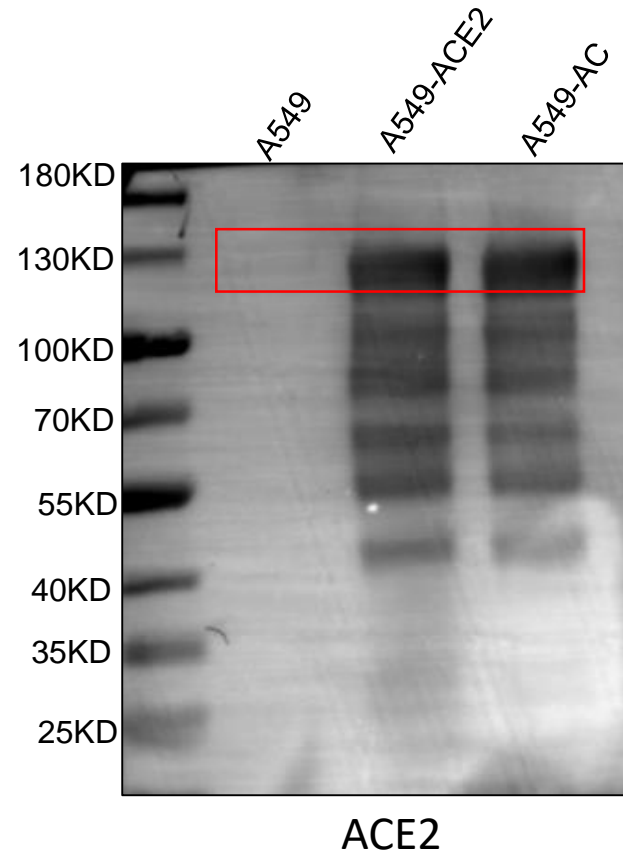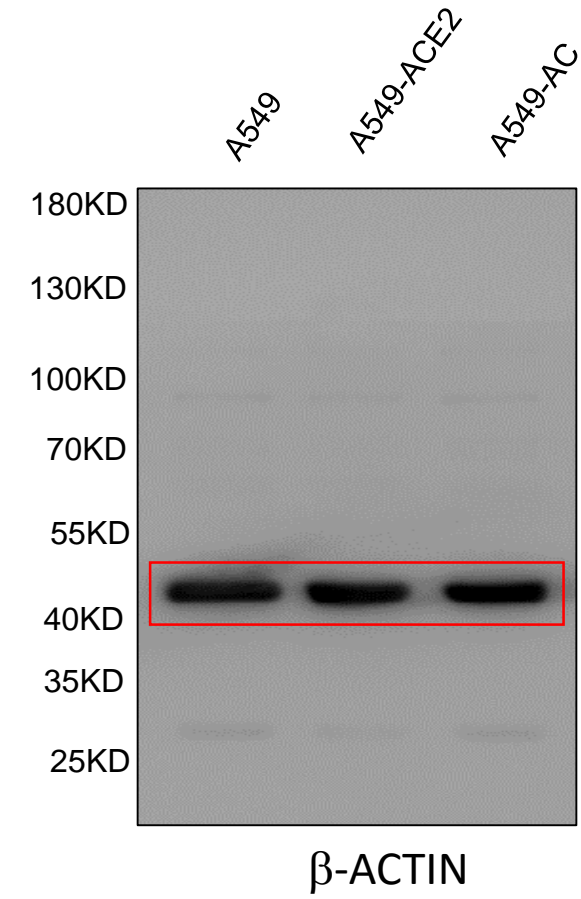

Supplementary Figure 6

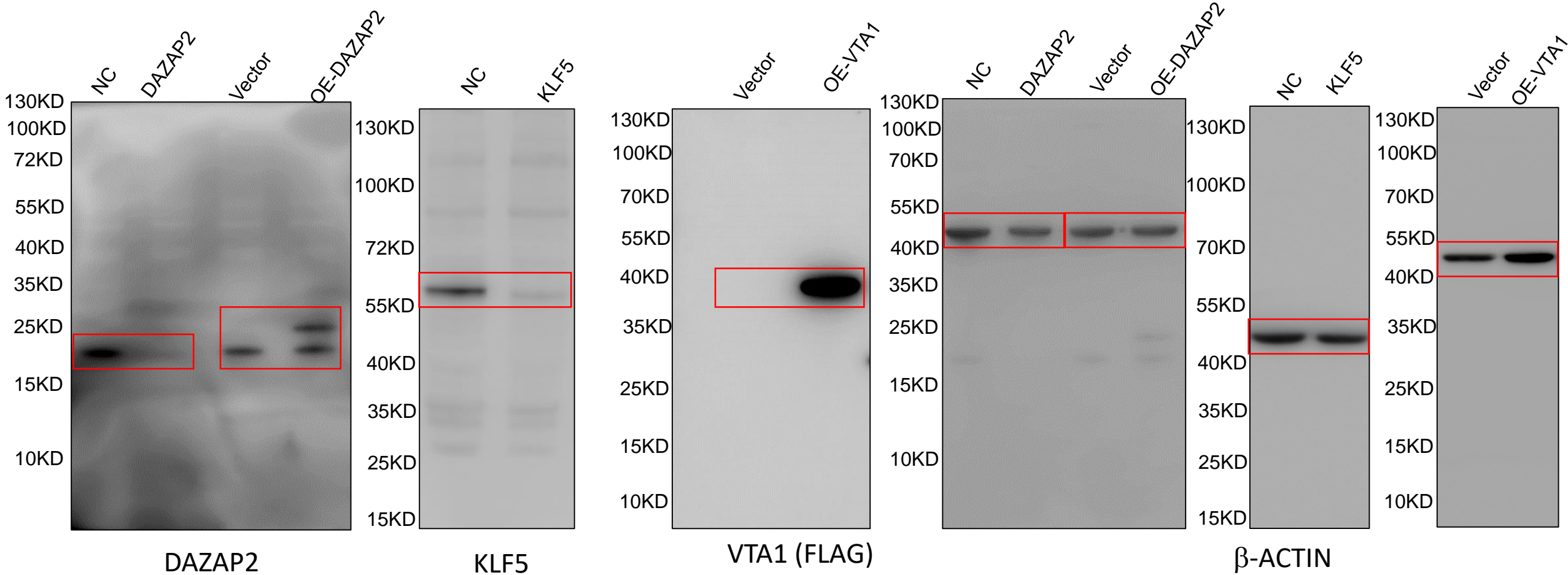

Supplement: Supplementary file 11 — Source Data [file 41467_2023_44175_MOESM11_ESM.zip › 382528_2_supp_8255388_s3f5hp_convrt.pdf]
